# Feasibility of measles and rubella vaccination programmes for disease elimination: a modelling study

**DOI:** 10.1016/S2214-109X(22)00335-7

**Published:** 2022-09-13

**Authors:** Amy K Winter, Brian Lambert, Daniel Klein, Petra Klepac, Timos Papadopoulos, Shaun Truelove, Colleen Burgess, Heather Santos, Jennifer K Knapp, Susan E Reef, Lidia K Kayembe, Stephanie Shendale, Katrina Kretsinger, Justin Lessler, Emilia Vynnycky, Kevin McCarthy, Matthew Ferrari, Mark Jit

**Affiliations:** a Department of Epidemiology and Biostatics and Center for the Ecology of Infectious Diseases, University of Georgia, Athens, GA, USA; b Department of Biology, Pennsylvania State University, State College, PA, USA; c Institute for Disease Modeling, Global Health Division, Bill & Melinda Gates Foundation, Seattle, WA, USA; d Department of Infectious Disease Epidemiology, Faculty of Epidemiology and Population Health, London School of Hygiene & Tropical Medicine, London, UK; e TB Modelling Group, Centre for Mathematical Modelling of Infectious Diseases, London School of Hygiene & Tropical Medicine, London, UK; f Department of Statistics Modelling and Economics, UK Health Security Agency, London, UK; g Institute of Sound and Vibration Research, University of Southampton, Southampton, UK; h Department of International Health, Johns Hopkins Bloomberg School of Public Health, Baltimore, MD, USA; i Ramboll Health Sciences, Copenhagen, Denmark; j Global Immunization Division, Centers for Disease Control and Prevention, Atlanta, GA, USA; k Department of Immunization, Vaccines and Biologicals, WHO, Geneva, Switzerland; l Department of Epidemiology, Gillings School of Global Public Health and Carolina Population Center, University of North Carolina, Chapel Hill, NC, USA

## Abstract

**Background:**

Marked reductions in the incidence of measles and rubella have been observed since the widespread use of the measles and rubella vaccines. Although no global goal for measles eradication has been established, all six WHO regions have set measles elimination targets. However, a gap remains between current control levels and elimination targets, as shown by large measles outbreaks between 2017 and 2019. We aimed to model the potential for measles and rubella elimination globally to inform a WHO report to the 73rd World Health Assembly on the feasibility of measles and rubella eradication.

**Methods:**

In this study, we modelled the probability of measles and rubella elimination between 2020 and 2100 under different vaccination scenarios in 93 countries of interest. We evaluated measles and rubella burden and elimination across two national transmission models each (Dynamic Measles Immunisation Calculation Engine [DynaMICE], Pennsylvania State University [PSU], Johns Hopkins University, and Public Health England models), and one subnational measles transmission model (Institute for Disease Modeling model). The vaccination scenarios included a so-called business as usual approach, which continues present vaccination coverage, and an intensified investment approach, which increases coverage into the future. The annual numbers of infections projected by each model, country, and vaccination scenario were used to explore if, when, and for how long the infections would be below a threshold for elimination.

**Findings:**

The intensified investment scenario led to large reductions in measles and rubella incidence and burden. Rubella elimination is likely to be achievable in all countries and measles elimination is likely in some countries, but not all. The PSU and DynaMICE national measles models estimated that by 2050, the probability of elimination would exceed 75% in 14 (16%) and 36 (39%) of 93 modelled countries, respectively. The subnational model of measles transmission highlighted inequity in routine coverage as a likely driver of the continuance of endemic measles transmission in a subset of countries.

**Interpretation:**

To reach regional elimination goals, it will be necessary to innovate vaccination strategies and technologies that increase spatial equity of routine vaccination, in addition to investing in existing surveillance and outbreak response programmes.

**Funding:**

WHO, Gavi, the Vaccine Alliance, US Centers for Disease Control and Prevention, and the Bill & Melinda Gates Foundation.

## Introduction

Global increases in measles and rubella vaccine coverage have resulted in substantial reductions in the number of infections and the burden of disease. Between 2000 and 2019, the incidence of measles decreased by 62% and 25·5 million deaths have been averted.^1^ As of January, 2021, measles had been eliminated in 81 countries. Rubella vaccination has been introduced in 173 of 194 WHO member countries,^2^ and as of January, 2021, its elimination had been verified in 93 countries.^3^ The last case of endemic rubella was reported in the WHO Region of the Americas in 2009, and in 2015, the Region was verified as free of endemic rubella and congenital rubella syndrome.^4^ However, between 2017 and 2019, measles cases rebounded in all regions of the world; the global number of measles cases increased by 556% between 2016 and 2019, including large outbreaks in Ukraine, Madagascar, and the Democratic Republic of the Congo.^1^ As a result of endemic transmission of measles in Venezuela and Brazil, elimination was not maintained in the WHO Region of the Americas.^1^ Despite sustained rubella-free and measles-free status in many countries, the goal set by the Measles and Rubella Initiative to eliminate measles and rubella in at least five WHO regions by 2020 has not been met.^5^, ^6^ Furthermore, in 2020, lower routine vaccination coverage and postponement of vaccination campaigns due to the COVID-19 pandemic has left many countries susceptible to future outbreaks.^7^, ^8^

**Research in context**

**Evidence before this study**

We searched the title field in the PubMed database on June 16, 2021, without language or date restrictions for all published articles using the search terms “(measles OR rubella) AND (elimination OR eradication)”. Our search yielded 672 records. We evaluated the titles and abstracts of all publications and identified those most relevant to the feasibility of global eradication of measles or rubella. The articles directly addressed goals and challenges of, or progress and strategies towards, measles and rubella elimination or eradication. Eradication of measles is biologically feasible and the programmatic and operational feasibility of measles and rubella elimination has been demonstrated in the WHO Region of the Americas. Progress towards measles elimination has slowed or reversed in the since 2017 following outbreaks in 2018–19 and disruptions to vaccination programmes because of the COVID-19 pandemic. Our comprehensive analysis is the first to evaluate the programmatic feasibility of measles and rubella elimination based on prominent vaccine strategies used in the WHO Region of the Americas (routine vaccination and vaccination campaigns).

**Added value of this study**

We found that intensified scale-up of vaccination strategy coverage in the WHO Region of the Americas could markedly decrease the measles burden and would be likely to eliminate rubella in all 93 countries of interest. However, it might not be enough to reach and maintain measles elimination in all countries, specifically if the cessation of vaccination campaigns is the goal.

**Implications of all the available evidence**

The programmatic feasibility of measles and rubella global eradication will rely on novel vaccination strategies and technologies that address and mitigate inequities in vaccination coverage. Additionally, modelling that accounts for improved surveillance, outbreak response, and revised criteria for ceasing vaccination campaigns would improve understanding of the effort needed to reach elimination in countries where measles transmission is hardest to control. Beyond these considerations, the remaining challenges for measles and rubella eradication will be political and financial.

Measles and rubella are ideal candidates for eradication for a number of reasons.^9^ All six WHO Regions have set regional measles elimination goals and four have rubella elimination goals. However, no global measles or rubella eradication goal has been declared. At the 70th World Health Assembly held in May, 2017, the WHO Director-General was requested to report back in 3 years “on the epidemiological aspects and feasibility of, and potential resource requirements for, measles and rubella eradication”. To address this request, a Feasibility Assessment of Measles and Rubella Eradication was conducted, reported, and published.^10^ One component of the assessment's objectives was to model four vaccination strategies to evaluate the theoretical feasibility of eradication of the two pathogens.

Here, we report the results of the modelling component of the assessment, in which we aimed to evaluate the probability of measles and rubella elimination in 93 countries of interest, focusing on two vaccination scenarios. This work was the joint effort of the WHO Strategic Advisory Group of Experts Measles and Rubella Working Group and the US Centers for Disease Control and Prevention, together with five modelling groups.

## Methods

### Overview

Using disease transmission models, we explored the potential for measles and rubella elimination in 93 countries under two vaccination scenarios. Vaccination scenarios were based on historical measles and rubella vaccination coverage data for both routine immunisation and supplemental immunisation activities (SIAs) obtained from the WHO Immunization dashboard, with future coverage projected using different methods and assumptions for estimating long-term trends. The models projected the distributions of expected numbers of measles or rubella infections annually. These distributions were then analysed to understand the impact of each vaccination scenario in each country on health outcomes (ie, measles deaths or congenital rubella syndrome cases) and the likelihood of achieving and maintaining measles or rubella elimination.

### Vaccination scenarios

The vaccination scenarios relied on the two prominent vaccine delivery mechanisms: routine vaccination via childhood immunisation schedules, and intermittent vaccination campaigns that target large age groups to vaccinate quickly (known as SIAs). Two vaccination scenarios were developed to represent a set of possibilities for constant routine and SIA vaccination coverage (so-called business as usual scenario) and optimally improving routine and SIA vaccination coverage (so-called intensified investment scenario) into the future. SIAs are intended to supplement routine programmes until routine coverage is high enough that campaigns are no longer necessary; therefore, SIA frequency additionally differs by the vaccination scenario. SIAs occurring between 2018 and 2100 in the business as usual scenario were based on a documented history of national measles SIAs between 2000 and 2017, whereas SIAs were more frequent in the intensified investment scenario. For countries that clearly stated they do not plan to continue with large vaccination campaigns, or the opinion of regional subject matter experts was to discontinue large vaccination campaigns, SIAs were discontinued in both scenarios. For the remaining countries, SIAs continued indefinitely in the business as usual scenario but ceased in the intensified investment scenario once control criteria were met. Coverage, vaccine introductions, and campaign frequencies for each vaccination scenario were country-specific and year-specific. Further information on the vaccination scenarios is provided in table 1 and the appendix (pp 4–6).

**Table 1:** Vaccination scenarios

	**MCV1**	**MCV2**	**Rubella-containing vaccine**	**SIAs**		
				Frequency	Coverage	Cessation criteria
Business as usual scenario	Maintain 2017 coverage	Maintain 2017 coverage, no new introductions beyond 2018	Maintain 2017 coverage, no new introductions beyond 2018	Measles campaigns conducted between 2000 and 2017 were used to calculate an SIA interval	Mean age-specific coverages for all campaigns conducted between 2000 and 2017 by country among children aged <5 years	None, if SIAs occurred they continued indefinitely
Intensified investment scenario	For countries that had not eliminated measles or reached 95% MCV1 coverage by 2016, coverage increases at a global median compound rate of 4·4% to up to 99%*; for countries that had eliminated measles and exceeded 90% MCV1 coverage, or reached 95% by 2016, coverage remained constant	Country-specific introduction between 2018 and 2024 (regional SMEs); from 2017 onward, MCV2 coverage was set to the maximum between three options (2 percentage points below the value of MCV1 for that year, the 2016 coverage, or value estimated via natural logarithmic function); for countries that had not introduced MCV2 by 2017, MCV2 coverage at the year of introduction was set to a percentage difference of MCV1 coverage based on World Bank income level-specified differences between MCV1 and MCV2	Country-specific introduction between 2018 and 2024 (regional SMEs); first dose of rubella-containing vaccine coverage equal to MCV1 coverage; second dose of rubella-containing vaccine coverage equal to MCV2 coverage	Based on accrual of unvaccinated individuals since previous SIA (per MCV1 and MCV2 activities) in which SIAs should be conducted when the number of susceptible children is 75% of the size of one birth cohort, or every 4th year, whichever occurs first	Where post-campaign survey results were available since 2016, those were used; administrative coverage was used for SIAs between 2017 and 2019 only if they were lower than the average age-specific coverage estimates in the business as usual scenario; SIA coverage was increased by 10% of the incremental difference between the previous SIA coverage estimate and 100%, then following 2020 coverage increased in two equal increments to 95% coverage; SIAs occurring after the introduction of rubella-containing vaccine are aimed at children aged <5 years	Rubella-containing vaccine introduction with an introduction campaign and one follow-up campaign, >5 years after MCV2, when the number of susceptible children in a 10-year period is smaller than the size of one birth cohort

Vaccination scenarios were based on historical measles and rubella vaccination coverage data for both routine immunisation and SIAs obtained from the WHO Immunization dashboard. Two vaccination scenarios were used, with differing levels of coverage, introduction dates, and timing for routine and supplemental measles and rubella immunisation efforts. For each scenario, country-specific and year-specific estimates and practices for each of the 93 countries from 2018 to 2100 are detailed in the appendix (p 55). These estimates include MCV1 and MCV2, rubella-containing vaccine doses (implemented within the measles-containing vaccines after introduction), and SIAs. Where necessary, SMEs informed the vaccination scenarios. MCV1=first dose of measles-containing vaccine. MCV2=two doses of measles-containing vaccine. SIAs=supplemental immunisation activities. SMEs=subject matter experts.

* Global median compound rates of coverage are included in the appendix (p 4).

### Transmission models

The vaccination scenarios were evaluated within the context of two national models^11^, ^12^ and one subnational model for measles transmission, and two national models for rubella transmission.^13^, ^14^, ^15^ The four national-level models have previously been used to generate future projections of the impact of investments made by Gavi, the Vaccine Alliance as part of the Vaccine Impact Modelling Consortium; they include Dynamic Measles Immunisation Calculation Engine (DynaMICE; developed by the London School of Hygiene & Tropical Medicine), Pennsylvania State University (PSU), Johns Hopkins University (JHU), and Public Health England (PHE) models. The 93 low-income, lower-middle income, and upper-middle income countries with the highest measles and rubella burden and incidence globally were selected, accounting for 91% of global measles cases and 90% of global rubella cases in 2019 (appendix p 3).^16^ The subnational Institute for Disease Modeling model simulated measles dynamics in a single country (Nigeria), providing spatial granularity that complements the national models.

Each transmission model captures both the direct and indirect (herd) impact of vaccination, with uncertainty originating from input parameter uncertainty distributions and, in some cases, first-order uncertainty (ie, randomness in the model processes). Each model was run for 200 stochastic simulations for each vaccination scenario and country from 1980 to 2100 (to 2050 for the subnational model). All models use some form of compartmental structure, whereby populations or individuals move between epidemiological classes. All models account for maternal immunity, vaccine efficacy, and assume lifelong immunity following infection with or vaccination against measles or rubella. Demographic and vaccination data were standardised across models of the same pathogen. Demographic data (population size, crude birth rates, and age-specific death rates) were supplied by the Vaccine Impact Modelling Consortium based on United Nations World Population Prospects. Vaccination data were defined by the vaccination scenarios. In the national models, all vaccine doses were assumed to be administered uniformly and randomly across the population, with no correlation between doses. The subnational model considers alternative scenarios exploring the impact of correlation between doses. Details of each model are summarised in table 2 and the appendix (pp 6–12).

**Table 2:** Key assumptions of measles and rubella transmission models

	**Model structure**	**Seasonality**	**Age and spatial mixing patterns**	**Measles case fatality rate**	Risk of congenital rubella syndrome*	**Vaccine effectiveness**	**Case importation**
DynaMICE national measles model	Mechanistic, deterministic, age-stratified, MSIRV†	Yes	Age-dependent per POLYMOD study^17^ (Great Britain)	Country-specific^18^	NA	First dose 85% effective when given at 9 months, 95% effective when given at 12 months; second dose 98%	No
PSU national measles model	Semi-mechanistic, stochastic MSIR† fitted to observed cases with Kalman filter	No	NA	Country-specific	NA	First dose 84% at 9 months, 93% at 12 months; second dose 99%; SIAs 99%	Random variation in annual attack rate
IDM measles subnational model	Agent-based stochastic metapopulation MSEIR† for each local government area of Nigeria	Yes	Age-dependence fit to incidence data; spatial mixing	4% in children aged <5 years, 2% in children aged ≥5 years	NA	Dependence on age and maternal protection status fit to meta-analysis;^19^ First dose 90% at mean age 9 months (SD 1); second dose 95% at mean age 12 months (SD 1·5); SIAs 95%	Stochastic importation of single cases at constant average rate
JHU national rubella model	Mechanistic, discrete time, stochastic, age-stratified, MSIRV†	Yes	Age-dependent (1 year age groups)^20^	NA	0·59	First dose is age-specific, increasing from 74% at age 6 months to 97% at age 12 months; second dose 97%; SIAs 97%	Stochastic importation of cases scaled by population size
PHE national rubella model	Mechanistic, continuous time, deterministic, age-stratified, MSEIRV†	No	Age-dependent (<13 years and ≥13 years age groups)	NA	0·65	All doses 95%	0·001% of country population susceptible to rubella infection

DynaMICE=Dynamic Measles Immunisation Calculation Engine. NA=not applicable. PSU=Pennsylvania State University. SIA=supplemental immunisation activities. IDM=Institute for Disease Modeling. JHU=Johns Hopkins University. PHE=Public Health England.

* Probability of a pregnant woman infected with rubella in the first 16 weeks of pregnancy giving live birth to a child with congenital rubella syndrome.

† Each model had a different model structure whereby the population was divided into a combination of epidemiological classifications of M (maternally immune), S (susceptible), E (exposed; infected but not yet infectious), I (infected), R (recovered; immune via natural infection), and V (vaccinated; immune via successful vaccination).

### Output analysis

The annual numbers of infections projected by each model, country, and vaccination scenario were used to explore if, when, and for how long the infections would be below a threshold for elimination. Because the models were continuous and infected individuals were periodically introduced as importations in four of the five models, true elimination (ie, sustained periods with no measles or rubella infections in the simulation) did not occur in these models, although short periods of time with zero cases could occur. As such, we defined elimination as an annual incidence of five infections per million people or fewer, although in practice we view the elimination threshold more as a necessary condition for elimination during which transmission would be unstable and likely to be interrupted in the absence of continued case importation. The threshold was conservatively based on five infections per million rather than five reported cases per million, which was maintained from 2003 until elimination in all countries in the Region of the Americas, with the exception of Canada (in 2011 and 2014–15) and Ecuador (in 2011). We explored the timing of achieving the threshold in each country under each vaccination scenario. We also explored the duration of continuous-time periods spent below the threshold to differentiate between temporary low-incidence years (eg, in years following large outbreaks controlled by highly effective SIAs) versus long-term maintenance of incidence below the threshold for multiple years (ie, more robust achievement of near-elimination conditions).

### Role of the funding source

The funders of the study were involved in study design, data collection and data analysis, data interpretation, and writing of the report.

## Results

**Figure 1:**
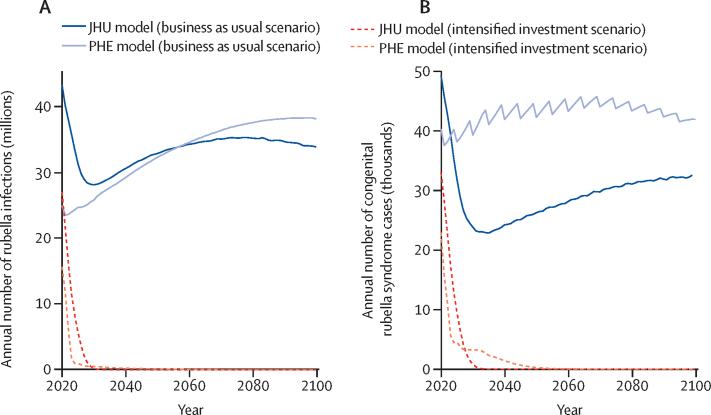
Rubella burden Time series of the annual aggregate number of rubella infections (A) and congenital rubella syndrome cases (B) across 93 countries based on JHU and PHE models under business as usual and intensified investment vaccination scenarios; the line for each model and scenario (ie, colour and line type) represents the median across 200 stochastic runs. JHU=Johns Hopkins University. PHE=Public Health England.

In the business as usual vaccination scenario, the burden of rubella was projected to remain high between 2020 and 2100 (figure 1A, B; appendix p 13). Since most countries had introduced rubella-containing vaccines by 2017 (during the historical period of the models), many were projected to achieve elimination by 2020 (56 countries modelled in the JHU model and four countries modelled in the PHE model; appendix p 14). All countries projected to achieve rubella elimination by 2020 reported less than five rubella cases per million people to WHO between 2017 and 2020 (appendix p 15). However, in this scenario, 23 countries did not introduce rubella-containing vaccines (appendix pp 16–17) and would predominantly drive the number of rubella infections, congenital rubella syndrome cases, and congenital rubella syndrome deaths (appendix pp 18–20).

**Figure 2:**
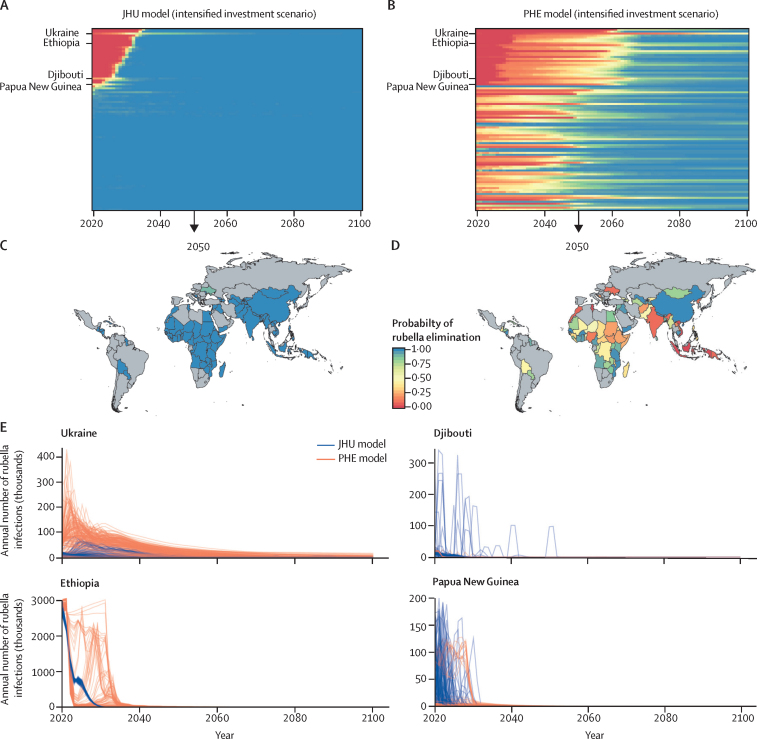
Models of rubella elimination Time series of probability of rubella elimination by country (rows) between 2020 and 2100 for the JHU (A) and PHE (B) models. The probability of rubella elimination at 2050 by country for JHU (C) and PHE (D) models. The probability of achieving the elimination threshold of no more than five rubella infections per million people is shown as a proportion of 200 stochastic runs that would reach the threshold in the intensified investment vaccination scenario. (E) Time series of incident rubella infections for Ukraine, Ethiopia, Djibouti, and Papua New Guinea across 200 stochastic runs for JHU and PHE models; each line represents a different stochastic simulation. JHU=Johns Hopkins University. PHE=Public Health England.

In the intensified investment scenario, wherein rubella-containing vaccines are introduced in all countries and rubella-containing vaccine coverage increases as specified, the total number of rubella infections and congenital rubella syndrome cases was projected to reduce substantially between 2020 and 2100 (figure 1A, B; appendix pp 18–20), and reaching the criteria for rubella elimination would be possible and probable in all countries (figure 2A–D; appendix p 14). In this scenario, the probability of achieving rubella elimination (five infections per million people or fewer) was higher (appendix pp 16–17, 21–22), and the time to elimination was shorter than that for the business as usual scenario (appendix p 14). The magnitude, uncertainty, and time to elimination differed between models. The JHU model predicted a high probability of elimination in all countries over time (figure 2A, C), whereas the PHE model results had more variation in cases across stochastic runs and a lower probability of elimination over time (figure 2B, D). The lower probability of elimination over time in the PHE model was strongly influenced by assumptions about the importation rate (appendix p 23).

The JHU and PHE intensified investment models showed that once the necessary criteria for rubella elimination were achieved, elimination was generally maintained (figure 2A, B). However, sporadic outbreaks were observed in some smaller countries before elimination was achieved, and a small number of countries might be at risk of an outbreak after elimination has been achieved, although the probability of elimination was high (figure 2E). These results were driven by highly transient dynamics, but highlight the need for continued vigilance to survey rubella and congenital rubella syndrome cases and rapidly respond to sporadic outbreaks after elimination is achieved.

**Figure 3:**
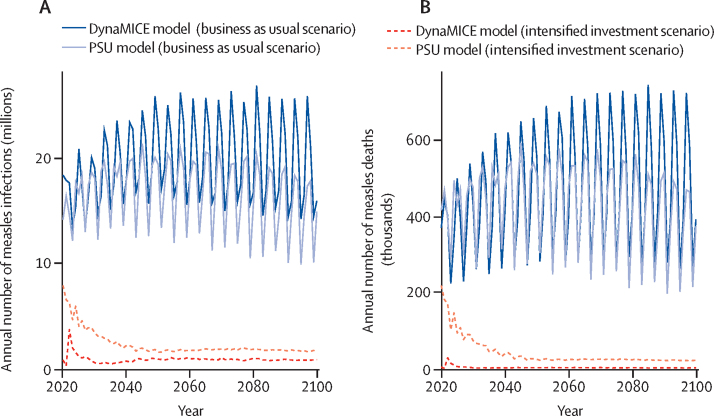
Measles burden Time series of the annual number of measles infections (A) and deaths (B) across 93 countries based on the DynaMICE and PSU models under business as usual and intensified investment vaccination scenarios; the line for each model and scenario (ie, colour and line type) represents the median across 200 stochastic runs. DynaMICE=Dynamic Measles Immunisation Calculation Engine. PSU=Pennsylvania State University.

Under the business as usual vaccination scenario, 16 (17%) of 93 modelled countries in the DynaMICE model and 19 (20%) of 93 modelled countries in the PSU model were projected to have achieved the conditions for measles elimination by 2020 (appendix p 24); of these countries, ten (63%) of 16 countries in the DynaMICE model and 18 (95%) of 19 countries in the PSU model reported less than five measles cases per million people to WHO between 2017 and 2020 (appendix p 25). For the remaining countries, measles cases and deaths were projected between 2020 and 2100 (appendix pp 26–27), resulting in a median of 20 million measles infections (80% prediction interval [PI] 13–37) and 469 000 measles deaths (236 000–862 000) annually in the DynaMICE model and 17 million measles infections (11–22) and 441 000 measles deaths (243 000–620 000) annually in the PSU model (figure 3**;** appendix p 28).

**Figure 4:**
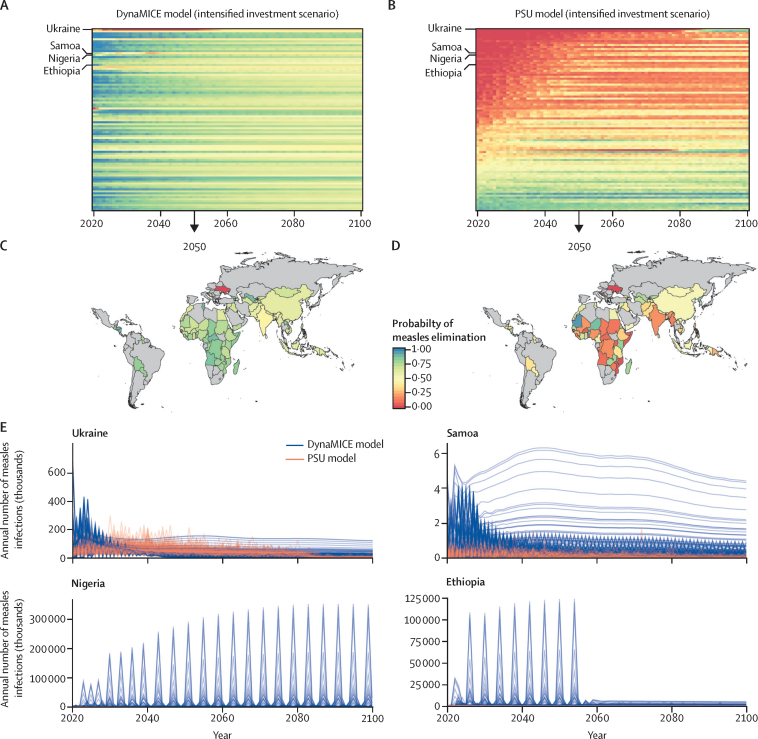
Models of measles elimination Time series of probability of measles elimination by country (rows) between 2020 and 2100 for the DynaMICE (A) and PSU (B) models. The probability of measles elimination by 2050 by country for the DynaMICE (C) and PSU (D) models. The probability of achieving the elimination threshold of no more than five measles infections per million people is shown as a proportion of 200 stochastic runs that would reach the threshold in the intensified investment vaccination scenario. (E) Time series of incident measles infections in Ukraine, Samoa, Nigeria, and Ethiopia across 200 stochastic runs (each line represents a different stochastic simulation) for DynaMice and PSU models. DynaMICE=Dynamic Measles Immunisation Calculation Engine. PSU=Pennsylvania State University.

The intensified investment scenario was predicted to result in marked reductions in the burden of measles cases and mortality. Between 2020 and 2100, in the DynaMICE model, a median of 900 000 measles infections (80% PI 0–33 million) and 3000 measles deaths (0–466 000) were projected to occur annually, and in the PSU model, a median of 2·1 million measles infections (1·3–4·1) and 28 000 measles deaths (18 000–89 000) were projected to occur annually (figure 3; appendix p 28). Additionally, more countries were expected to achieve elimination (appendix pp 29–32) and the time to elimination was shorter (appendix p 24) in the intensified investment scenario than in the business as usual scenario. Model results for the intensified investment scenario show that it is possible for all countries to achieve the necessary criteria for elimination (appendix p 24); however, the probability of elimination is low (figure 4A, B). The probability of achieving measles elimination by 2050 was higher than 75% in only 36 (39%) of 93 modelled countries in the DynaMICE model and 14 (16%) of 93 modelled countries in the PSU model (figure 4C, D).

The probability of reaching elimination was constantly fluctuating for many countries (figure 3A, B), and the magnitude in the probability of measles elimination threshold differed between the DynaMICE and PSU models, resulting from different model structures and assumptions (table 2). Therefore, some countries could not sustain elimination conditions even in the intensified investment scenario. The difficulty in sustaining elimination conditions is partly due to the coverage assumptions of the intensified investment scenario. In many of these countries, routine coverage did not reach high levels (>95%), yet levels of coverage for two doses of measles-containing vaccine (MCV2) were high enough for SIAs to cease, eventually leading to a resurgence of measles infections once enough susceptible individuals had accumulated (figure 4E).

The subnational model of Nigeria provided qualitatively similar results to the other national measles models (appendix p 33). Compared with the business as usual scenario, in the intensified investment scenario, measles burden was reduced (appendix p 34), and the probability of elimination was increased (appendix p 34). The subnational model also explored the impact of two assumptions made in the other measles models: vaccine doses are administered in a spatially uniform distribution across the population and receipt of vaccine doses (eg, first dose of measles-containing vaccine [MCV1], MCV2, and SIA) is uncorrelated.

**Figure 5:**
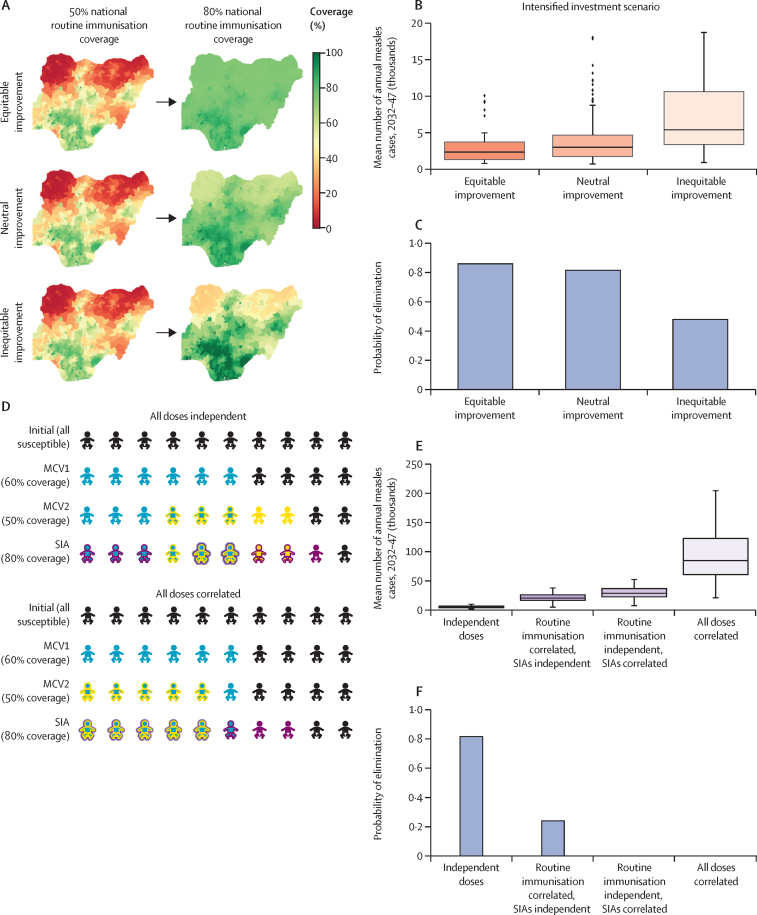
Impacts of different spatial equity scenarios in vaccination and correlation between vaccine dosing opportunities on measles burden and probability of elimination using the Institute for Disease Modeling model for Nigeria Spatial distribution of improvements in routine immunisation coverage under different equity scenarios (A), the impact of spatial equity assumptions on mean annual burden of measles between 2032 and 2047 (B), and probability of measles elimination (C) in the intensified investments scenario. (D) Impact of correlation in access to MCV1, MCV2, and SIA dosing opportunities; for illustrative purposes, we assumed 60% coverage for MCV1, 50% coverage for MCV2, and 80% coverage for a single dose SIA. Impact of dose correlation on mean annual measles burden (E) and probability of elimination (F). In figure parts B and E, boxes show the IQR, whiskers represent 1·5 times the IQR, the horizontal lines show the median, and dots show outliers. MCV1=first dose of measles-containing vaccine. MCV2=two doses of measles-containing vaccine. SIAs=supplemental immunisation activities.

First, increasing spatial equity in routine vaccination requires that improvements in national-level coverage are focused in the districts of Nigeria with lowest coverage first (figure 5A). Higher spatial equity provided a greater reduction in the average annual burden (figure 5B) and increased the probability of elimination (figure 5C) at equivalent levels of national coverage. Second, the assumption of independent dosing with each vaccination opportunity (MCV1, MCV2, SIA) is optimistic. Alternative scenarios (figure 5D), in which MCV2 is correlated with MCV1 (ie, MCV2 is only administered to recipients of MCV1), and SIA doses are correlated with routine doses (ie, SIA doses are first administered to recipients of routine immunisation and only the remaining doses are administered to unvaccinated children), resulted in a considerably higher mean annual measles burden (figure 5E) and lower probability of elimination (figure 5F) than independent dosing scenarios would. In some cases, the measles burden was an order of magnitude higher in the correlated doses scenario than in the independent dosing scenarios, driven by the presence of a population of children who had been repeatedly missed by vaccination programmes (figure 5D). Furthermore, the probability of elimination decreases with correlated dosing and decreased to zero if campaign doses were first administered to the most accessible children who were already reached by routine immunisation (figure 5E).

## Discussion

Global control of measles and rubella is at a crucial point. Substantial progress has been made since 2000 regarding improved MCV1 coverage, and introduction of MCV2 and rubella-containing vaccines. Since 2017, however, these improvements have stagnated and are at risk of being reversed by disruptions caused by the COVID-19 pandemic.^1^ Global and national policy makers need a clear understanding of the gains that can be expected from the continued or increased investment in measles and rubella vaccination. We found that improved coverage in the intensified investment scenario is likely to result in the necessary conditions for rubella elimination in all countries, and in large reductions in measles incidence and mortality despite increases in population size. However, measles elimination will remain unlikely in a subset of countries because, even if the necessary conditions for elimination are achieved, elimination often cannot be maintained in the absence of continued vaccination campaigns.

What would be needed to achieve the established regional goals of measles elimination in all countries? In countries where it is improbable to achieve and maintain measles elimination (ie, annual probability of elimination decreases to less than 0·5 at any timepoint between 2090 and 2100), the median measles incidence will decrease to less than 500 infections per million people by 2100. This is likely to launch the final phase of a measles elimination initiative that implements or accelerates additional strategies (not considered in the models) to capture remaining susceptible individuals. Such strategies could include enhanced surveillance to identify remaining transmission chains, rapid and efficient outbreak response, including ring vaccination of bordering areas, house-to-house vaccination campaigns, school entry checks and catch-up vaccination, transit point vaccinations, and focused efforts to prevent the spread of vaccine misinformation and refusal.^6^, ^21^, ^22^

Targeted vaccination strategies to reach unvaccinated children are likely to be necessary to achieve elimination of measles in all countries. Even when optimistically assuming non-correlated vaccine doses in the national models, a subset of countries remained unable to achieve the necessary conditions for elimination. The subnational analysis for Nigeria shows that targeting SIAs to reach unvaccinated individuals can greatly improve the probability of elimination. Novel strategies to reduce dose correlation and directly target unvaccinated individuals will remain necessary, although not sufficient, to achieve elimination. Novel approaches might include subnational microplanning to create tailored strategies, and to increase delivery infrastructure investment in underperforming areas.^23^, ^24^ Our results also suggest intermittent SIAs might need to continue even after 95% routine coverage is achieved, although targeted strategies to reach unvaccinated individuals and enhanced outbreak response measures were not considered.

Subnational modelling also indicated a higher probability of measles elimination when improvements in national-level routine coverage were pursued in a spatially equitable fashion (ie, improving coverage in the areas with lowest coverage first). Innovative vaccination approaches and technologies can improve vaccination equity within a country. For example, subnational modelling can evaluate strategies to optimise the likelihood of elimination across differential scale-up of routine coverage across administrative units*.* Countries can prioritise strategic planning to ensure targeting of hard-to-reach populations and allow ongoing vaccination opportunities for older children or adults who remain unvaccinated or for vulnerable populations (eg, refugees).^25^, ^26^ Additionally, technologies being developed at present, including microarray patches that deliver measles and rubella vaccines, are easier to administer, more stable, do not require a cold chain, and might increase access among hard-to-reach populations.^27^ Although this modelling focused on spatial equity, the same principles apply to other characteristics associated with heterogeneous measles coverage and clustering of social contacts (eg, gender, ethnicity, religion, and socioeconomic status).^28^ The modelling also did not consider differences in transmission not related to coverage; in practice, subnational regions with low vaccination coverage might have a higher (eg, urban slums) or lower (eg, villages in remote areas) propensity for large outbreaks even if coverage was the same.

In addition to within-country vaccination equity, our results highlight the importance of country vaccination equity across countries. We found that, despite a consistent rate of increase in routine vaccination coverage over time in all countries in the intensified investment scenario, inequitable vaccination coverage rates remained. The intensified investment scenario was based on projections of measles and rubella investments that were likely to be implemented considering historical patterns. The result was a time-varying probability of elimination across regions and the world. Treating equitable coverage as a global goal might motivate the allocation of resources to countries with lower vaccine coverage first. This approach could align the timing by which countries achieve elimination, thus reducing the risk of re-introductions in countries that are trying to sustain their elimination, which is an issue at present in the WHO Region of the Americas. However, considering differing country-specific demography, spatial spread, and contact structure, equitable coverage might not translate to equitable risk. Simulations can be used to differentiate the importance of equitable coverage versus risk at the global level to understand the impact on not only country elimination, but also global eradication.

The ongoing COVID-19 pandemic has major implications for measles and rubella elimination targets. For measles and rubella in particular, the pandemic has adversely affected vaccine uptake in the short term by decreasing access to routine services and delaying vaccination campaigns, thus allowing susceptible individuals to accumulate and increase outbreak risk. In the long term, the pandemic might threaten national economies and the capital of governments for investing in vaccination or surveillance. Therefore, COVID-19 disruptions have the potential to amplify inequities both within and across countries. Conversely, mitigation measures for COVID-19 could also result in a transient unintended positive impact for measles and rubella. Non-pharmaceutical interventions that minimise the number of daily contacts an individual has might reduce not only the risk of SARS-CoV-2 infection but also the risk of measles and rubella infections.^29^ If the reduction in contacts is clinically significant enough to drive the effective reproduction number below 1, a decline in the contact rate could theoretically create an opportunity for measles elimination despite accumulating numbers of susceptible individuals. Sustained elimination would require that infectious individuals are not being introduced from populations where measles continues to circulate and that susceptible individuals are vaccinated as soon as possible, probably through catch-up SIAs.

In addition to model-specific limitations (appendix pp 6–12), the models have several limitations that impact our assessment of measles and rubella elimination. First, the models estimated the true incidence of measles and rubella infection, not reported cases. Subclinical rubella infection and under-reporting of measles and rubella cases means that the defined elimination threshold of five infections per million people might be difficult to translate to an empirical threshold for reported cases. Second, although case importation was accounted for in various ways by the different models (thus allowing for the possibility for re-introduction), no models incorporated explicit global, cross-border transmission dependent on the burden of disease in exporting countries. Consequently, the likelihood of elimination might be overestimated or underestimated depending on the timing of elimination and connectivity with other countries. For example, we might overestimate the likelihood of elimination in a measles-free country that is well connected to high incidence countries by assuming too low an importation rate of infectious individuals. The impact of mobility on the probability of elimination highlights the importance of coordinating national and regional elimination goals and achieving globally equitable reductions in incidence. Third, the national models assume independence of vaccine doses, which is likely to result in the overestimation of the number of vaccinated children. This optimistic assumption, which nevertheless results in some countries being unable to achieve measles elimination, highlights the importance of vaccine equity and new strategies and tools for achieving elimination. Fourth, the national models do not account for subnational heterogeneity in epidemiological and vaccination factors that drive transmission dynamics. To address this gap, lessons learned from the Nigerian subnational model on the sensitivity of homogeneity and equity assumptions are broadly applicable to other countries.

The availability of measles vaccination has resulted in 25·2 million measles deaths being averted since 2000.^1^ Our analyses demonstrate that with sustained vaccination, it is possible to build on these gains and to potentially achieve and maintain measles and rubella elimination this century, although substantial challenges exist. The COVID-19 pandemic has highlighted the potential for infectious diseases to cause widespread disruption to health systems and national economies. Thus, it is crucial that the goal of measles and rubella elimination does not become one more casualty of the COVID-19 pandemic.

## Data sharing

This study does not involve any patient data or participant data. Data collected for the study consisted of demographic data from United Nations World Population Prospects and coverage data from WHO–UNICEF Estimates of National Immunization Coverage, which are freely accessible. Future vaccination scenario coverage projections were prepared by the CDC expert opinion and characterised in the manuscript. Modelled output of time-series of median estimates of measles and rubella elimination probabilities by country and vaccination scenario are available in the appendix (p 55).

## Declaration of interests

MJ, MF, AKW, ST, and JL report grants from the Bill & Melinda Gates Foundation and Gavi, the Vaccine Alliance. EV reports grants from WHO. MJ and MF report grants from WHO. JL and MF report grants from the US Centers for Disease Control and Prevention (CDC) and National Institutes of Health. MF additionally receives grants from the National Science Foundation. SER and MJ are members of the WHO Strategic Group of Experts on Immunization (SAGE) working group on measles and rubella. SER is acting Chair of the Measles and Rubella Initiative and a member of the Measles and Rubella Post Elimination Regional Monitoring and Re-verification Commission of Pan American Health Organization and The Regional Verification Commission for Measles and Rubella Elimination in the Eastern Mediterranean Region. KM is an employee of the Institute of Disease Modeling and the Bill & Melinda Gates Foundation. CB reports grants from WHO, and is currently a contract employee of Diversant, on contract to Merck Research Laboratories. TP, DK, JKK, LKK, HS, SS, KK, PK, and BL declare no competing interests. The findings and conclusions in this Article are those of the authors and do not necessarily represent the views of the US CDC. The views expressed are those of the authors and not necessarily those of the Vaccine Impact Modelling Consortium or its funders.
