# Supplementary material for: Feasibility of measles and rubella vaccination programmes for disease elimination: a modelling study
Source: Lancet Glob Health. 2022 Sep 13;10(10):e1412–22. doi: 10.1016/S2214-109X(22)00335-7 (PMC9557212; doi:10.1016/S2214-109X(22)00335-7)
Supplement: Supplementary appendix [file mmc1.pdf]

THE LANCET
Global Health

Supplementary appendix

This appendix formed part of the original submission and has been peer reviewed. We post it as supplied by the authors.

Supplement to: Winter AK, Lambert B, Daniel Klein D, et al. Feasibility of measles and rubella vaccination programmes for disease elimination: a modelling study. *Lancet Glob Health* 2022; **10:** e1412–22.

Measles and rubella elimination in an inequitable world: Assessing the strengths and limitations of current vaccination strategies using multiple models

Supplementary Material

Amy Winter[1], Brian Lambert, Daniel Klein, Petra Klepac, Timos Papadopoulos, Shaun Truelove, Colleen Burgess, Heather Santos, Jennifer K. Knapp, Susan E. Reef, Lidia K. Kayembe, Stephanie Shendale, Katrina Kretsinger, Justin Lessler, Emilia Vynnycky, Kevin McCarthy, Matthew Ferrari, Mark Jit

CONTENTS

LIST OF FIGURES 2

LIST OF TABLES 2

MATERIALS AND METHODS 3

- **GENERAL APPROACH** 3
  - *Demography* 3
  - *Vaccine coverage scenarios* 4
- **TRANSMISSION MODELS** 6
  - *London School of Hygiene and Tropical Medicine DynaMICE: measles model, multi-national* 6
  - *Pennsylvania State University (PSU): measles model, multi-national* 7
  - *Institute for Disease Modelling (IDM) EMOD: measles model, subnational* 7
  - *Comparison of Measles Models* 9
  - *Public Health of England (PHE): rubella model, multi-national* 10
  - *Johns Hopkins University (JHU): rubella model, multi-national* 11
- **OUTCOME ANALYSIS** 12

RESULTS 13

- **RUBELLA RESULTS** 13
- **MEASLES RESULTS** 24
- **MEASLES RESULTS - SUBNATIONAL MODEL** 33

RESULTS (IN GREYSCALE) 35

- **RUBELLA RESULTS** 35
- **MEASLES RESULTS** 45
- **MEASLES RESULTS - SUBNATIONAL MODEL** 53

COVERAGE ESTIMATES 55

MODEL OUTPUT 55

REFERENCES 56

[1] Corresponding author: awinter@uga.edu

List of Figures

FIGURE S1: MODELED COUNTRIES 3
FIGURE S2: RUBELLA BURDEN WITH MODEL UNCERTAINTY. 13, 35
FIGURE S3: TIME TO RUBELLA ELIMINATION. 14, 36
FIGURE S4: PROBABILITY OF RUBELLA ELIMINATION, JHU, BUSINESS-AS-USUAL. 16, 37
FIGURE S5: PROBABILITY OF RUBELLA ELIMINATION, PHE, BUSINESS-AS-USUAL. 17, 38
FIGURE S6: ANNUAL RUBELLA INFECTIONS. 18, 39
FIGURE S7: ANNUAL CRS CASES. 19, 40
FIGURE S8: ANNUAL CRS DEATHS. 20, 41
FIGURE S9: PROBABILITY OF RUBELLA ELIMINATION, JHU, INTENSIFIED-INVESTMENT. 21, 42
FIGURE S10: PROBABILITY OF RUBELLA ELIMINATION, PHE, INTENSIFIED-INVESTMENT. 22, 43
FIGURE S11: SENSITIVITY ANALYSIS, PHE. 23, 44
FIGURE S12: TIME TO MEASLES ELIMINATION. 24, 45
FIGURE S13: ANNUAL MEASLES INFECTIONS. 26, 46
FIGURE S14: ANNUAL MEASLES DEATHS. 27, 47
FIGURE S15: MEASLES BURDEN WITH MODEL UNCERTAINTY. 28, 48
FIGURE S16: PROBABILITY OF MEASLES ELIMINATION, DYNAMICE, BUSINESS-AS-USUAL. 29, 49
FIGURE S17: PROBABILITY OF MEASLES ELIMINATION, PSU, BUSINESS-AS-USUAL. 30, 50
FIGURE S18: PROBABILITY OF MEASLES ELIMINATION, DYNAMICE, INTENSIFIED-INVESTMENT. 31, 51
FIGURE S19: PROBABILITY OF MEASLES ELIMINATION, PSU, INTENSIFIED-INVESTMENT. 32, 52
FIGURE S20: TIME SERIES OF MEASLES INFECTIONS IN NIGERIA. 33, 53
FIGURE S21: ANNUAL MEASLES INFECTIONS IN NIGERIA, IDM. 34, 54
FIGURE S22: PROBABILITY OF MEASLES ELIMINATION IN NIGERIA, IDM. 34, 54

List of Tables

TABLE S1: DISTRIBUTION OF COUNTRIES BY OBSERVED AND JHU RUBELLA SIMULATED ELIMINATION STATUS AS OF 2020. 15
TABLE S2: DISTRIBUTION OF COUNTRIES BY OBSERVED AND PHE RUBELLA SIMULATED ELIMINATION STATUS AS OF 2020. 15
TABLE S3: DISTRIBUTION OF COUNTRIES BY OBSERVED AND DYNAMICE MEASLES SIMULATED ELIMINATION STATUS AS OF 2020. 25
TABLE S4: DISTRIBUTION OF COUNTRIES BY OBSERVED AND PSU MEASLES SIMULATED ELIMINATION STATUS AS OF 2020. 25

Materials and Methods

General Approach

Four different national and one sub-national model, each evaluating two vaccination scenarios, were employed to assess measles and rubella elimination potential and timing. The four national models have previously been used to generate future projections of the impact of Gavi investments in measles and rubella vaccination in 93 low, lower-middle, and upper-middle income countries, as part of the Vaccine Impact Modelling Consortium (VIMC; https://www.vaccineimpact.org/). These 93 countries (Figure S1) represent areas where most of the measles burden lies and provide a global picture of the feasibility of elimination in the most resource-challenged settings. The subnational-level model was conducted by the Institute for Disease Modelling (IDM) to model measles vaccination subnationally within a single country (Nigeria), providing spatial granularity that complements the national models.

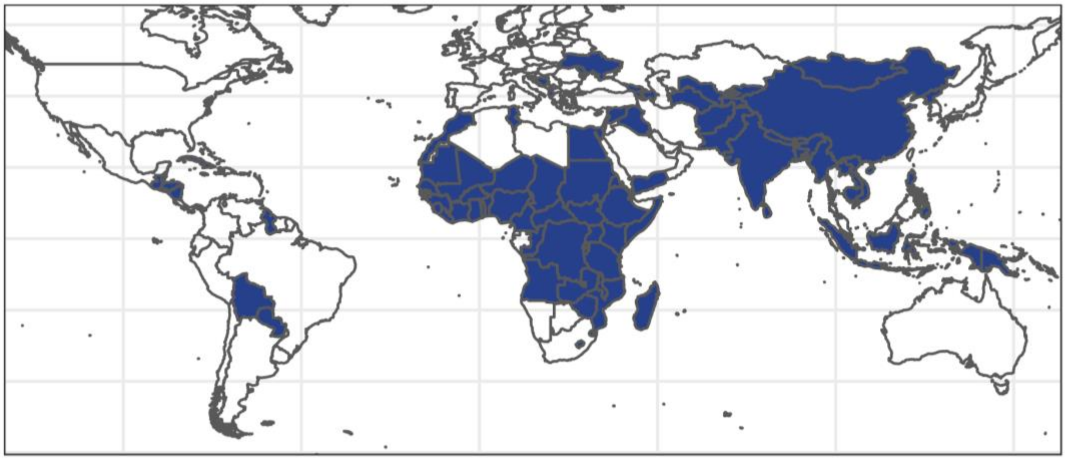

Figure S1: Modeled Countries

The five models used as input scenarios based on historical measles and rubella vaccination coverage for both routine immunization and supplemental immunization activities (SIAs), and future projections based on different methods for estimating long-term trends. The models were used to generate outputs for the years 1980-2100 in terms of the actual (i.e., both reported and unreported) numbers of cases (including congenital rubella syndrome [CRS] cases, for rubella models) and deaths annually by age. The number of cases is then used to examine whether and over what period the annual number of cases falls below 5 per million, representing the point at which incidence is likely low enough to produce transmission interruption at national levels.

Demography

Demographic inputs were standardized across all models. Demographic data, including population by age, live births per year, and background mortality rates, were derived from the United Nations World Populations Prospects, using standardized calculations from the Vaccine Impact Modelling Consortium.[1]

Vaccine coverage scenarios

Vaccine coverage inputs were standardized across models of the same pathogen.

Scenario overview

Two vaccination scenarios were generated to represent different assumptions about future vaccine coverage (2018-2100); these are detailed in Table 1 of the main text:

1) *Business as usual*: Routine coverage remains at 2017 levels, no new measles-containing vaccine second dose (MCV2) or rubella-containing vaccine (RCV) introductions after 2018, and supplemental immunization activities (SIAs) occur at a constant country-specific frequency derived from historical data without cessation. SIAs occurring during 2018-2100 were based on a documented history of national measles SIAs during 2000-2017 in the country of interest and on regional subject matter expert (SME) opinion as to whether a country would continue to conduct such campaigns. For countries that strongly and clearly stated they do not plan to continue with large campaigns to prevent outbreaks (e.g., China, India, and most European countries), SIAs were not continued.

2) *Intensified investments*: Routine coverage increases at a compound growth rate (CGR) of 4.4% up to 99% coverage for countries that have not yet reached elimination or have not reached 95% coverage before 2016; MCV2 and RCV occur during 2018-2024; and SIAs are more frequent, occurring when the unvaccinated population is 75% of size of annual birth cohort or every 4th year, whichever is shorter, until vaccination coverage criteria are met, at which point they cease.

MCV1 coverage estimates

A global median CGR was calculated from the 2000-2017 WHO and UNICEF Estimates of National Immunization Coverage (WUENIC) measles-containing vaccine first dose (MCV1) coverage estimates from countries that had not eliminated measles or rubella nor had reached 95% estimated MCV1 coverage by end-2016.[2] This CGR was used for the intensified investments scenario to estimate MCV1 coverage for these countries prospectively to a limit of 99%. CGR was also applied to countries that have eliminated measles and rubella but have not reached at least 90% MCV1 coverage.

The CGR methodology was developed by the Measles-Rubella Elimination Investment Case work group, U.S. Centers for Disease Prevention and Control (CDC), to measure vaccine coverage growth over time while assuming that gains are retained from year to year. The methodology used reflects a proxy for an asymptotic function, acknowledging the increasing difficulty of reaching every child for immunization as coverage increases. The calculation estimates a growth rate across a historical period, based on starting and ending WUENIC coverage values, and does not attempt to replicate the intervening years.

To calculate the CGR, we assume X_t = coverage at time t ($0 \leq t \leq T$) and i = compound growth rate. At time t, coverage is calculated as

$$X_t = X_0 (1+i)^t$$

For the *intensified-investment* scenario, these calculations were modified to reflect the rate of change of the unvaccinated population over time (instead of the rate of change in coverage) to emphasize the diminishing returns of efforts to vaccinate the hard-to-reach population over time. For example, instead of going from 80% to 90% MCV coverage, we looked at the rate of going from 20% unvaccinated to 10% unvaccinated.

MCV WUENIC data were used for both the starting (2000) and ending (2016) coverage values to estimate a compound growth rate for each country; these results were aggregated to determine a historical growth rate. Three sets of calculations were considered. The first included only countries with improving coverage (non-negative CGRs); the second all countries regardless of whether the CGR was positive, negative or zero. The selected third median CGR value was calculated from all CGR values of countries which had less than 95% coverage in 2000 (i.e. they still had room for growth); this value was 4.44%. The median was chosen instead of the mean because the distribution of CGRs across countries indicated skewness of data. This CGR was then applied to the 2017 baseline WUENIC value and calculated forward over time until 2055; coverage estimates for 2055 were carried forward for 2056-2100. Note that an annual 4.44% rate of decrease in the unvaccinated population is NOT an annual 4.44 percentage point decrease. In other words, if a country had 20% unvaccinated, the next year the unvaccinated population is estimated at 19.1% and not 15.56%.

MCV2 coverage estimates

The global CGR was similarly used to forecast MCV2 coverage prospectively for 2018-2055 for those countries in the not-eliminated, not-95% coverage category. The calculated MCV2 coverage for 2055 was carried forward at a constant level for 2056-2100. For countries that had introduced MCV2 by end-2017, the 2017 MCV2 coverage estimate was used as the starting point for forecasted coverage. For countries that had not introduced MCV2 before 2018, coverage for the year of introduction was estimated as a percentage of its MCV1 coverage that year, based on region- and World Bank income level-specific MCV1-MCV2 differences for countries with ≥4 years of MCV2 data. Forecasted MCV2 coverage was subject to dynamic capping to ensure that MCV2 did not exceed MCV1 and that relative values were realistic.

SIAs

For the *business-as-usual* scenario, SIAs occurring during 2018-2100 were based on a documented history of national measles SIAs during 2000-2017 in the country of interest and on regional subject matter expert (SME) opinion as to whether a country would continue to conduct such campaigns. For countries that strongly and clearly stated they do not plan to continue with large campaigns, SIAs were discontinued; these included China, India, and most European countries (*personal communication from NIP*).

All countries eligible for SIAs from the *business-as-usual* scenario were also eligible for SIAs in the intensified investments scenario. SIAs were set to occur when the unvaccinated population reached 75% of the size of an annual birth cohort, or every 4th year, whichever interval is shorter. Cessation of SIAs

was set to occur when MCV1 reached 95% and MCV2 reached 93% coverage, unless otherwise defined. For *business-as-usual*, since no changes in coverage occurred from 2018-2100, cessation was defined solely on SME opinion or other information. For *intensified-investment*, some countries only had 1-2 SIAs before they met all cessation criteria. In some cases, future SIAs were included for countries where no previous campaign had occurred during 2000-2017, but for which an SIA occurred during 2017-2019 or was "planned" per the UNICEF vaccination calendar from 2019.

Transmission Models

The above scenarios were evaluated within the context of two national models [3,4] and one subnational model [5] for measles transmission, and two national models for rubella transmission.[6–8] All the models incorporated transmission, and hence captured both the direct and indirect (herd) impact of vaccination. Each model captures uncertainty in its outcomes through some combination of randomly sampling input parameter values over uncertainty distributions, and in some cases, incorporating first order uncertainty (i.e., randomness in the actual processes in the model). Each model was run for 200 stochastic simulations over the period from 1980 to 2100 (2050 for the sub-national measles model). Models are summarized in Table 2 in the main text; additional details are provided below.

London School of Hygiene and Tropical Medicine DynaMICE: measles model, multi-national

DynaMICE (Dynamic Measles Immunization Calculation Engine) is a realistic age-stratified compartmental model of measles infection transmission in vaccinated and unvaccinated individuals.[3] The model is coded in R and Fortran. Each country being modelled has a population that ages in weekly age classes up to the age of 3 years, and in yearly age classes between the ages of 3-100 years.[9] The population in the model can be susceptible to, infected with or recovered from measles, with transmission dependent upon the current proportion of infected individuals in the population, as well as the effective contact rate between different age groups.

Modelled populations can have immunity to measles due to either natural immunity following measles infection (assumed to be lifelong), vaccine-induced immunity, or short-lived passive immunity through maternal antibodies. Vaccines are assumed to be "all or nothing", so that individuals receiving the vaccine are either fully protected or not at all.[10] We assume that vaccination gives lifetime protection if it successfully elicits an immune response, and that vaccinating already-infected individuals does not increase the rate of infection clearance (i.e., the vaccine has no therapeutic action).

Age-dependent contact patterns in the population are based on diary-based contact studies; in this analysis, the default contact matrix for Great Britain in the POLYMOD study is used as this provides the best fit to measles epidemiology in most countries.[11] This corresponds to a probability of transmission per contact of about 3. The model parameters are not directly calibrated to data; instead R_0, the number of people infected by a single infected person in a completely susceptible population, is varied widely between 10-20.[12] The average infectious period of measles was assumed to be 14 days.[12] Previous comparisons of model estimates of measles incidence show similar trends over time as estimates over the same period from Global Burden of Diseases 2019 by the Institute for Health Metrics and Evaluation

(IHME).[13] DynaMICE produces lower estimates of incidence than figures from IHME, but higher estimates than those from WHO's case notifications.

The DynaMICE model captures uncertainty in epidemiological dynamics by probabilistic sampling from a distribution representing the possible values of the basic reproduction number (R0), and uncertainty in disease burden by probabilistic sampling from a distribution representing the possible values of the case-fatality risk (CFR).

Pennsylvania State University (PSU): measles model, multi-national

The PSU measles model is a semi-mechanistic, age-structured, discrete time-step, annual SIR model which simulates the aggregate number of cases over one-year time steps.[4] While this is coarse relative to the time scale of measles transmission, it matches the annual reporting of measles cases available for all countries since approximately 1980, through the WHO Joint Reporting Form (JRF). To account for the many fine-scale dynamics that are being summed over a full year, we model the number of infections by country, year, and age class as an increasing function of the fraction of the population susceptible in that age class at the start of the year. The country- and year-specific annualized attack rate is parameterized to each country independently using a state-space model fitted to observed annual cases reported through the JRF from 1980-2016 as previously described in [14–16]. The model's major source of uncertainty is in the variance in the annualized attack rate function. This value reflects the degree to which the observed dynamics conform to the deterministic form of the attack rate function.

The number of susceptible individuals in each single-year age class is equal to the number not infected in the previous year, nor immunized through supplemental immunization activities (SIAs). The number susceptible is further deprecated by the crude death rate. The efficacy of doses administered through SIAs is assumed to be 99%. The number of susceptible individuals in the 0-1 year old age class is assumed to be 50% of the annual live birth cohort; this assumes that all children have protective maternal immunity until 6 months of age. First and second dose routine measles vaccination is assumed to be administered before the start of the time step; thus the number susceptible is further reduced by the product of the coverage and the efficacy. Efficacy is assumed to be 85% and 93% for the first dose in countries delivering at 9m and 12m of age, respectively, and assumed to 99% for the second dose.

Deaths are calculated by applying an age- and country-specific case fatality ratio (CFR) to each country. CFRs for cases below 59 months of age for all countries were taken from [17]; CFR for cases above 59 months of age are assumed to be reduced by 50%. Forward simulations of this model assume random variation in the annual attack rate.

Institute for Disease Modelling (IDM) EMOD: measles model, subnational

The EMOD model is an individual-based stochastic model of disease transmission, written in C++ with support in Python, with accommodation for campaign implementation, heterogeneous transmission, and spatially segregated populations coupled by individual-level migration.[5] EMOD has been utilized to model transmission of many diseases; a past version of this model applied specifically to measles in Nigeria can be found in [18]. The measles model builds upon a discrete-time, individual-based form of the

well-known MSEIR (maternally protected-susceptible-exposed-infectious-recovered) model, with age-structured transmission with 3 age classes: 0-1 year, 1-2 years, and 2+ years old. The contact matrix is constrained to scale the force of infection onto individuals in a specific age class independent of the age of infected shedders. Annual forcing in transmission is implemented to reproduce the seasonal outbreak structure in historical case count data. There is no waning of immunity; all individuals who enter the recovered state will remain immune until death.

Nigeria is divided into metapopulations representing the Local Government Areas (LGAs), the second-level administrative districts of Nigeria. Birth and mortality rates vary regionally in Nigeria; however, for simplicity's sake, these are spatially homogeneous in the model. The mortality rate is implemented as a function of age, with values obtained from the Demographics and Health Survey, 2013,[19] with the ratio of mortality rate versus age distribution held constant over time. The birth rate is interpolated year-by-year so that the yearly nationwide population tracks the total population over time defined in the VIMC demographic data for Nigeria.

Individuals of any disease state and age can migrate between the 774 LGA metapopulations (nodes); the infection process in a given node at a given time-step is governed by individuals in that node at that time-step only. Migration is modeled as one-day round-trips to prevent unrealistic population accumulation in the largest nodes. Individuals migrate between nodes with rates to destination nodes defined according to a gravity-like model of migration, computed for all connections; however, each node only retains its 8 nearest spatial neighbors and the next 60 largest migration rates as possible destinations.

Almost all children are born with maternal protection and are assumed to be completely protected through some time, after which they transition into the susceptible class. Each infant draws a duration of maternal protection at birth from a sigmoid distribution fit to the data on children of naturally immune mothers from [20]. Maternally protected or susceptible children can be vaccinated, and if immunization is successful they will transition directly into the recovered class. When an individual receives a vaccine, the probability that they will transition into the R class is dependent on the individual's age through a piecewise linear function, and dependent on whether they are still maternally protected or not.

National-level routine immunization coverages over time are defined based on the scenarios defined above. As a starting point for spatial heterogeneity, average routine immunization (RI) coverage for each LGA is defined as the birth-weighted average RI coverage of all 1kmx1km pixels within each LGA boundary. In the primary scenario simulations, as national-level RI coverage improves, the LGA-level coverages will improve in direct proportion to (1 – coverage). In effect, the shape of the distribution is preserved, and each LGA improves proportionally to match the target national-level coverage. Alternative scenarios explore situations in which improvement in routine coverage is more equitable (lower-coverage districts improve faster) or more inequitable (highest-coverage districts improve faster).

The EMOD model applied to measles in Nigeria has previously been published in[18]. For this manuscript, the spatial resolution was increased to LGA level, and eight free parameters of the model were recalibrated to measles lab-confirmed and epi-linked suspected measles cases from Nigeria, 2009-2018: The force of infection in the three age classes described above; the beginning, end, and amplitude of the high-infectivity season; and two parameters of a gravity model of migration - an overall scaling for total migration and the exponent governing the relationship between migration and distance (the two

population terms in the gravity model are assumed to be linear). The 8 parameters are jointly calibrated through Bayesian History Matching with emulation, targeting the age distribution of measles infections, the relative proportion of measles infections by month, and the distribution of province-level pair-wise correlations of the time-series of measles infections. The resulting calibration is a volume of plausible parameter space, and different stochastic runs of the model draw new parameter sets as well as a new random number seed for the simulation.

Campaign immunization is not assumed to be instantaneous; children covered by a campaign will seroconvert at a time from 1-60 days after the campaign begins. The historical campaign calendar is used to set the start days of campaigns in the past, and campaigns in the future are assumed to begin on October 1 in the northern regions of Nigeria and December 1 in the southern regions, to roughly reflect the historical campaign phasing in Nigeria. In the main simulations, campaign coverages at LGA-level are assumed to be independent and random, uncorrelated with routine immunization and uncorrelated from one campaign to the next.

In alternative simulations, we induce correlations between campaign and routine coverage to highlight the impact of heterogeneity in accessibility and the importance of finding zero-dose children. In these alternative simulations, we take a "worst-case" scenario from the standpoint of campaign effectiveness - i.e., vaccine doses in campaigns are assumed to go first to the children who received both routine doses, next to children who received only one dose, and only if there are doses remaining, do they go to children missed by routine immunization.

The uncertainty in the results from the IDM models reflects both stochasticity in the dynamic processes in the model (transmission, migration, importation, birth/death) and parametric uncertainty in the model parameters governing age-specific force of infection, seasonal profile, and spatial connectivity. 500 simulations were run in the two immunization scenarios, each of which sampled both a new model parameter set and a new random seed for the stochastic dynamics. The 500 parameter sets & random seeds were replicated across the modeled scenarios to simplify comparison of the differences between scenarios. The spatial equity and vaccine correlation sub-analyses presented in Figure 4 of the main text used a reduced set of 50 simulations (again with replication of parameters and random seeds across scenarios).

Comparison of Measles Models

Differences in national measles model outputs are primarily the result of how the models incorporate vaccination coverage. The DynaMICE model uses a more direct translation of coverage to impact than the PSU model. Taking into account age-specific vaccine effectiveness and dose correlations, vaccination coverage proportionately reduces the number of susceptibles in the DynaMICE model, which is then translated into impact using a mass-action SIR-type model with age-dependent mixing. The PSU model fits a logistic relationship between the annual attack rate and the proportion of the susceptible population in each country independently (methods described in Eilertson et al 2019[14]). The estimated slope and intercept determine the speed at which measles cases respond to increases in the proportion of the population susceptible (i.e., coverage). For example, a steep slope indicates that the probability of infection increases quickly with a small increase in the proportion of the population susceptible (i.e. a

large outbreak is likely after a small disruption). A shallow slope means that a large reduction in coverage (i.e., a large increase in susceptibles) would be necessary to generate a large and immediate outbreak. The shape of this function is fit to the annual measles time series from 1980 to 2019. As a result of these differences, the DynaMICE model is more sensitive to vaccination changes close to the herd immunity threshold, is more likely to reduce to zero cases and stay low until the accumulation of susceptible individuals reaches herd immunity, at which time is likely to simulate a faster increase in the number of cases. This has a particular impact in countries with high vaccination coverage but continuing transmission in which the DynaMICE model is more likely to predict elimination than the PSU model. [21] The IDM model uses a conceptually similar mechanistic framework as DynaMICE, but is an individual agent-based model rather than a compartmental model, and in this work was applied to only one country to explore the impact of subnational heterogeneities in dose and disease burden distribution.

Despite these model differences, the trends in cases and deaths are consistent across models and the magnitude of cases and deaths are relatively similar in the two different scenarios.

Public Health of England (PHE): rubella model, multi-national

The PHE model used an age and sex-structured, deterministic, compartmental model of the transmission dynamics of rubella based on previous work [8,22,23] and adapted to allow infections acquired outside each country. The population comprises people aged 0-99 years and is stratified into those with maternal immunity (lasting 6 months), susceptible, pre-infectious (infected but not yet infectious), infectious and immune states, using annual age bands and a "Realistic Age Structure".[9] Country-specific birth and age-specific death rates were fixed at 2010 levels and calculated from UN population survival data for 2010-15 [1] respectively. The supplement to [22] provides the model's differential equations.

The force of infection (rate at which susceptibles are infected) changes over time and is calculated using the number of infectious individuals and the effective contact rate (rate at which infectious and susceptible individuals come into effective contact). Contact is described using a "Who Acquires Infection from Whom" (WAIFW) matrix. In this WAIFW matrix, the effective contact rate differs between <13 and ≥13 years, with its relative size based on contact survey data.[11] So that the transmission model reproduced available observed data on the age-specific proportion seropositive before RCV had been introduced, the effective contact rate was calculated from the average force of infection in persons <13 and ≥13 years olds, which, in turn had been estimated from the corresponding age-stratified rubella seroprevalence data by fitting catalytic models to data.[23] Seroprevalence data were available for 28 countries (see [8,23]). For countries lacking seroprevalence data, we used data from countries in the same WHO region.[8,23] Confidence intervals (CI) on the force of infection were calculated using 1000 bootstrap-derived-seroprevalence datasets.[8,23] We explored the effect of high, medium and low levels of importations, defined as 0.01%, 0.005% and 0.001% of susceptibles infected abroad monthly. Unless otherwise stated, the output was obtained assuming high levels of importations.

Country-specific numbers of CRS cases in year *y* during 2020-2100 were calculated by summing the number of CRS cases born each day to women aged 15-49 years. As assumed elsewhere, infection during the first 16 weeks of pregnancy carries a 65% risk of the newborn having CRS.[8,22–24] The number of CRS deaths in year *y* was calculated by multiplying the number of CRS cases born in year *y* by the assumed case fatality rate (30%). The number of DALYs for cases in year *y* was calculated by multiplying the

number of CRS cases in year *y* by the corresponding DALY (from [8,25]), which was based on the country-specific World Bank Income group for 2017.[26] Both the DALYs and the assigned World Bank income group remained fixed over time.

The model includes uncertainty in five country-specific parameters, as described elsewhere:[8] a) the age-dependent contact parameters (calculated from 1000 bootstrap-derived values of country or region-specific values for the pre-vaccination force of infection, obtained from seroprevalence studies[8,23]), b) the risk of a child being born with CRS if the mother was infected whilst pregnant (assumed to vary between 50 and 75%, based on observed studies[27-29]); c) the CRS-related mortality rate (assumed to vary between 10 and 30%, based on observed studies[30-32]); d) the vaccine efficacy (assumed to vary in the plausible range of 85%-99%) and e) the vaccine coverage (assumed to differ by up to 10% from that projected, which is plausible). In addition, we explored the sensitivity of model predictions to assumptions about the importation of infectious people, as described in this manuscript.

Johns Hopkins University (JHU): rubella model, multi-national

The JHU model is a discrete-time, stochastic, age-structured, compartmental rubella transmission model, building from previous work describing rubella dynamics.[6,7] The key feature of the model is a matrix that at every time-step defines transitions from each combination of epidemiological stage (maternally immune 'M', susceptible 'S', infected 'I', recovered 'R', and vaccinated 'V', taken to indicate those effectively vaccinated) and age group (1 month age groups up to 20 years old, then 1 year age groups up to 100 years old) to every other possible combination of epidemiological stage and age group. The discrete time-step was set to roughly two weeks (i.e., 24 time-steps in a year), the approximate generation time of rubella.

Humans are born either directly into the 'susceptible' class or move there as passively acquired 'maternal immunity' wanes over the first year of life. As individuals age, they can be exposed to either vaccination which, if successful, moves them permanently into the 'vaccinated' class, or to natural infection, moving them to 'infected' for a time-step (or rubella generation) then permanently into the 'recovered' class. In addition to these epidemiological transitions, there are demographic transitions including births, deaths, and aging.

Demographic parameters (population size, crude birth rates, and age-specific death rates) were supplied by the Vaccine Impact Modelling Consortium, and vaccination coverages were time- and country-specific, as defined by the scenarios described above. We further adjusted vaccination coverage based on the assumptions that repeated vaccination activities are not completely independent, and that a portion of the population may always remain inaccessible to vaccination campaigns. We assumed the age- and time-specific proportion inaccessible corresponded to WUENIC diphtheria, tetanus, and pertussis (DTP) routine vaccination rates.[2] Duration of maternal immunity[33] and vaccine efficacy[34] were assumed from published literature and are constant across time and country. The annual introduction of infected individuals scaled with the median population size of each country, ranging from 24 to 0.006, and was set to trigger an outbreak if the size of the susceptible population was large enough to induce transmission, but small enough not to alone surpass elimination thresholds.

Country-specific transmission to individuals in age group *a* from individuals in age group *j* for each time-step *t* is defined by the mean transmission from individuals in age group *j* to age group *a*, and the magnitude of seasonal fluctuations (assumed 0.15[6] and constant over time and country), estimated by rescaling population-adjusted age-contact rates (time constant and country-specific[35]) to reflect the assumed basic reproductive number (R_0) of rubella. R_0 distributions were country-specific and estimated by fitting a dampened exponential model[36] with likelihood-based Markov chain Monte Carlo algorithm to published rubella immunoglobulin G (IgG) seroprevalence data. Model parameters (i.e., R_0) were fit to empirical data, however the transmission model is not directly fit to data. Model uncertainty includes process uncertainty for all epidemiological and demographic transitions and uncertainty on the value of R_0.

Age- and time-specific CRS cases were estimated from each country's model output by multiplying the age-specific number of susceptible individuals, the sex ratio of the population, the age-specific fertility rate, the probability of becoming infected over 16-week period, and finally the probability of CRS following rubella infection during the first 16 weeks of pregnancy (estimated 0.59 [27-28, 37-39]). Fetal and child deaths were estimated from the number of CRS cases as mean estimated 9.3 per 100 live births, and mean estimated 1.4 per 100 live births, respectively.[40]

The model incorporates parameter uncertainty (i.e., country-specific rubella basic reproductive number, gestational age-specific CRS risk, and age-specific CRS death rates) and uncertainty of stochastic processes for every epidemiologic and demographic transition in the model. The model was validated to capture demographic changes and intra-annual rubella transmission.[6,41]

Outcome analysis

As infected individuals are periodically introduced into the metapopulation network, true elimination of measles (sustained periods with no measles infections anywhere in the simulation) does not occur, though short periods of time with zero cases can occur. As such, we use an annual incidence threshold (5 cases per 1M population) as a proxy to indicate fragile transmission that would likely be interrupted in the absence of continued importation. We also explore the duration of continuous time periods spent below these thresholds to differentiate between temporary low-incidence years (e.g., in years following a large outbreak or highly effective SIA) vs. long-term maintenance of incidence below threshold for multiple years, the latter indicating more robust achievement of near-elimination conditions. In the national models this is evaluated with a time series that estimates the proportion of 200 stochastic runs that fall below the 5 per million threshold at any given time point.

Results

Rubella Results

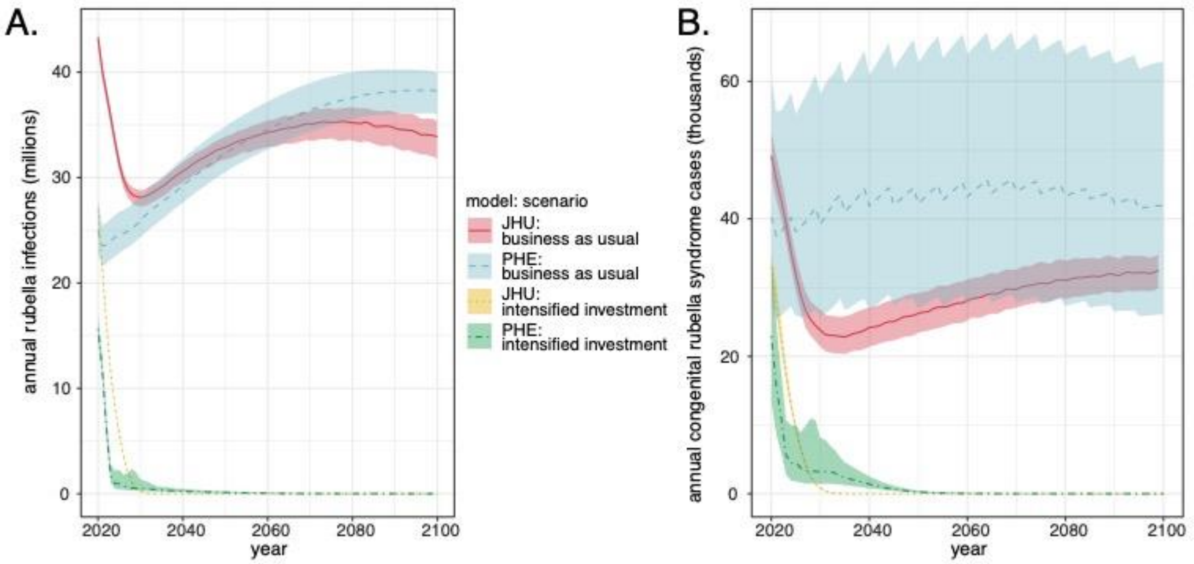

Figure S2: Rubella burden with model uncertainty.

Time series of the annual aggregate number of rubella infections (A) and CRS cases (B) across 93 countries based on Johns Hopkins University (JHU) and Public Health England (PHE) models given business-as-usual and intensified-investment vaccination scenarios. The line (i.e., color and line type) and ribbon (i.e., transparent shading) for each model and scenario represent the median and 80% prediction intervals across 200 stochastic runs.

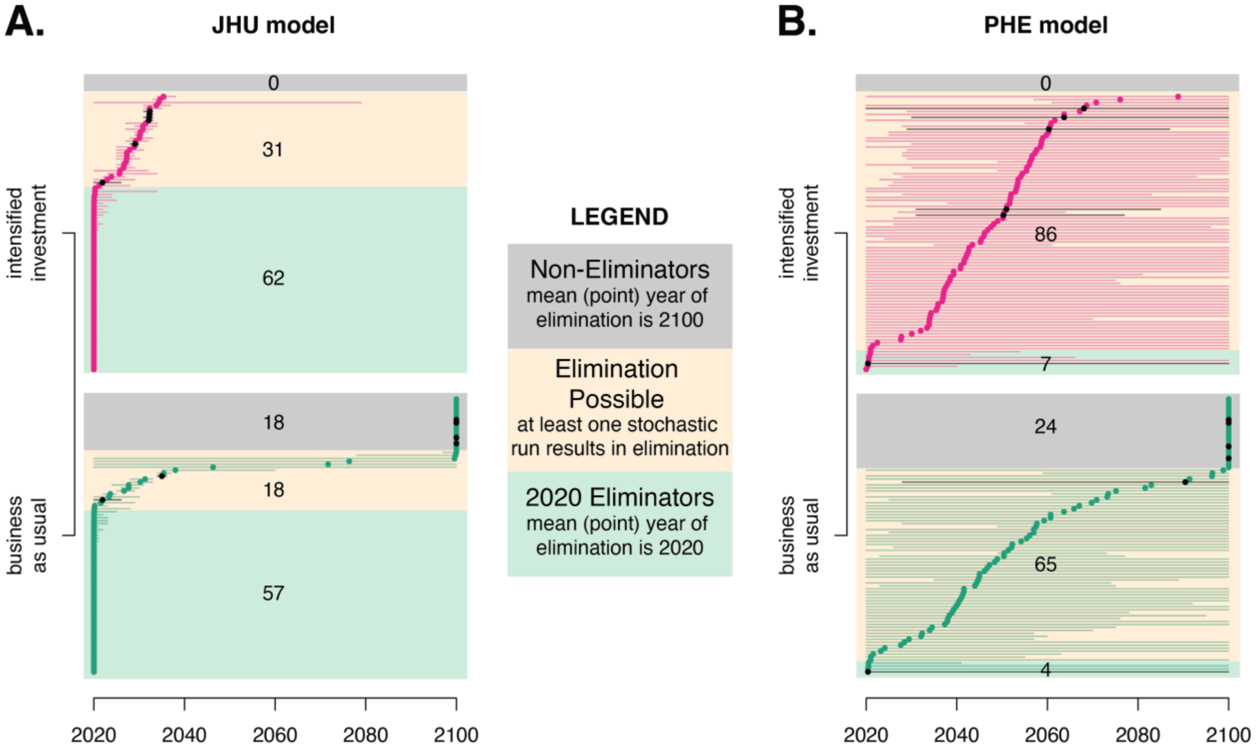

Figure S3: Time to rubella elimination.

The year that countries reach the "elimination" threshold of 5 rubella cases per one million population for JHU (**A**) and PHE (**B**) models for business-as-usual (green) and intensified-investment (pink) vaccination scenarios. The point represents the mean, and the bars show the minimum and maximum values from 200 runs. The black points and lines represent Pakistan, India, Nigeria, Ethiopia, and the Democratic Republic of Congo (DRC). Simulations that never result in cases below the elimination threshold are assigned the elimination year 2100. Countries are ordered by the mean year they reach elimination and order will differ by scenario and model. The transparent green, tan, and grey boxes characterize countries as 2020 eliminators, elimination possible, and non-eliminators, respectively.

	Observed Elimination	Observed Non-Elimination	Total
**JHU Simulated Elimination**	56 (61.5%)<br>*1,113,825 (20.6%)*	0 (0%)<br>*0 (0%)*	56
**JHU Simulated Non-Elimination**	34 (37.4%)<br>*4,248,819 (78.6%)*	1 (1.1%)<br>*43,849 (0.8%)*	35
**Total**	90	1	91 (100%)<br>*5,406,494 (100%)*

Table S1: Distribution of countries by observed and JHU rubella simulated elimination status as of 2020.

JHU simulated 'elimination' are countries that reach the "elimination" threshold of 5 rubella infections per one million population by mean year of 2020 (i.e., 2017-2020 across 200 simulated runs) given **business-as-usual** vaccination scenarios. Observed 'elimination' are countries that reported annual < 5 rubella per one million population between 2017 and 2020 to the WHO via Joint Reporting Form. Note that 2 countries did not report cases (including zero cases) 2017-2020 to the WHO and were therefore were not included in the table. The top row (black text) represents the number of countries (percentage); the bottom row (blue italic text) represents the 2020 population total (percentage).

	Observed Elimination	Observed Non-Elimination	Total
**PHE Simulated Elimination**	4 (4.4%)<br>*1,492,518 (27.6%)*	0 (0%)<br>*0 (0%)*	4
**PHE Simulated Non-Elimination**	86 (94.5%)<br>*3,870,126 (71.6%)*	1 (1.1%)<br>*43,849 (0.8%)*	87
**Total**	90	1	91 (100%)<br>*5,406,494 (100%)*

Table S2: Distribution of countries by observed and PHE rubella simulated elimination status as of 2020.

PHE simulated 'elimination' are countries that reach the "elimination" threshold of 5 rubella infections per one million population by mean year of 2020 (i.e., 2017-2020 across 200 simulated runs) given **business-as-usual** vaccination scenarios. Observed 'elimination' are countries that reported annual < 5 rubella per one million population between 2017 and 2020 to the WHO via Joint Reporting Form. Note that 2 countries did not report cases (including zero cases) 2017-2020 to the WHO and were therefore were not included in the table. The top row (black text) represents the number of countries (percentage); the bottom row (blue italic text) represents the 2020 population total (percentage).

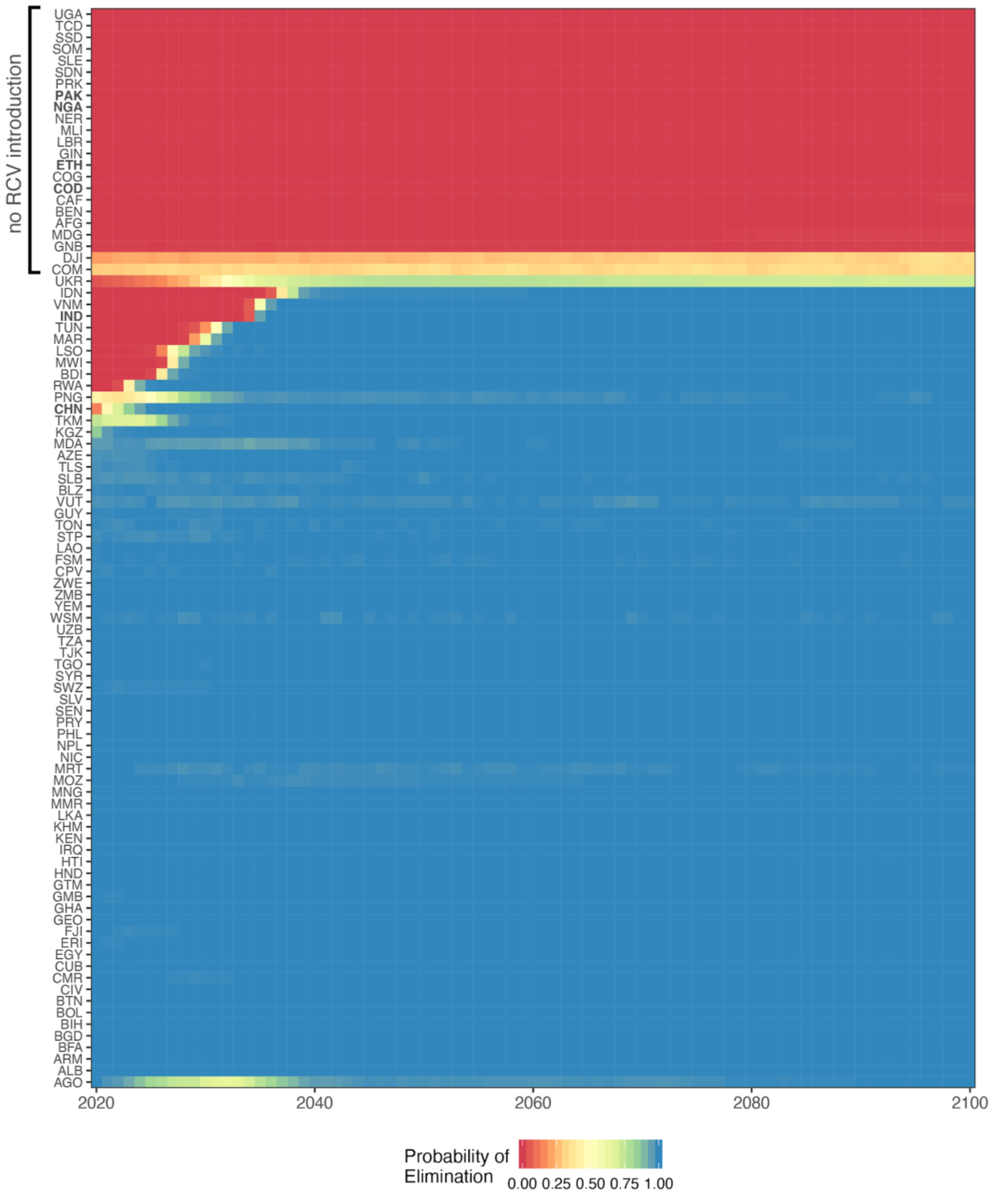

Figure S4: Probability of rubella elimination, JHU, Business-as-usual.

JHU model estimates of the annual probability of reaching the “elimination” threshold of 5 rubella cases per one million population, shown as the proportion of 200 stochastic runs which reach the threshold in the **business-as-usual** vaccination scenario. Countries are listed according to their International Organization for Standardization 3166 alpha-3 country codes (ISO 3; https://www.iso.org/iso-3166-country-codes.html).

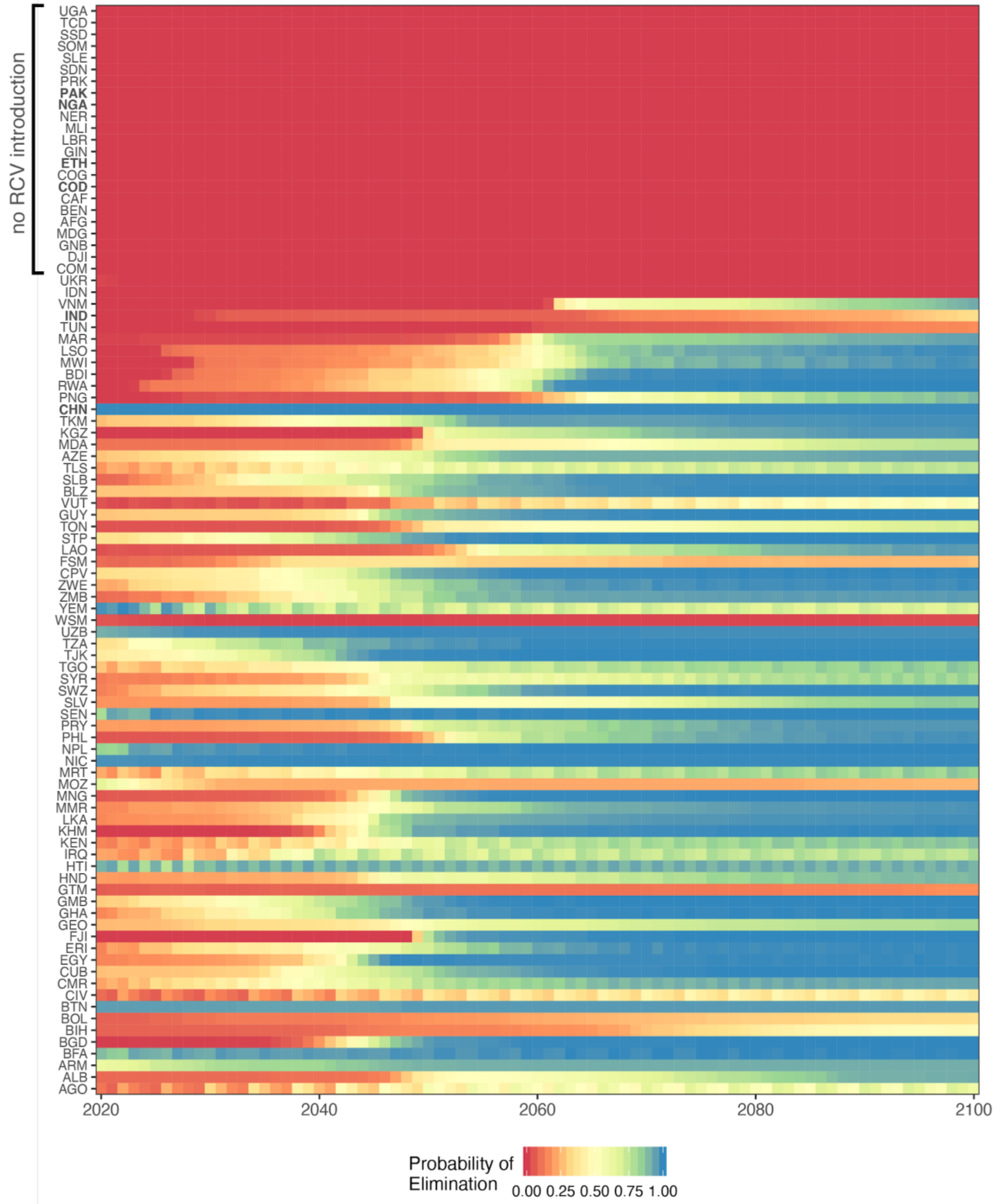

Figure S5: Probability of rubella elimination, PHE, business-as-usual.

PHE model estimates of the annual probability of reaching the "elimination" threshold of 5 rubella cases per one million population, shown as the proportion of 200 stochastic runs which reach the threshold in the **business-as-usual** vaccination scenario. Countries are listed according to their International Organization for Standardization 3166 alpha-3 country codes (ISO 3; https://www.iso.org/iso-3166-country-codes.html).

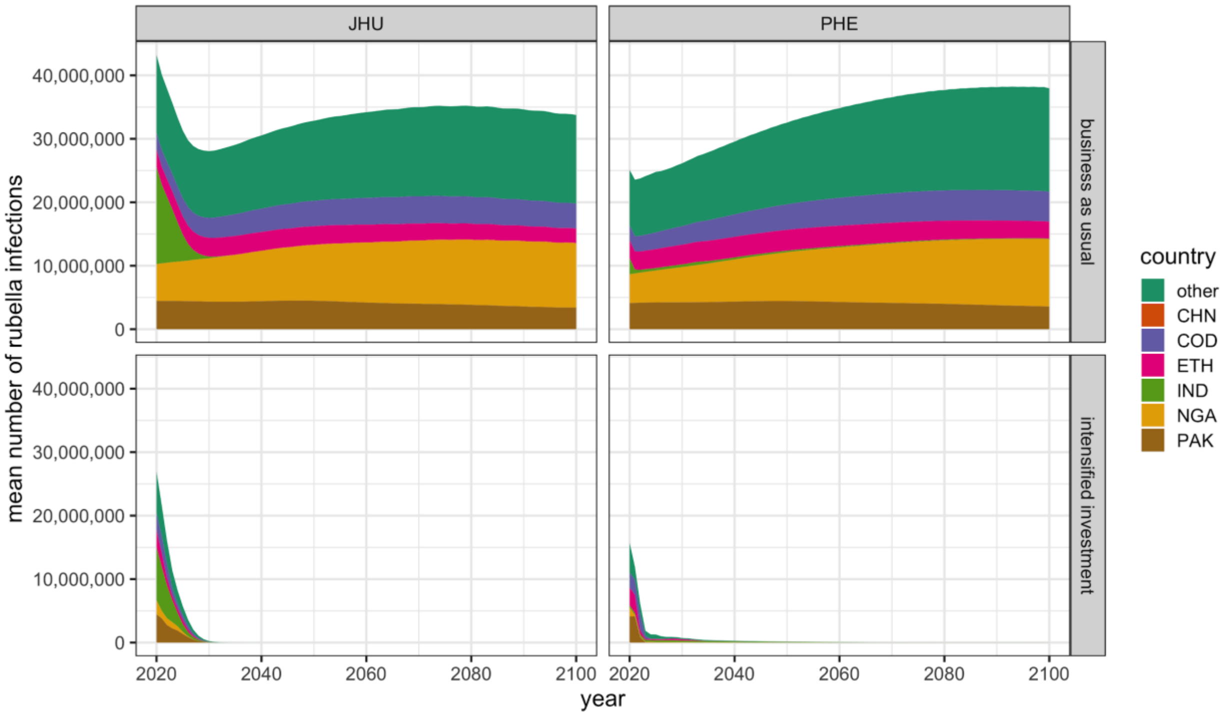

Figure S6: Annual rubella infections.

The mean number of annual rubella infections for China (CHN), Democratic Republic of Congo (COD), Ethiopia (ETH), India (IND), Nigeria (NGA), Pakistan (PAK), and all other countries combined (other) per JHU (left column) and PHE (right column) models for the business-as-usual (top row) and intensified-investment (bottom row) vaccination scenarios.

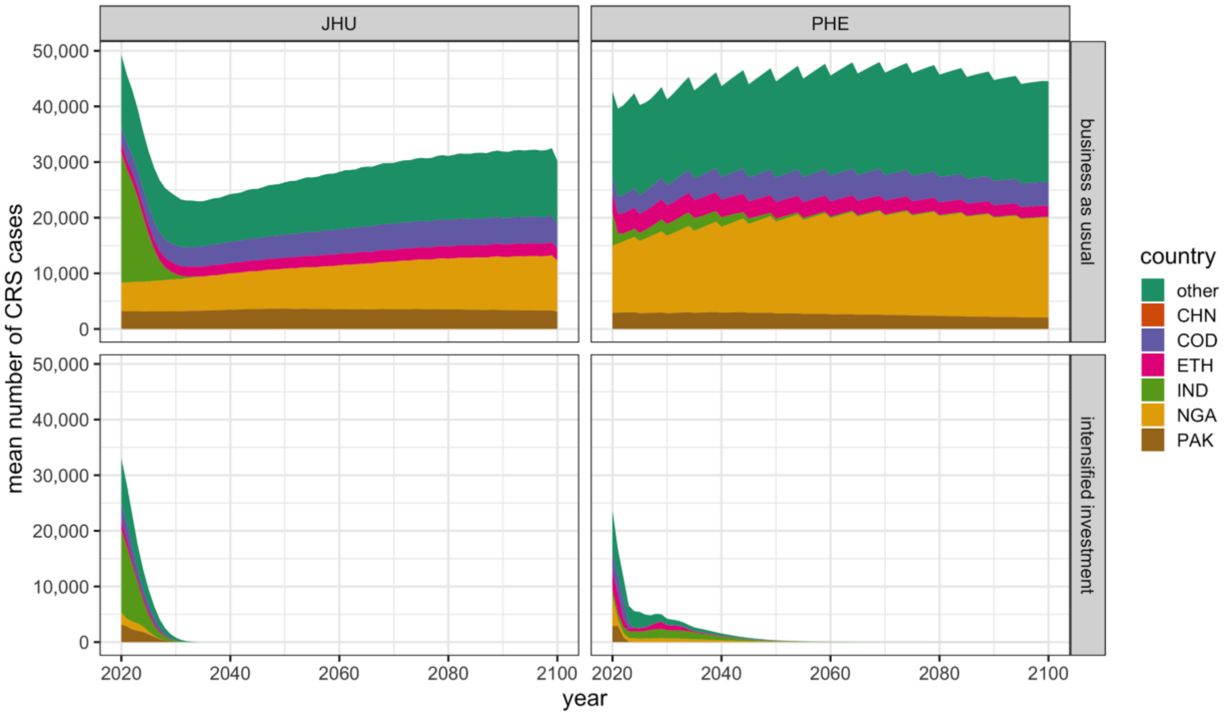

Figure S7: Annual CRS cases.

The mean number of annual CRS cases for China (CHN), Democratic Republic of Congo (COD), Ethiopia (ETH), India (IND), Nigeria (NGA), Pakistan (PAK), and all other countries combined (other) per JHU (left column) and PHE (right column) models for the business-as-usual (top row) and intensified-investment (bottom row) vaccination scenarios.

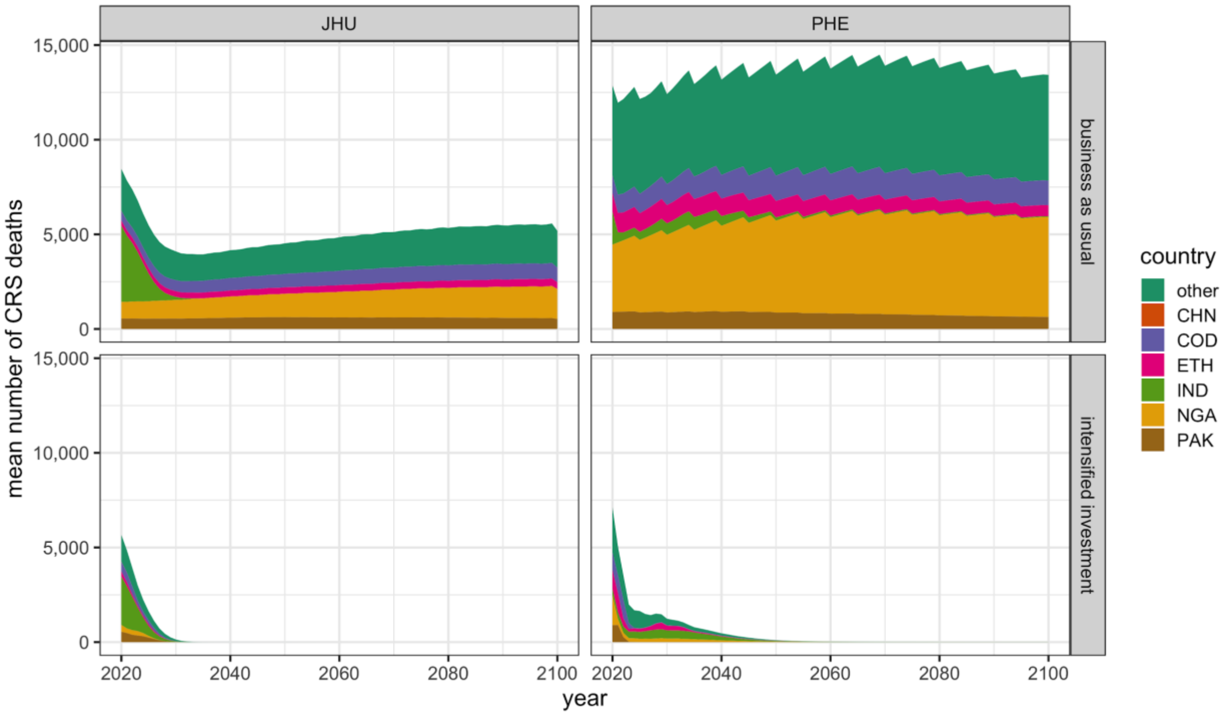

Figure S8: Annual CRS deaths.

The mean number of annual deaths due to CRS for China (CHN), Democratic Republic of Congo (COD), Ethiopia (ETH), India (IND), Nigeria (NGA), Pakistan (PAK), and all other countries combined (other) per JHU (left column) and PHE (right column) models for the business-as-usual (top row) and intensified-investment (bottom row) vaccination scenarios.

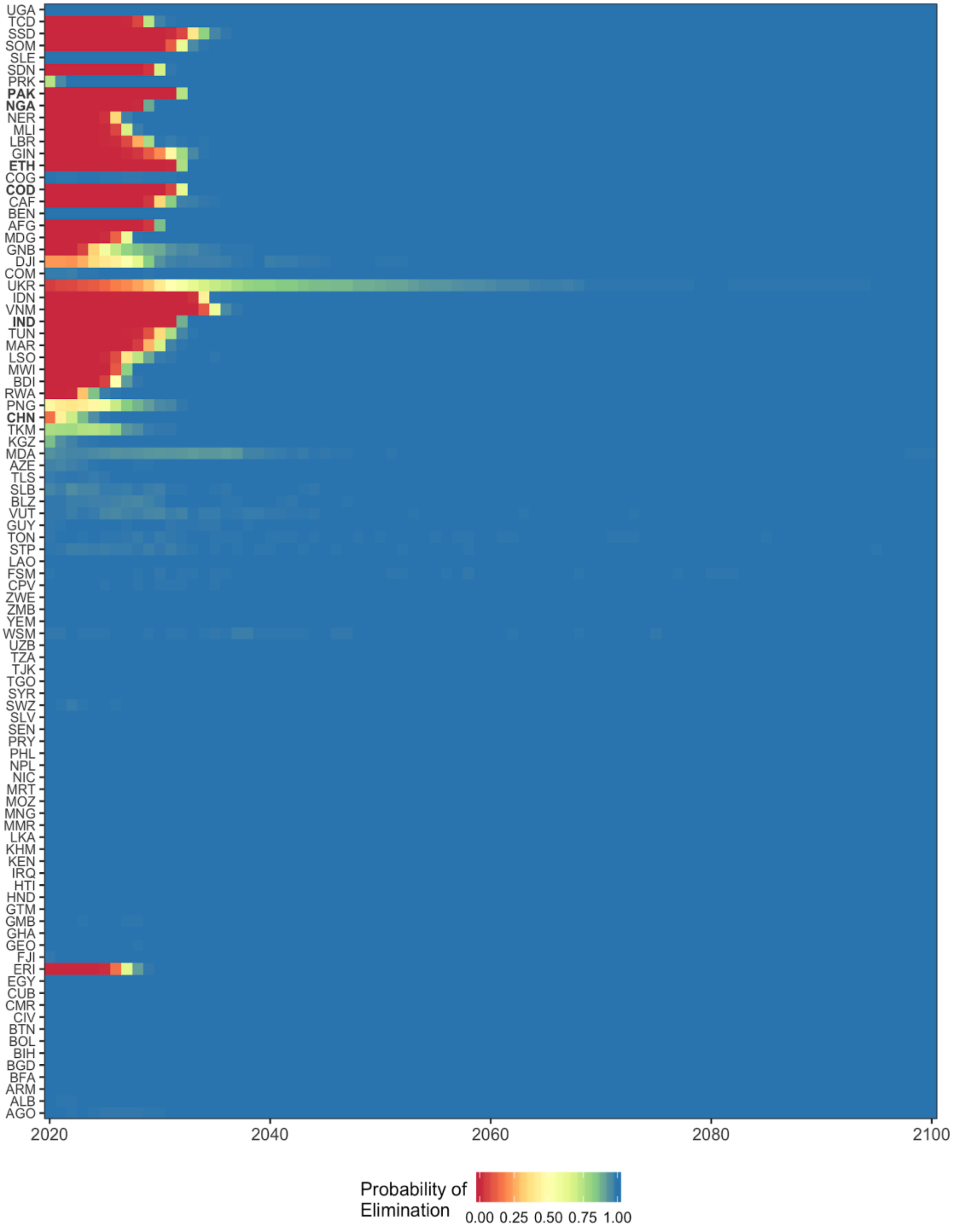

Figure S9: Probability of rubella elimination, JHU, intensified-investment.

JHU model estimates of the annual probability of reaching the "elimination" threshold of 5 rubella cases per one million population, shown as the proportion of 200 stochastic runs which reach the threshold in the **intensified-investment** vaccination scenario. Countries are listed according to their International Organization for Standardization 3166 alpha-3 country codes (ISO 3; https://www.iso.org/iso-3166-country-codes.html).

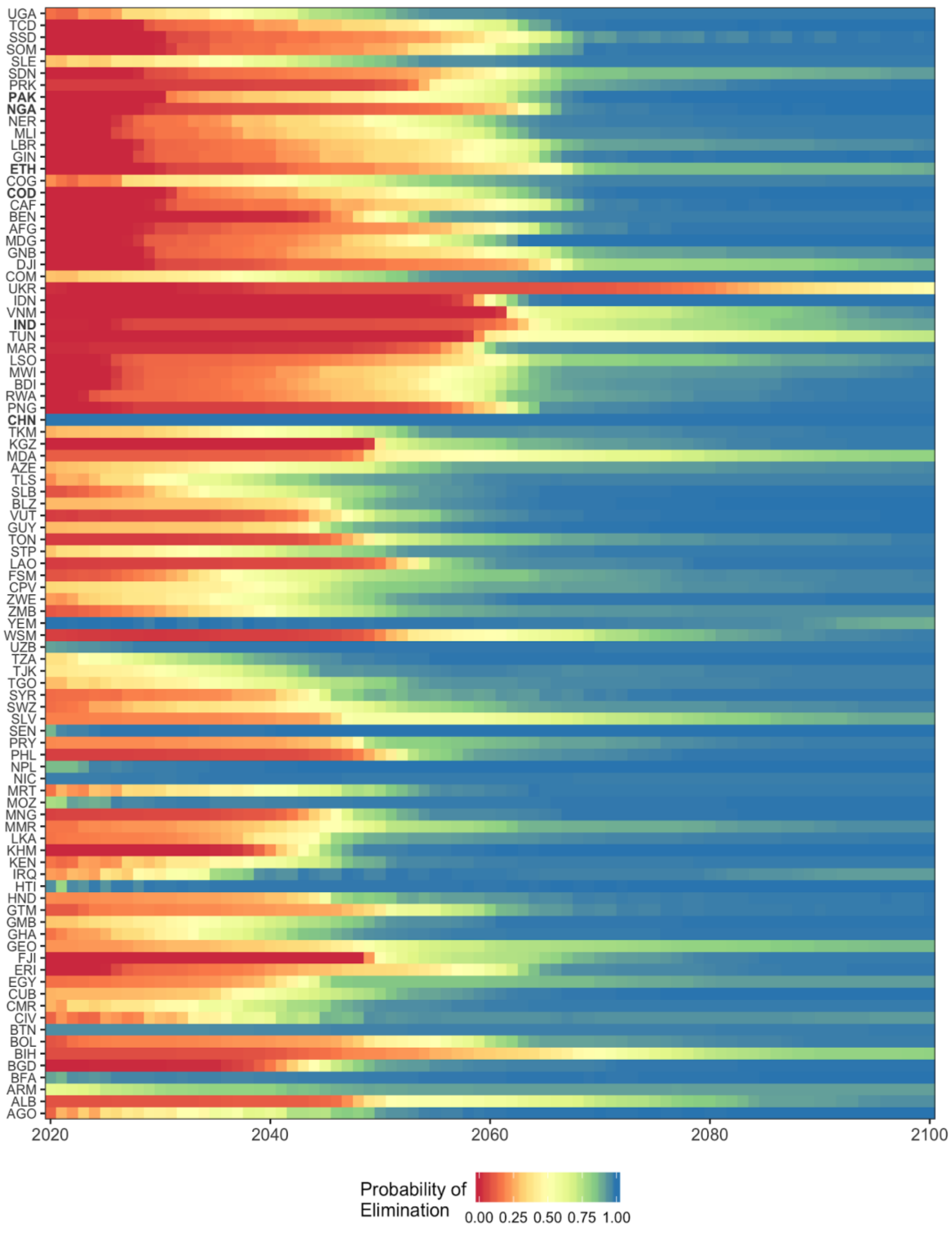

Figure S10: Probability of rubella elimination, PHE, intensified-investment.

PHE model estimates of the annual probability of reaching the "elimination" threshold of 5 rubella cases per one million population, shown as the proportion of 200 stochastic runs which reach the threshold in the **intensified-investment** vaccination scenario. Countries are listed according to their International Organization for Standardization 3166 alpha-3 country codes (ISO 3; https://www.iso.org/iso-3166-country-codes.html).

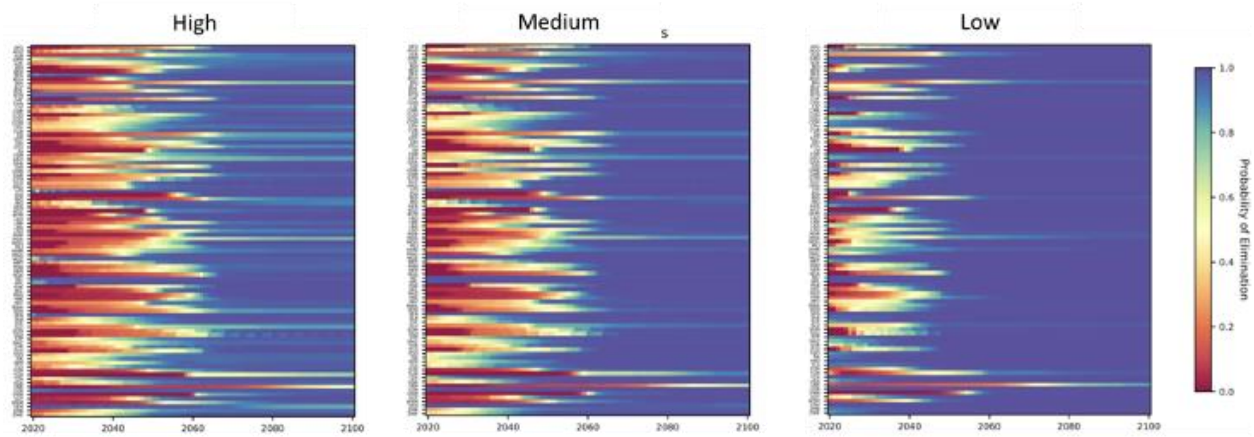

Figure S11: Sensitivity Analysis, PHE.

PHE model predictions of achieving an annual incidence of 5 per million for the intensified-investment scenario for assumptions of a high, medium, and low importation rate, defined as 0.01%, 0.005%, and 0.001% respectively of susceptibles infected abroad monthly. Note that the countries are ordered alphabetically from top to bottom. Countries as listed according to their International Organization for Standardization 3166 alpha-3 country codes (ISO 3; https://www.iso.org/iso-3166-country-codes.html).

Measles Results

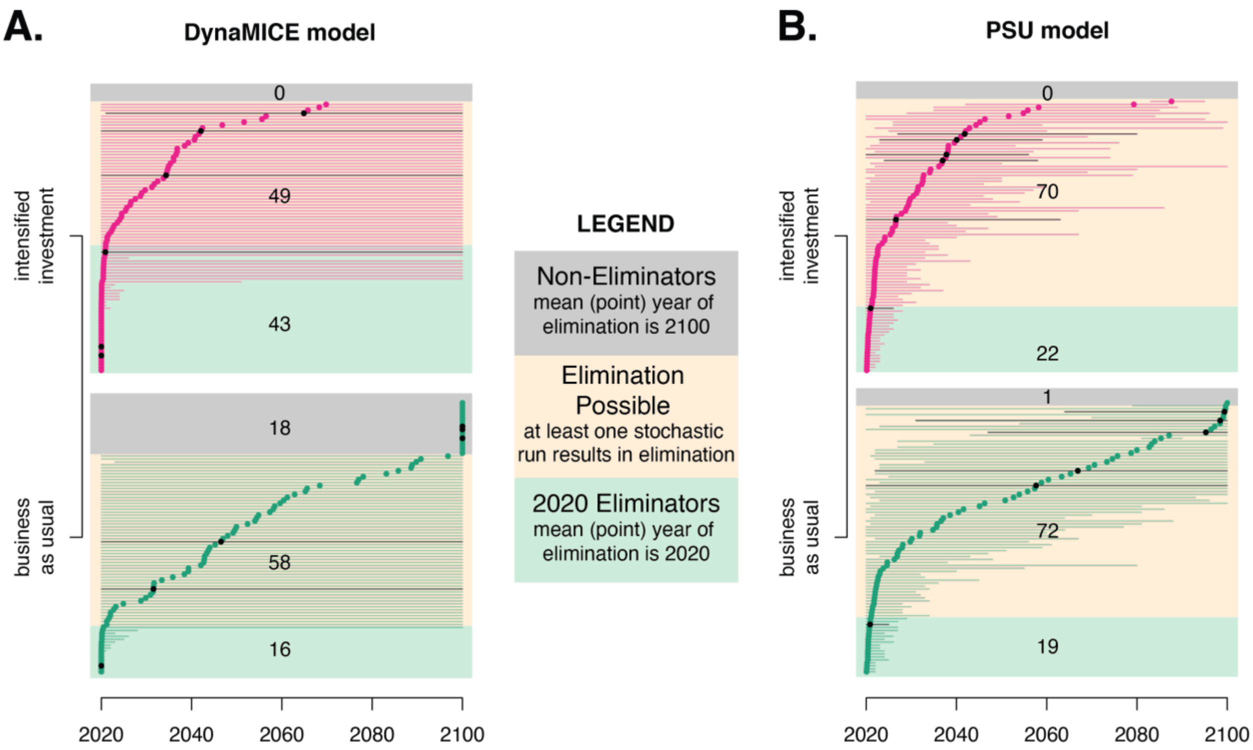

Figure S12: Time to measles elimination.

The year that countries reach the "elimination" threshold of 5 measles cases per one million population for DynaMICE (**A**) and PSU (**B**) models in business-as-usual (green) and intensified-investment (pink) vaccination scenarios. The point represents the mean year, and the bars show the minimum and maximum years from 200 stochastic runs for each country. Simulations that never result in cases below the elimination threshold are assigned the elimination year 2100. The black points and lines represent Pakistan, India, Nigeria, Ethiopia, and the Democratic Republic of Congo (DRC). Countries are ordered by the mean year they reach elimination, and the order will differ by scenario and model. The transparent green, tan, and grey boxes characterize countries as 2020 eliminators, elimination possible (although potentially improbable), and non-eliminators, respectively.

	Observed Elimination	Observed Non-Elimination	Total
**DynaMICE Simulated Elimination**	10 (11.1%)<br>*200,444 (3.7%)*	6 (6.7%)<br>*185,148 (3.5%)*	16
**DynaMICE Simulated Non-Elimination**	57 (63.3%)<br>*3,857,707 (72.0%)*	17 (18.9%)<br>*1,117,639 (20.8%)*	74
**Total**	67	23	90 (100%)<br>*5,360,938 (100%)*

Table S3: Distribution of countries by observed and DynaMICE measles simulated elimination status as of 2020.

DynaMICE simulated 'elimination' are countries that reach the "elimination" threshold of 5 measles infections per one million population by mean year of 2020 (i.e., 2017-2020 across 200 simulated runs) given **business-as-usual** vaccination scenarios. Observed 'elimination' are countries that reported annual < 5 measles per one million population between 2017 and 2020 to the WHO via Joint Reporting Form. Note that 3 countries did not report cases (including zero cases) 2017-2020 to the WHO and were therefore were not included in the table. The top row (black text) represents the number of countries (percentage); the bottom row (blue italic text) represents the 2020 population total (percentage).

	Observed Elimination	Observed Non-Elimination	Total
**PSU Simulated Elimination**	18 (20%)<br>*1,736,038 (32.4%)*	1 (1.1%)<br>*12,952 (0.2%)*	19
**PSU Simulated Non-Elimination**	49 (54.4%)<br>*2,322,113 (43.3%)*	22 (24.4%)<br>*1,289,835 (24.1%)*	71
**Total**	67	23	90 (100%)<br>*5,360,938 (100%)*

Table S4: Distribution of countries by observed and PSU measles simulated elimination status as of 2020.

PSU simulated 'elimination' are countries that reach the "elimination" threshold of 5 measles infections per one million population by mean year of 2020 (i.e., 2017-2020 across 200 simulated runs) given **business-as-usual** vaccination scenarios. Observed 'elimination' are countries that reported annual < 5 measles per one million population between 2017 and 2020 to the WHO via Joint Reporting Form. Note that 3 countries did not report cases (including zero cases) 2017-2020 to the WHO and were therefore were not included in the table. The top row (black text) represents the number of countries (percentage); the bottom row (blue italic text) represents the 2020 population total (percentage).

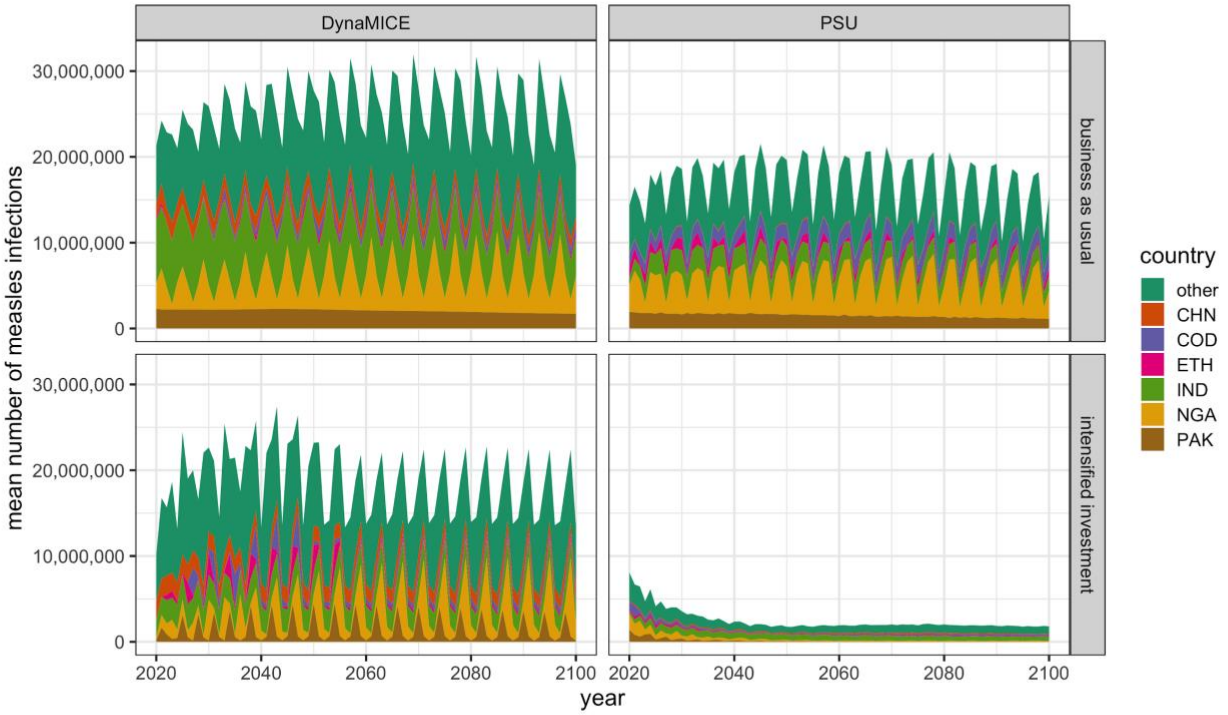

Figure S13: Annual measles infections.

The mean number of annual measles infections for China (CHN), Democratic Republic of Congo (COD), Ethiopia (ETH), India (IND), Nigeria (NGA), Pakistan (PAK), and all other countries combined (other) per DynaMICE (left column) and PSU (right column) models for business-as-usual (top row) and intensified-investment (bottom row) vaccination scenarios.

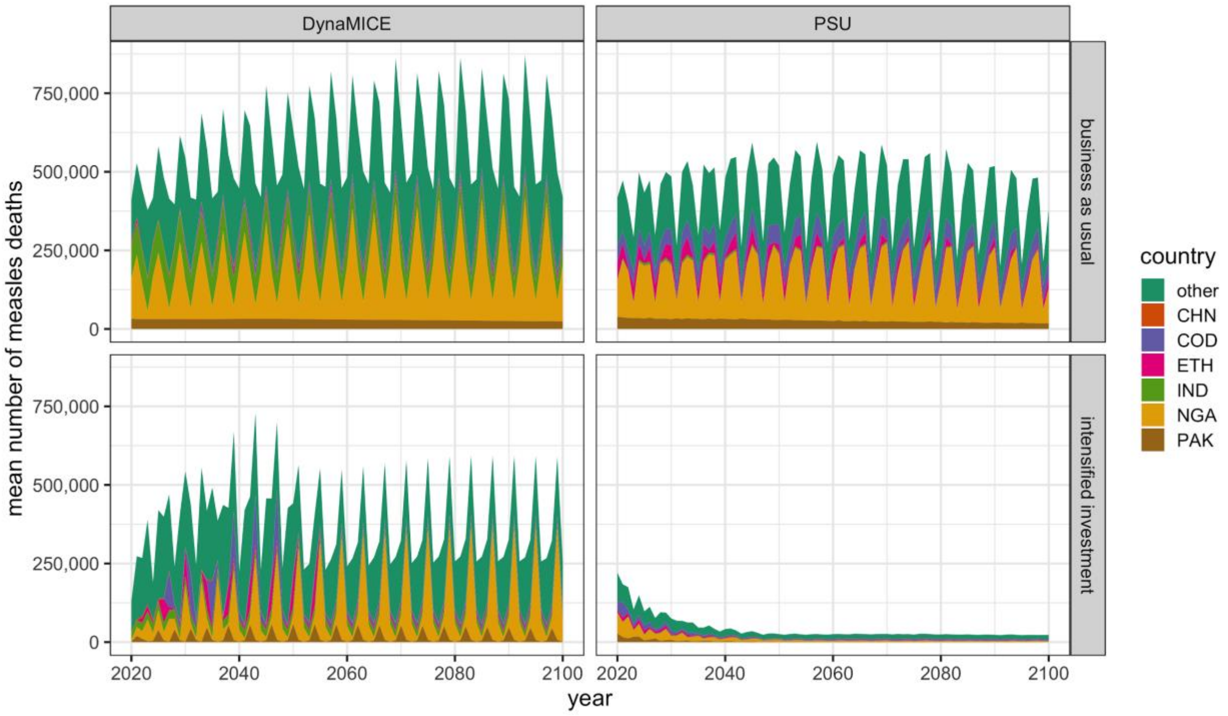

Figure S14: Annual measles deaths.

The mean number of annual measles deaths for China (CHN), Democratic Republic of Congo (COD), Ethiopia (ETH), India (IND), Nigeria (NGA), Pakistan (PAK), and all other countries combined (other) per DynaMICE (left column) and PSU (right column) models for business-as-usual (top row) and intensified-investment (bottom row) vaccination scenarios.

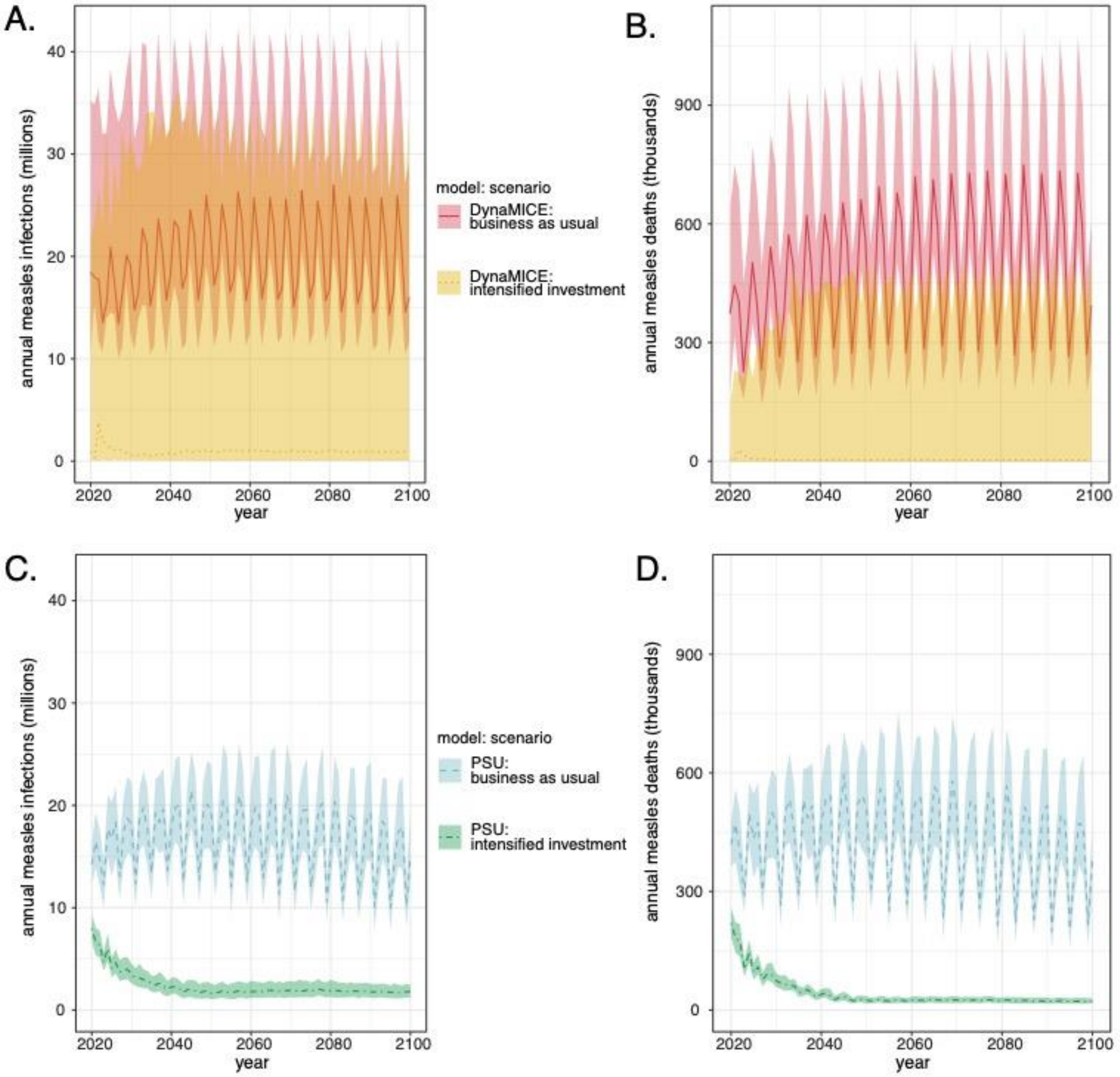

Figure S15: Measles burden with model uncertainty.

Time series of the annual aggregate number of measles infections and deaths across 93 countries based on London School of Hygiene & Topical Medicine (DynaMICE) (A,B) and Pennsylvania State University (PSU) (C,D) models given business-as-usual and intensified-investment vaccination scenarios. The line (i.e., color and line type) and ribbon (i.e., transparent shading) for each model and scenario represent the median and 80% prediction intervals across 200 stochastic runs.

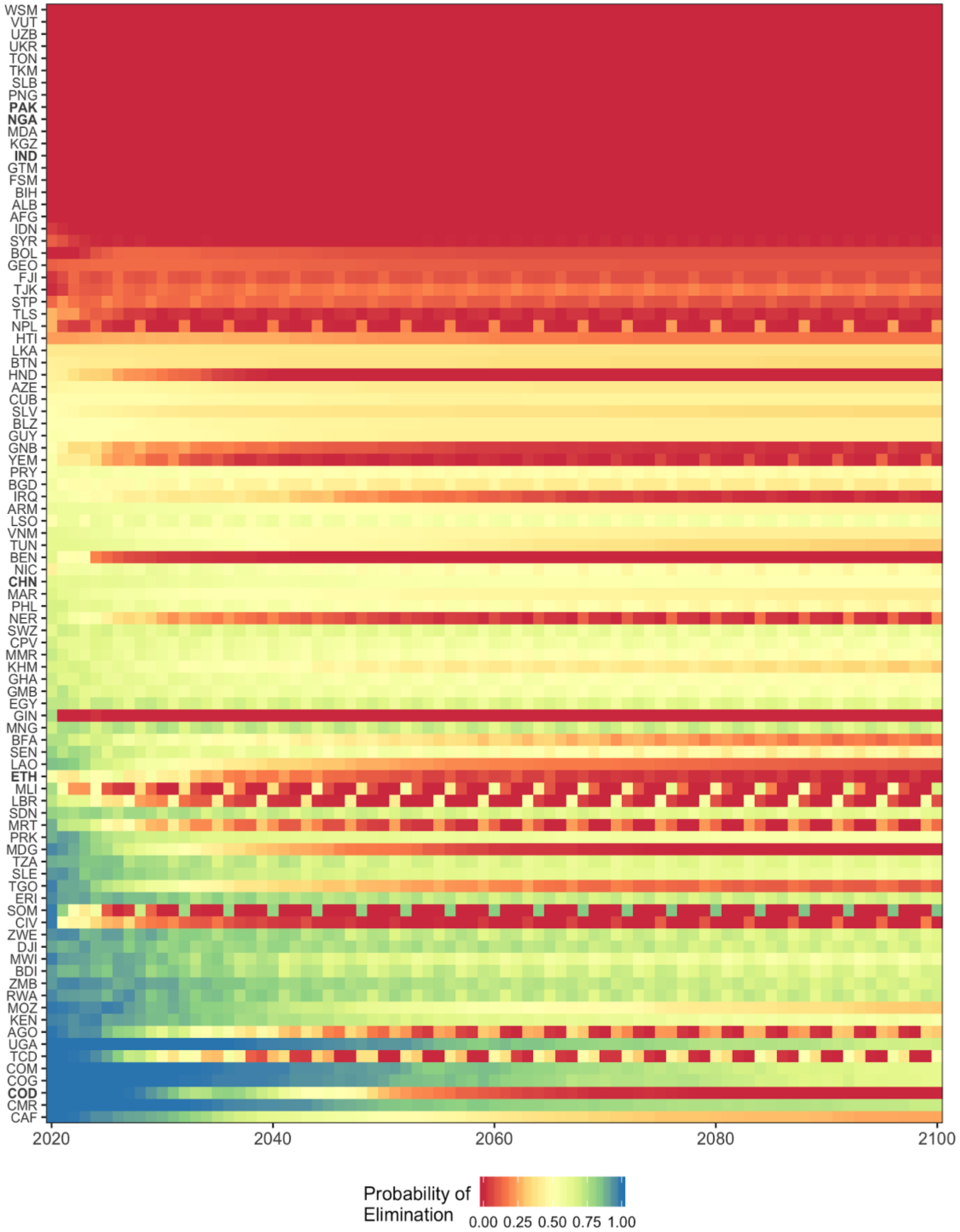

Figure S16: Probability of measles elimination, DynaMICE, business-as-usual.

DynaMICE model estimates of the annual probability of reaching the "elimination" threshold of 5 measles cases per one million population, shown as the proportion of 200 stochastic runs which reach the threshold in the **business-as-usual** vaccination scenario. Countries are listed according to their International Organization for Standardization 3166 alpha-3 country codes (ISO 3; https://www.iso.org/iso-3166-country-codes.html).

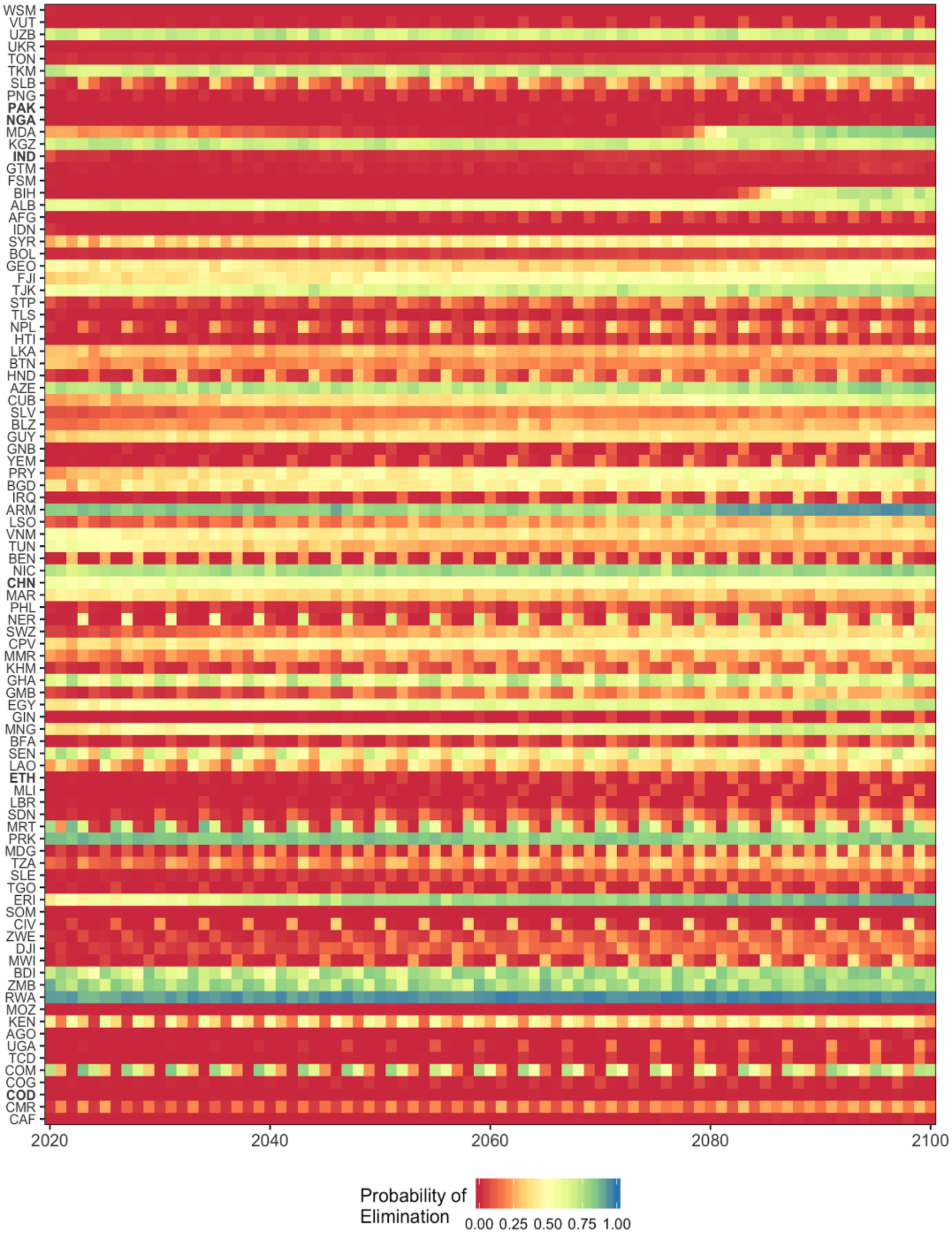

Figure S17: Probability of measles elimination, PSU, business-as-usual.

PSU model estimates of the annual probability of reaching the "elimination" threshold of 5 measles cases per one million population, shown as the proportion of 200 stochastic runs which reach the threshold in the **business-as-usual** vaccination scenario. Countries are listed according to their International Organization for Standardization 3166 alpha-3 country codes (ISO 3; https://www.iso.org/iso-3166-country-codes.html).

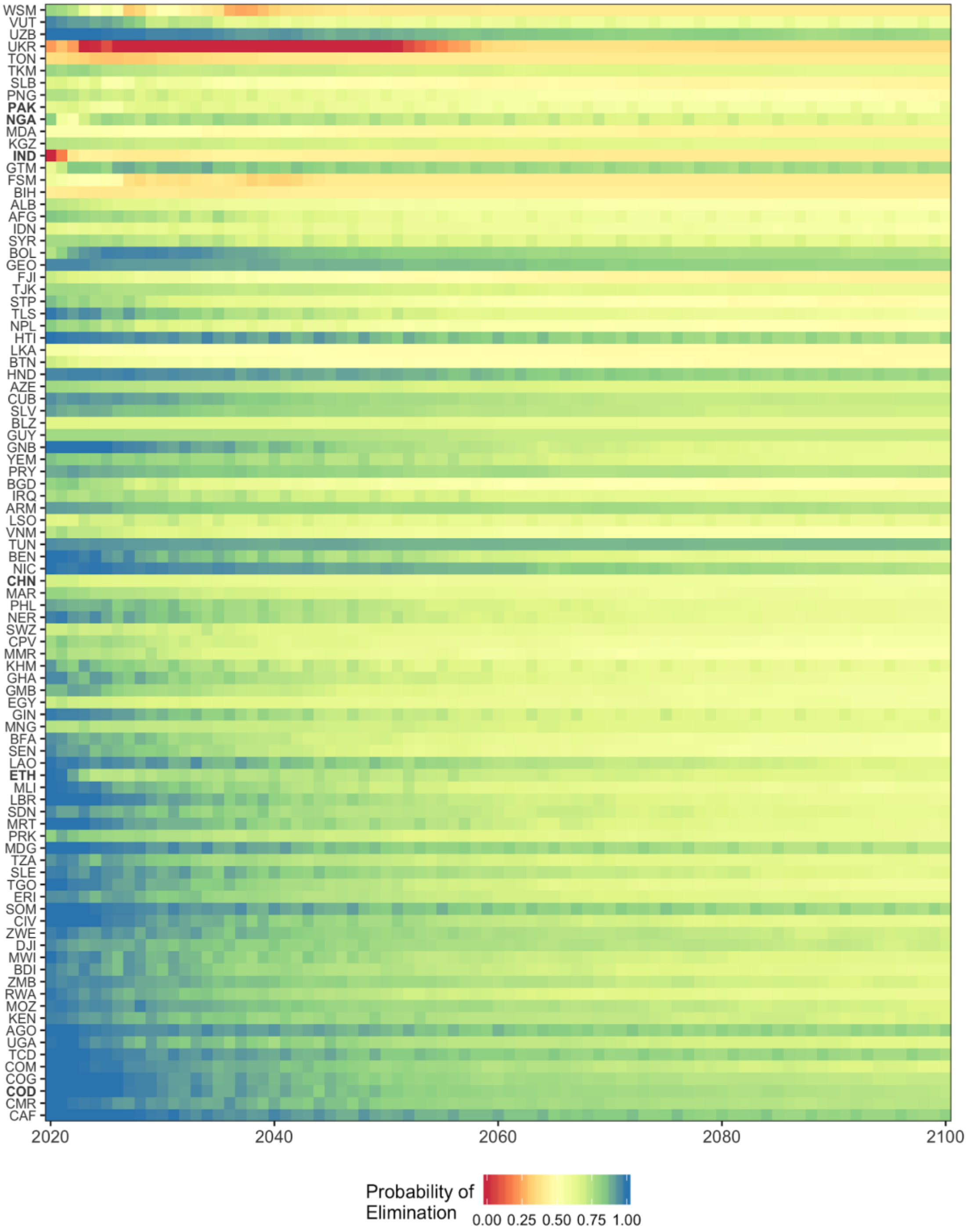

Figure S18: Probability of measles elimination, DynaMICE, intensified-investment.

DynaMICE model estimates of the annual probability of reaching the “elimination” threshold of 5 measles cases per one million population, shown as the proportion of 200 stochastic runs which reach the threshold in the **intensified-investment** vaccination scenario. Countries are listed according to their International Organization for Standardization 3166 alpha-3 country codes (ISO 3; https://www.iso.org/iso-3166-country-codes.html).

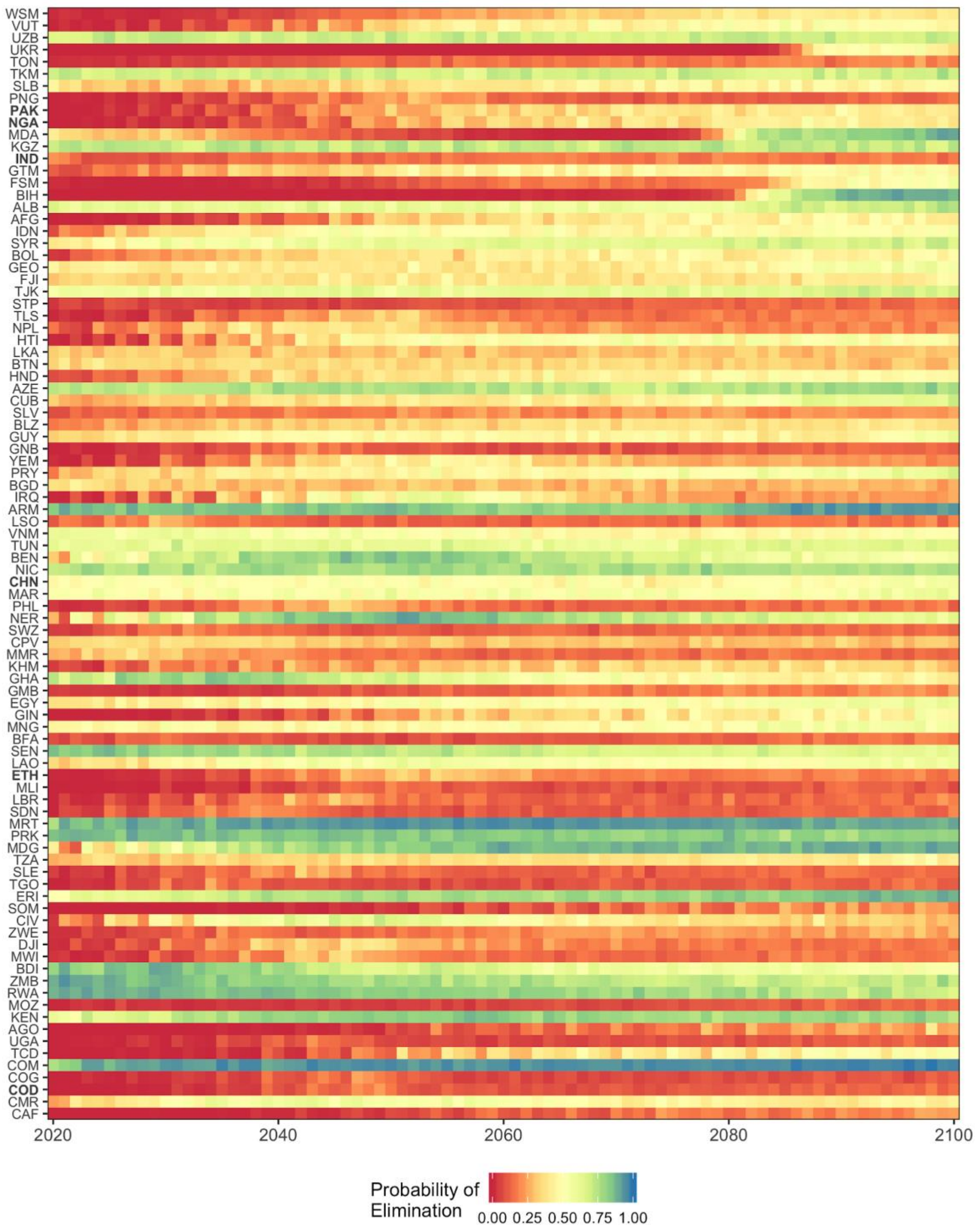

Figure S19: Probability of measles elimination, PSU, intensified-investment.

PSU model estimates of the annual probability of reaching the “elimination” threshold of 5 measles cases per one million population, shown as the proportion of 200 stochastic runs which reach the threshold in the **intensified-investment** vaccination scenario. Countries are listed according to their International Organization for Standardization 3166 alpha-3 country codes (ISO 3; https://www.iso.org/iso-3166-country-codes.html).

Measles Results - Subnational Model

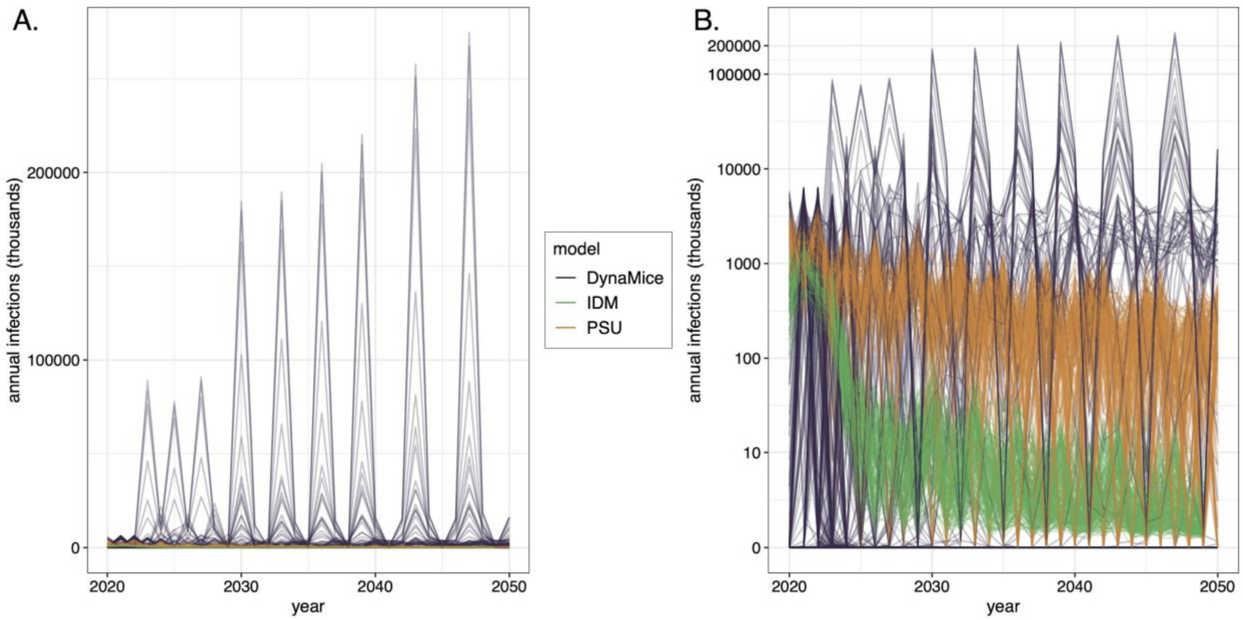

Figure S20: Time series of measles infections in Nigeria.

Time series of incident measles infections in Nigeria based on the DynaMICE national model (purple lines), IDM sub-national model (green lines), and PSU national model (orange lines) under the intensified-investment vaccination scenario. Each line represents one stochastic simulation, 200 total for each model. Figure **B** y-axis is log base 10 annual infections in thousands with a smooth transition to linear scale around 0.

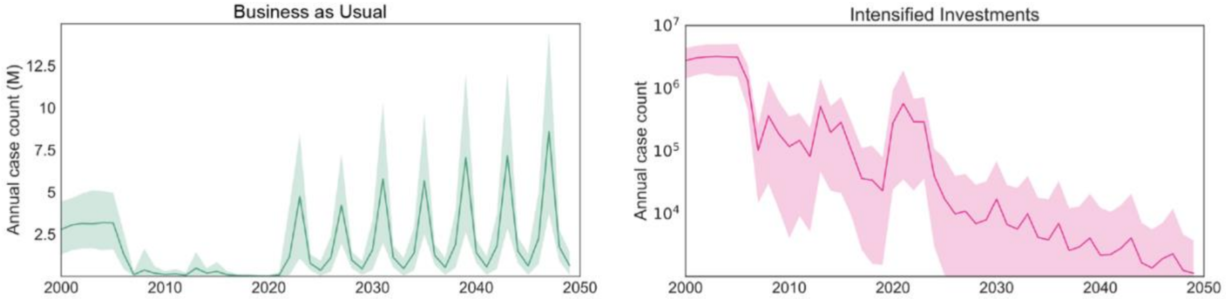

Figure S21: Annual measles infections in Nigeria, IDM.

Annual numbers of measles infections for Nigeria for the IDM sub-national model, in the **business-as-usual** (left) and **intensified-investment** (right) scenarios.

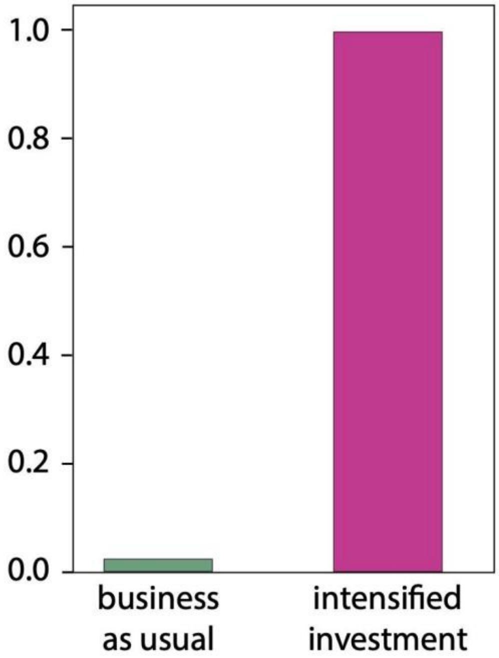

Figure S22: Probability of measles elimination in Nigeria, IDM.

Probability of reaching the "elimination" threshold of 5 measles cases per one million population, shown as the proportion of stochastic runs that reach the threshold by vaccination scenario in the IDM sub-national model.

Results (in greyscale)

Rubella Results

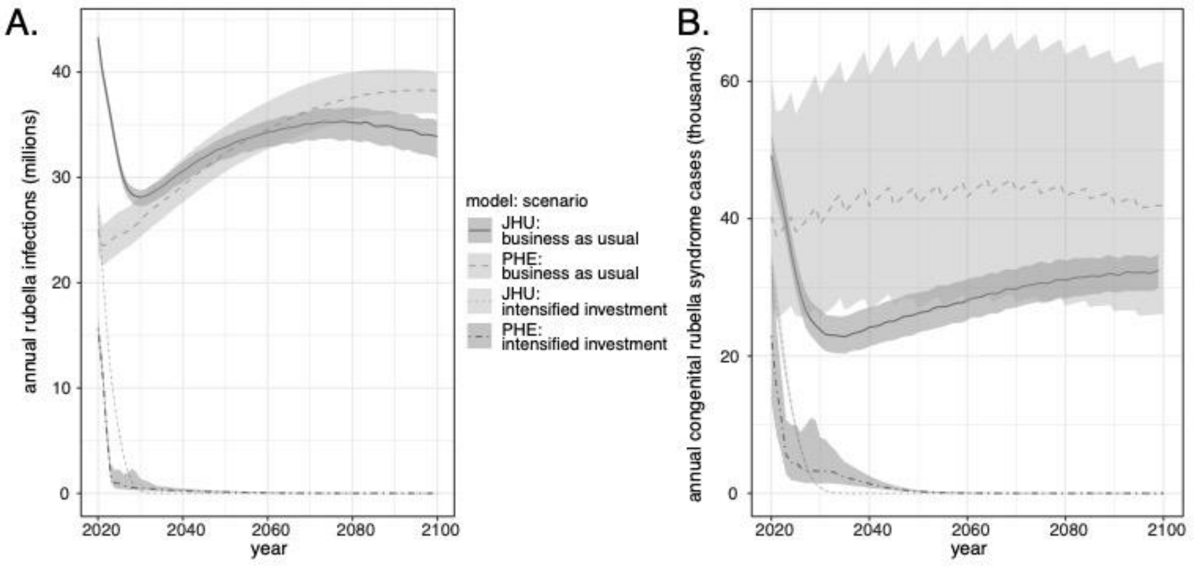

Figure S2: Rubella burden with model uncertainty.

Time series of the annual aggregate number of rubella infections (A) and CRS cases (B) across 93 countries based on Johns Hopkins University (JHU) and Public Health England (PHE) models given business-as-usual and intensified-investment vaccination scenarios. The line (i.e., color and line type) and ribbon (i.e., transparent shading) for each model and scenario represent the median and 80% prediction intervals across 200 stochastic runs.

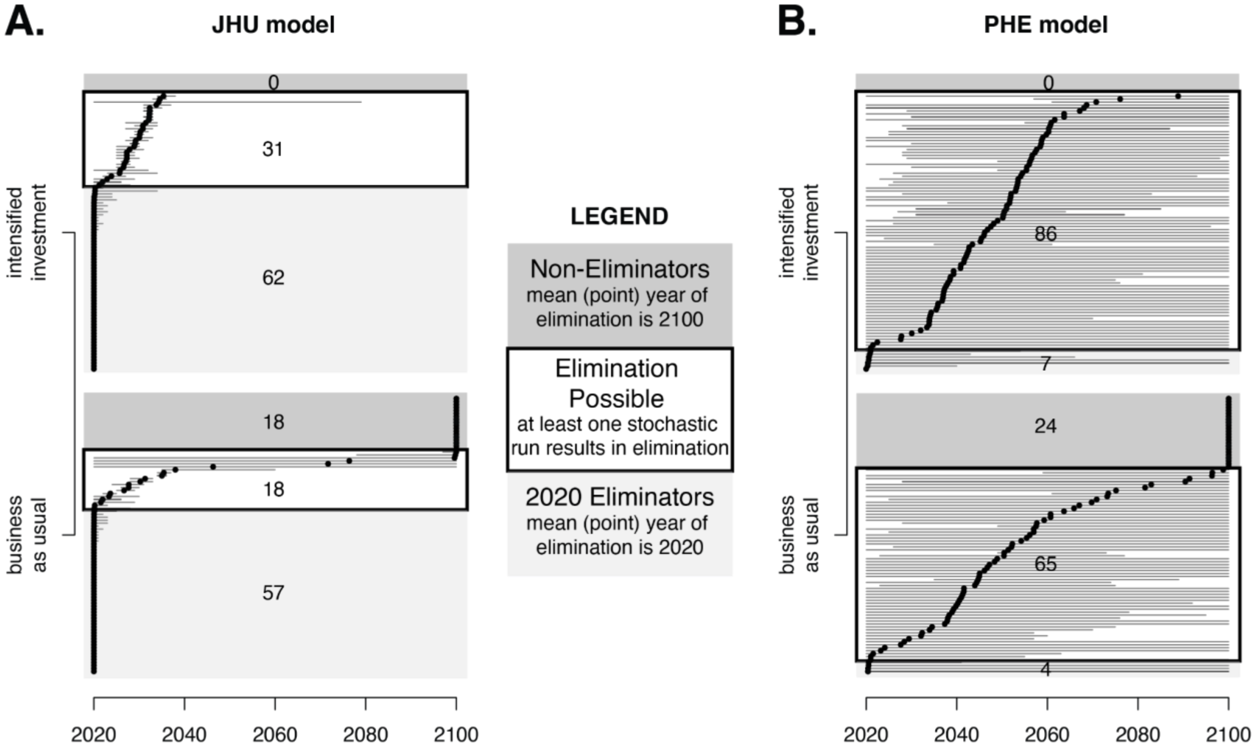

Figure S3*: *Time to rubella elimination.

The year that countries reach the "elimination" threshold of 5 rubella cases per one million population for JHU (**A**) and PHE (**B**) models for business-as-usual (green) and intensified-investment (pink) vaccination scenarios. The point represents the mean, and the bars show the minimum and maximum values from 200 runs. The black points and lines represent Pakistan, India, Nigeria, Ethiopia, and the Democratic Republic of Congo (DRC). Simulations that never result in cases below the elimination threshold are assigned the elimination year 2100. Countries are ordered by the mean year they reach elimination and order will differ by scenario and model. The transparent green, tan, and grey boxes characterize countries as 2020 eliminators, elimination possible, and non-eliminators, respectively.

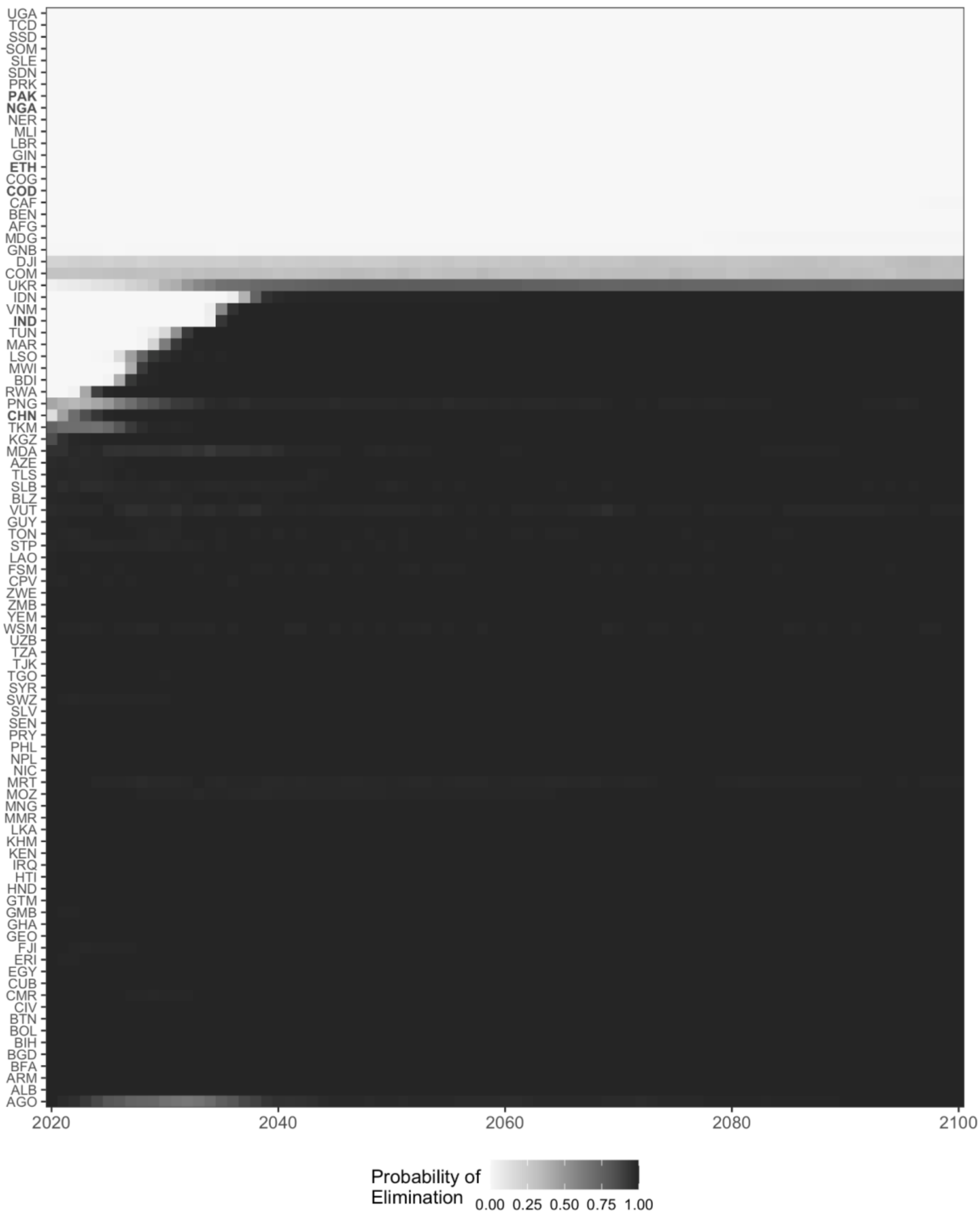

Figure S4: Probability of rubella elimination, JHU, business-as-usual.

JHU model estimates of the annual probability of reaching the "elimination" threshold of 5 rubella cases per one million population, shown as the proportion of 200 stochastic runs which reach the threshold in the **business-as-usual** vaccination scenario. Countries are listed according to their International Organization for Standardization 3166 alpha-3 country codes (ISO 3; https://www.iso.org/iso-3166-country-codes.html).

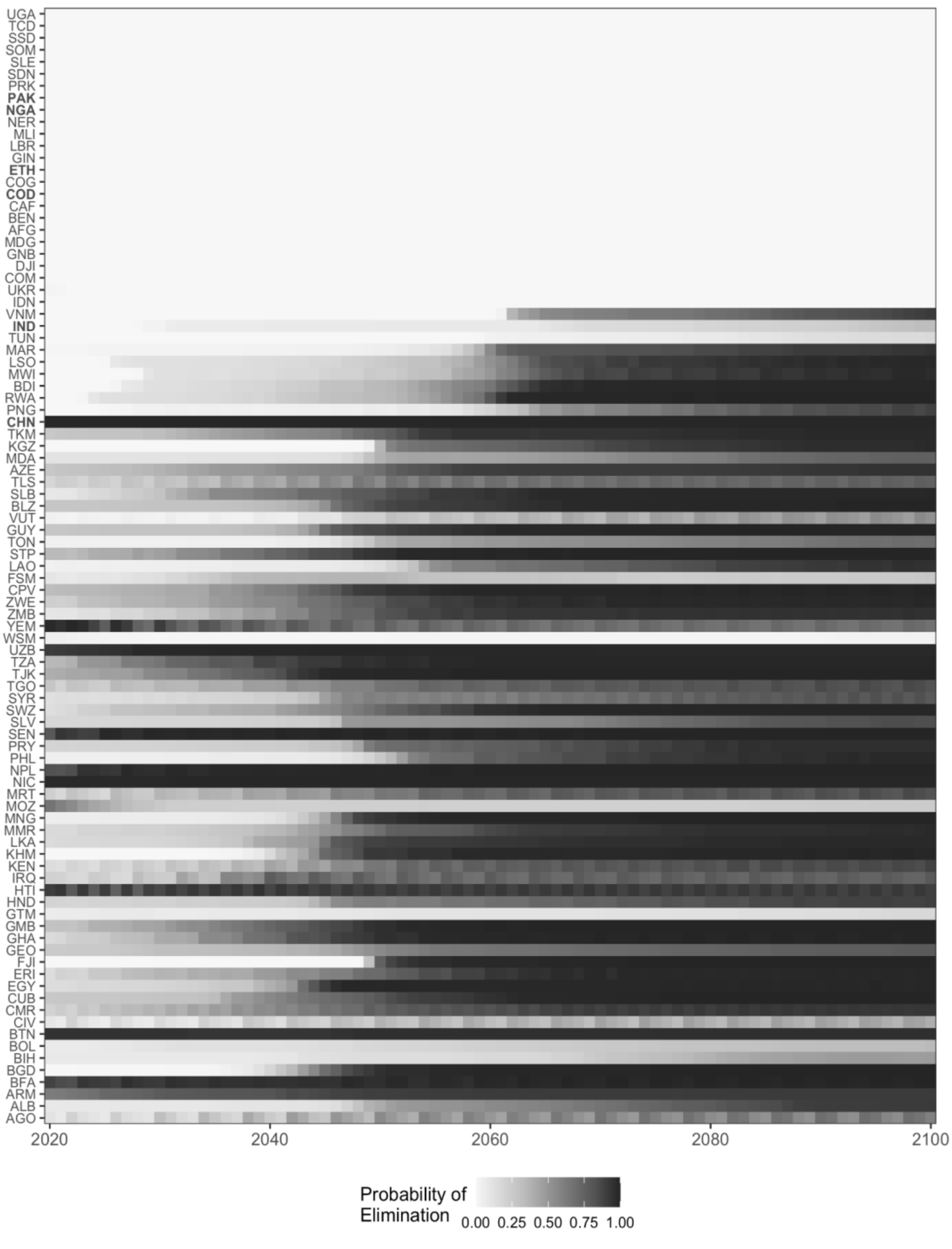

Figure S5: Probability of rubella elimination, PHE, business-as-usual.

PHE model estimates of the annual probability of reaching the “elimination” threshold of 5 rubella cases per one million population, shown as the proportion of 200 stochastic runs which reach the threshold in the **business-as-usual** vaccination scenario. Countries are listed according to their International Organization for Standardization 3166 alpha-3 country codes (ISO 3; https://www.iso.org/iso-3166-country-codes.html).

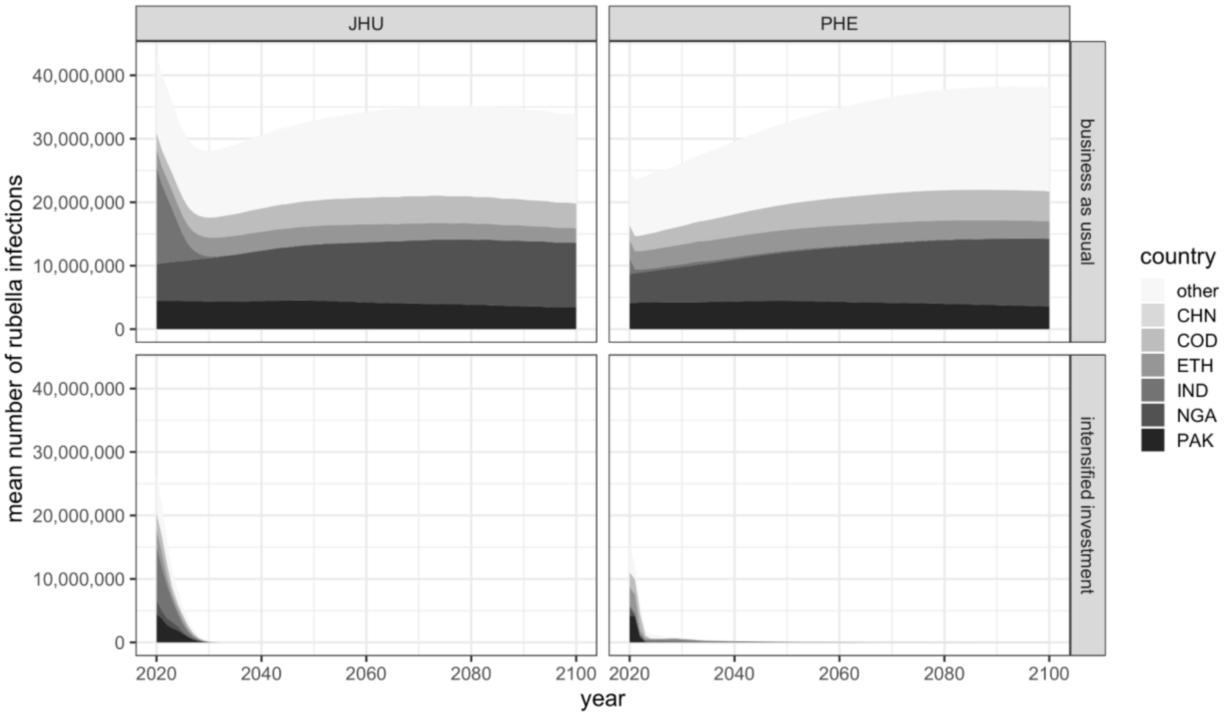

Figure S6: Annual rubella infections.

The mean number of annual rubella infections for China (CHN), Democratic Republic of Congo (COD), Ethiopia (ETH), India (IND), Nigeria (NGA), Pakistan (PAK), and all other countries combined (other) per JHU (left column) and PHE (right column) models for the business-as-usual (top row) and intensified-investment (bottom row) vaccination scenarios.

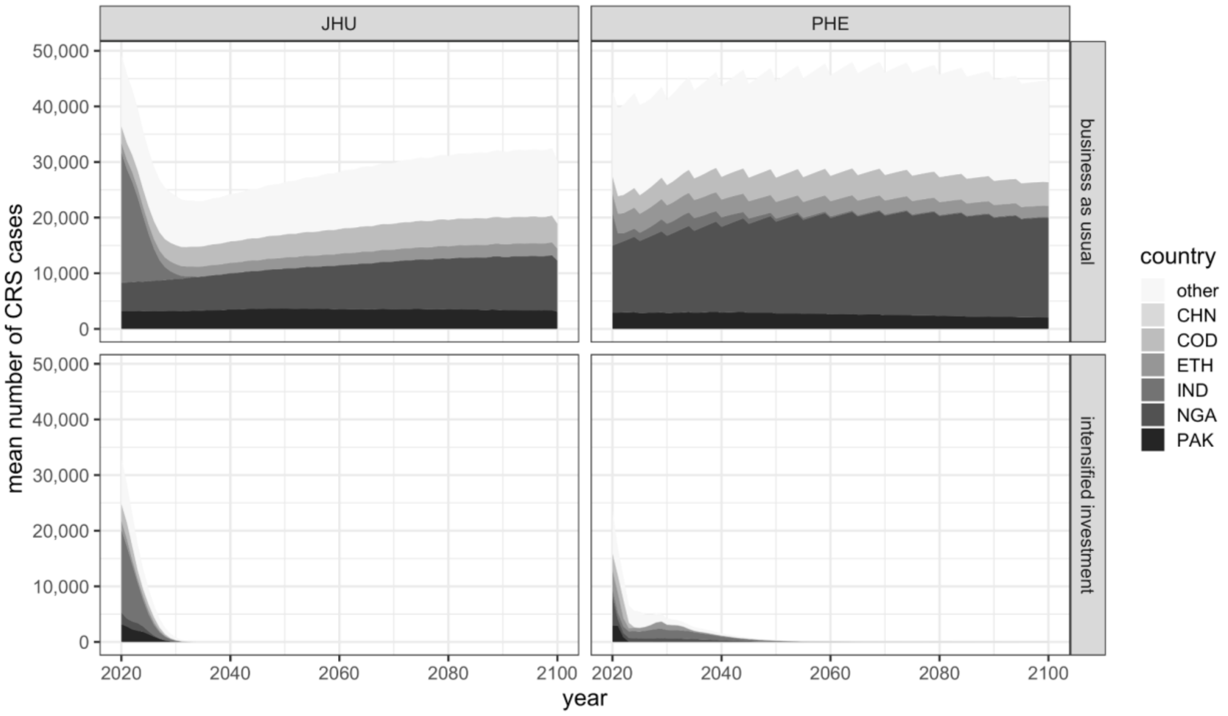

Figure S7: Annual CRS cases.

The mean number of annual CRS cases for China (CHN), Democratic Republic of Congo (COD), Ethiopia (ETH), India (IND), Nigeria (NGA), Pakistan (PAK), and all other countries combined (other) per JHU (left column) and PHE (right column) models for the business-as-usual (top row) and intensified-investment (bottom row) vaccination scenarios.

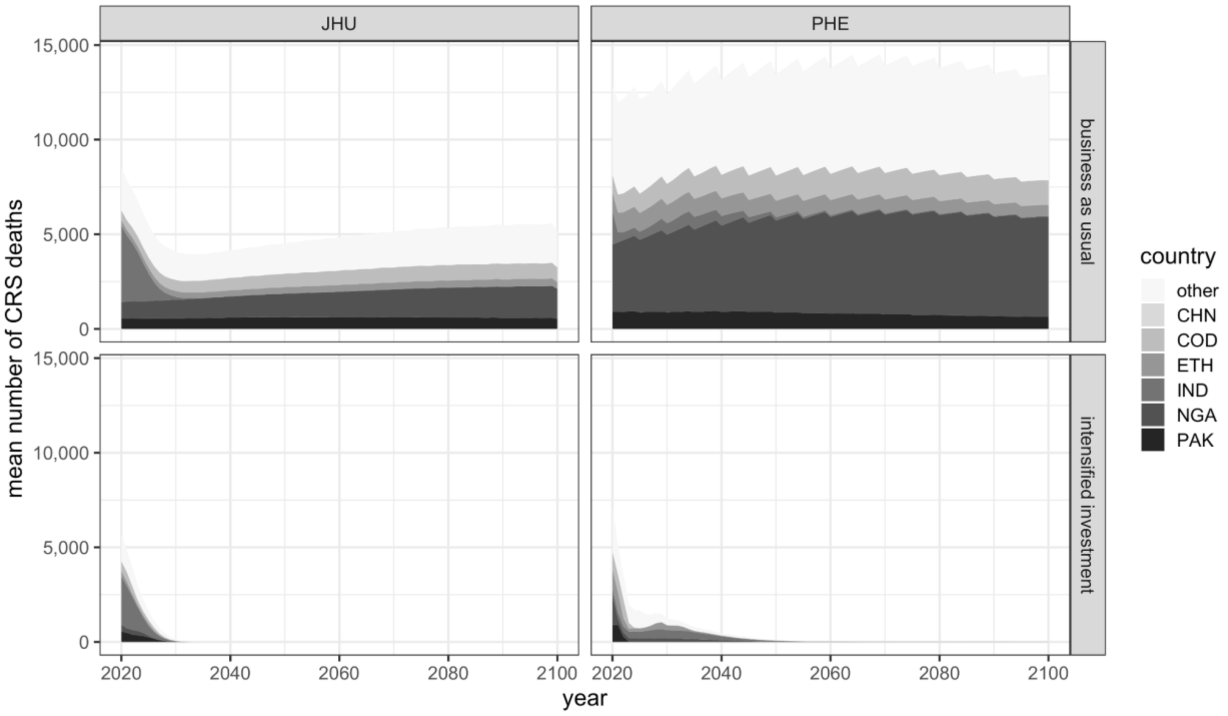

Figure S8: Annual CRS deaths.

The mean number of annual deaths due to CRS for China (CHN), Democratic Republic of Congo (COD), Ethiopia (ETH), India (IND), Nigeria (NGA), Pakistan (PAK), and all other countries combined (other) per JHU (left column) and PHE (right column) models for the business-as-usual (top row) and intensified-investment (bottom row) vaccination scenarios.

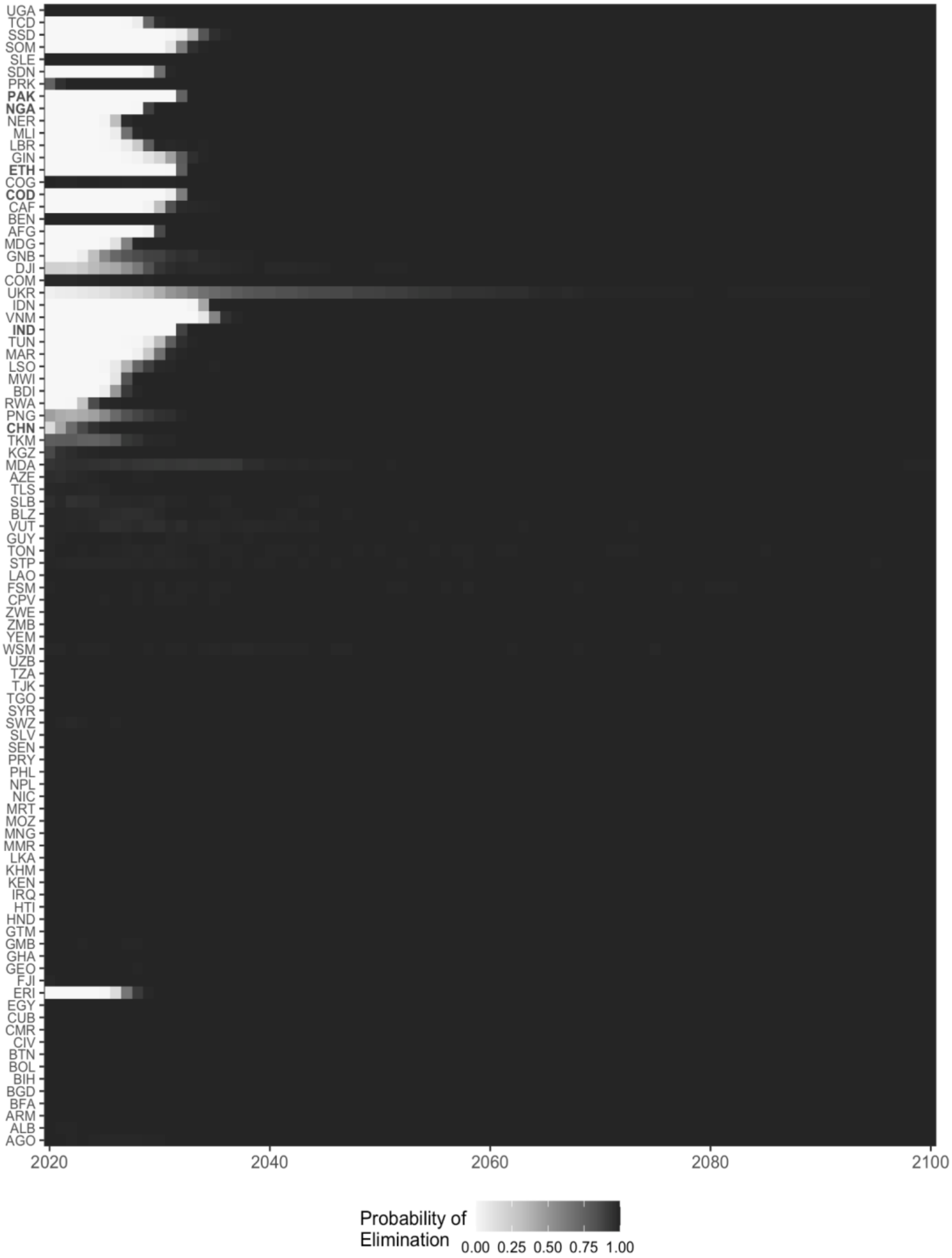

Figure S9: Probability of rubella elimination, JHU, intensified-investment.

JHU model estimates of the annual probability of reaching the “elimination” threshold of 5 rubella cases per one million population, shown as the proportion of 200 stochastic runs which reach the threshold in the **intensified-investment** vaccination scenario. Countries are listed according to their International Organization for Standardization 3166 alpha-3 country codes (ISO 3; https://www.iso.org/iso-3166-country-codes.html).

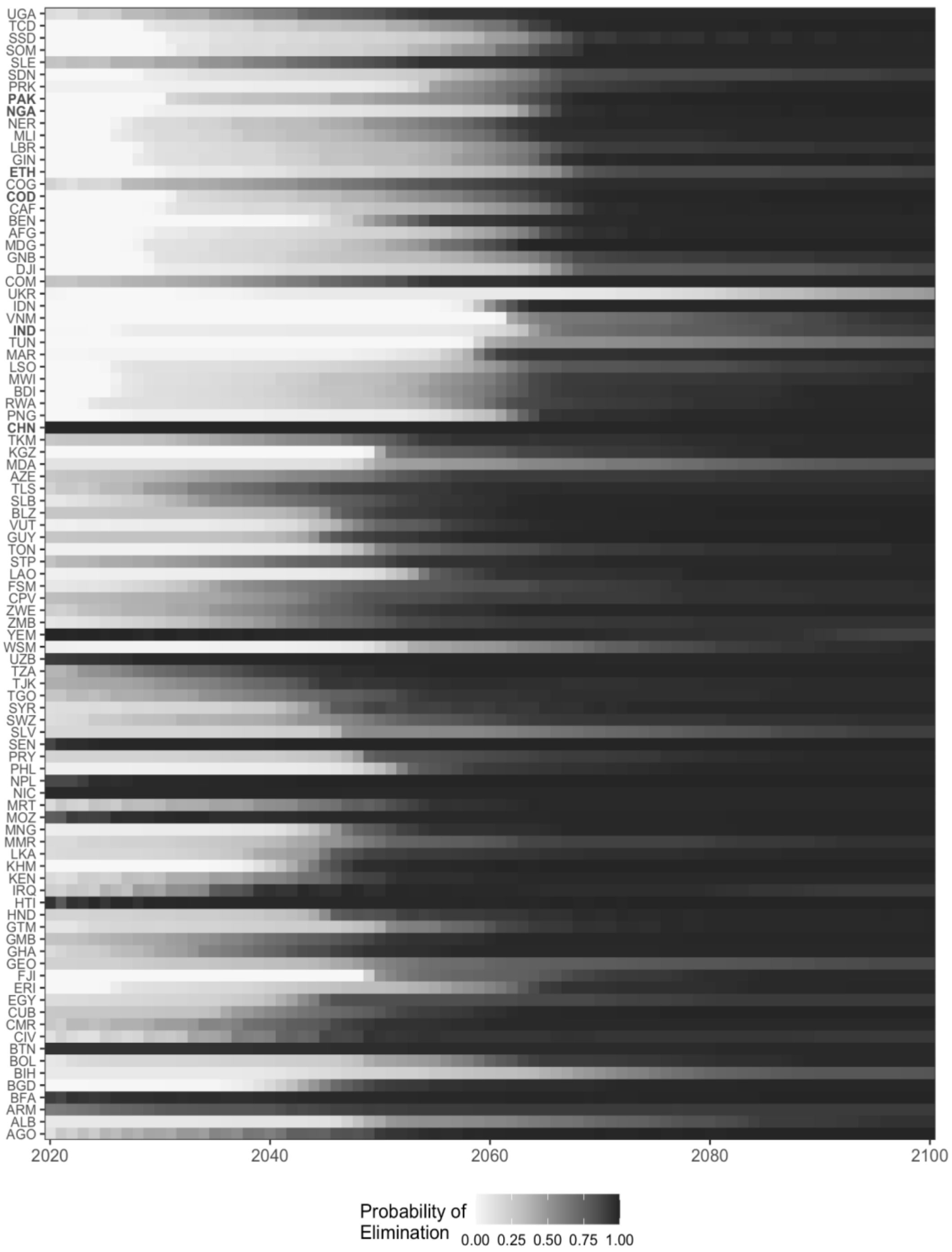

Figure S10: Probability of rubella elimination, PHE, intensified-investment.

PHE model estimates of the annual probability of reaching the "elimination" threshold of 5 rubella cases per one million population, shown as the proportion of 200 stochastic runs which reach the threshold in the **intensified-investment** vaccination scenario. Countries are listed according to their International Organization for Standardization 3166 alpha-3 country codes (ISO 3; https://www.iso.org/iso-3166-country-codes.html).

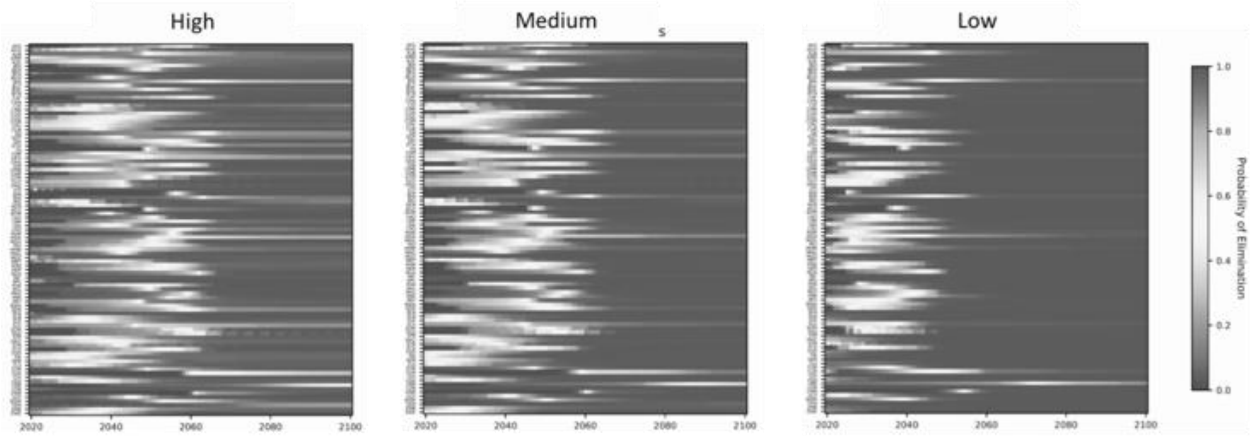

Figure S11: Sensitivity Analysis, PHE.

PHE model predictions of achieving an annual incidence of 5 per million for the intensified-investment scenario for assumptions of a high, medium, and low importation rate, defined as 0.01%, 0.005%, and 0.001% respectively of susceptibles infected abroad monthly. Note that the countries are ordered alphabetically from top to bottom. Countries as listed according to their International Organization for Standardization 3166 alpha-3 country codes (ISO 3; https://www.iso.org/iso-3166-country-codes.html).

Measles Results

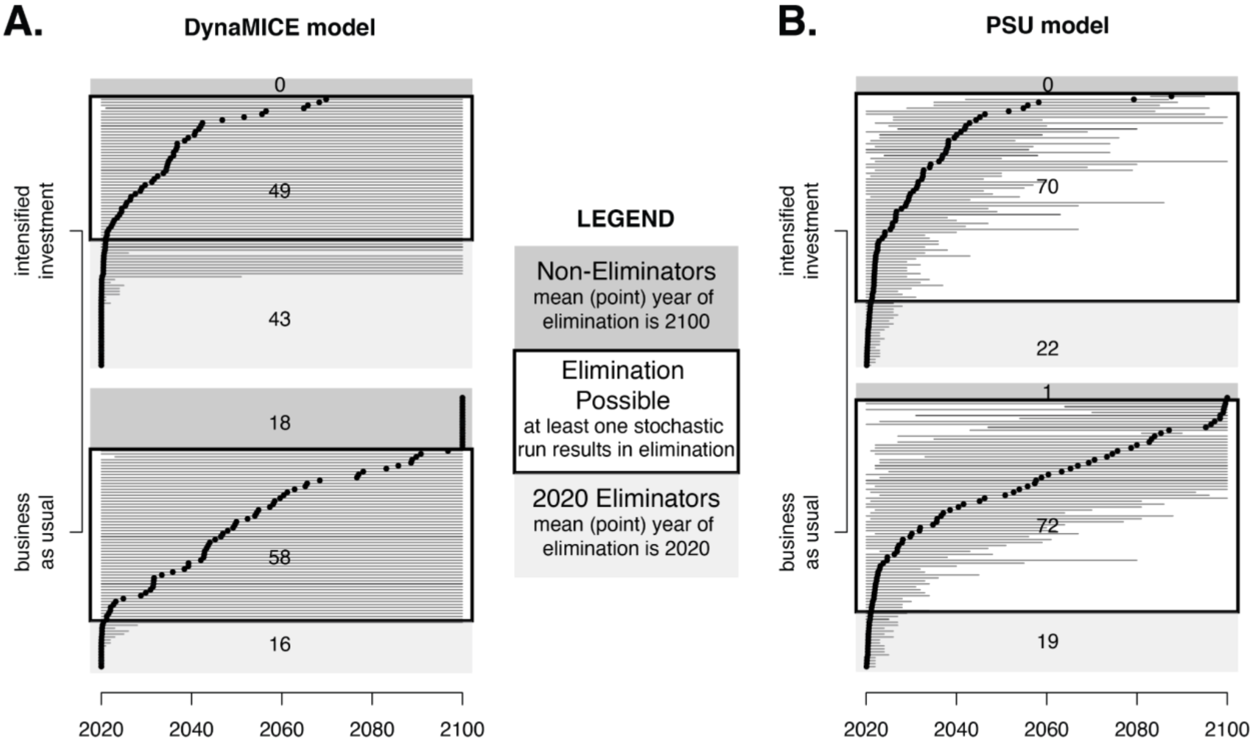

Figure S12: Time to measles elimination.

The year that countries reach the "elimination" threshold of 5 measles cases per one million population for DynaMICE (**A**) and PSU (**B**) models in business-as-usual (green) and intensified-investment (pink) vaccination scenarios. The point represents the mean year, and the bars show the minimum and maximum years from 200 stochastic runs for each country. Simulations that never result in cases below the elimination threshold are assigned the elimination year 2100. The black points and lines represent Pakistan, India, Nigeria, Ethiopia, and the Democratic Republic of Congo (DRC). Countries are ordered by the mean year they reach elimination, and the order will differ by scenario and model. The transparent green, tan, and grey boxes characterize countries as 2020 eliminators, elimination possible (although potentially improbable), and non-eliminators, respectively.

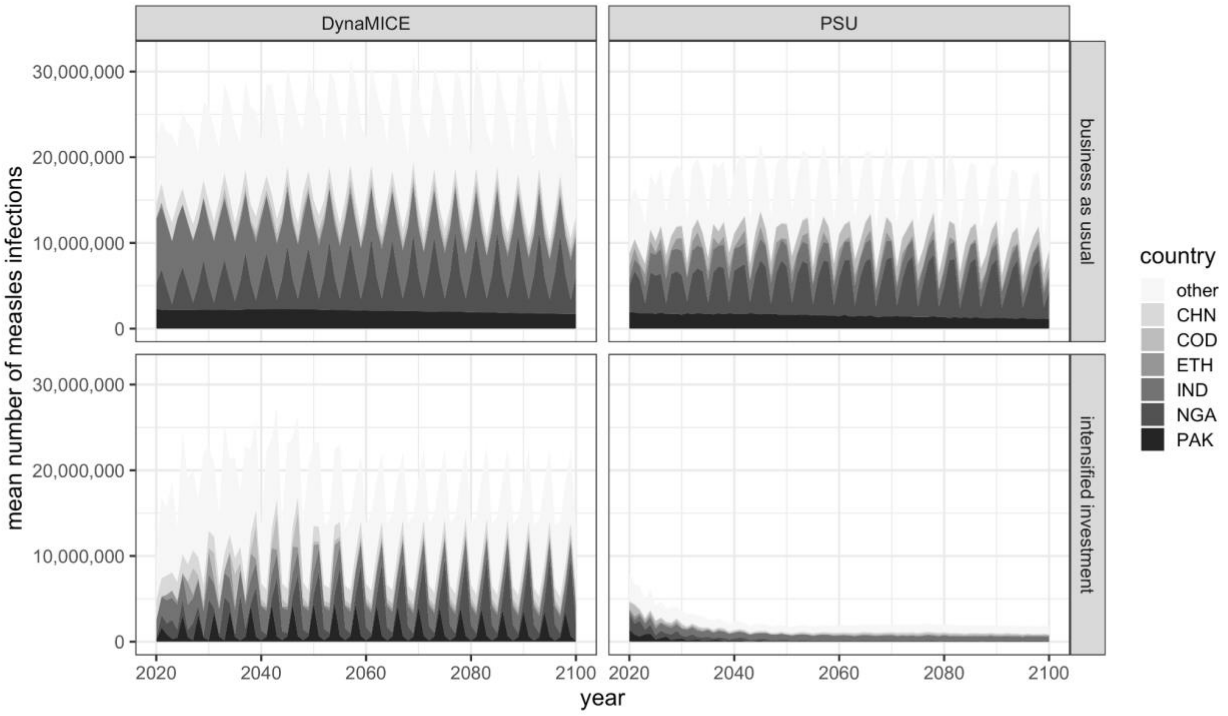

Figure S13: Annual measles infections.

The mean number of annual measles infections for China (CHN), Democratic Republic of Congo (COD), Ethiopia (ETH), India (IND), Nigeria (NGA), Pakistan (PAK), and all other countries combined (other) per DynaMICE (left column) and PSU (right column) models for business-as-usual (top row) and intensified-investment (bottom row) vaccination scenarios.

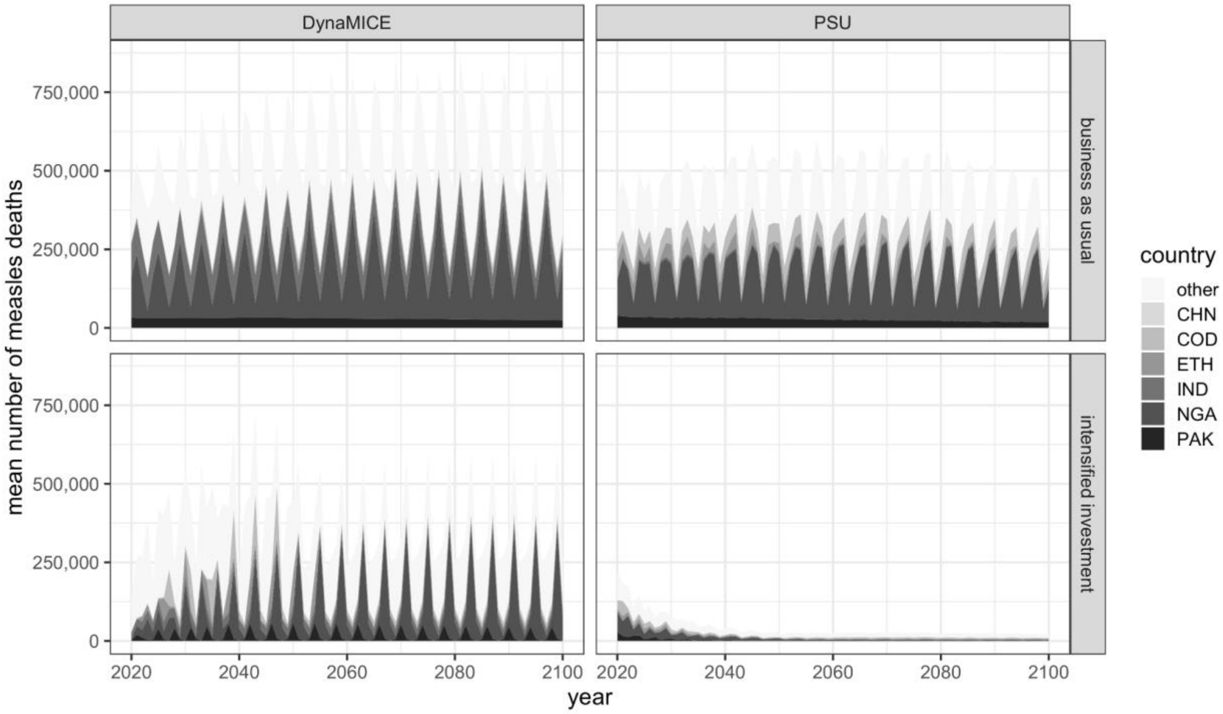

Figure S15: Annual measles deaths.

The mean number of annual measles deaths for China (CHN), Democratic Republic of Congo (COD), Ethiopia (ETH), India (IND), Nigeria (NGA), Pakistan (PAK), and all other countries combined (other) per DynaMICE (left column) and PSU (right column) models for business-as-usual (top row) and intensified-investment (bottom row) vaccination scenarios.

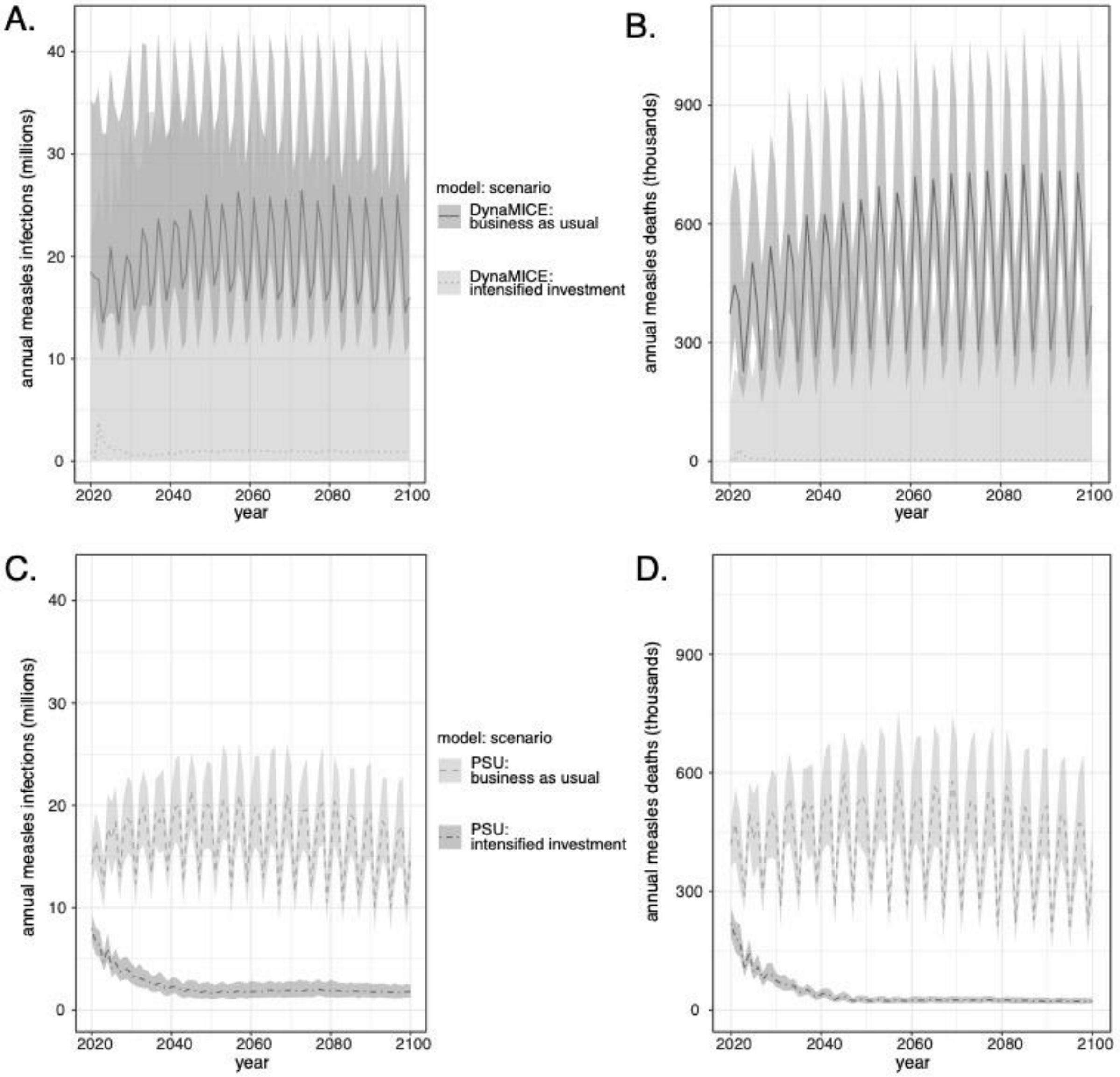

Figure S15: Measles burden with model uncertainty.

Time series of the annual aggregate number of measles infections and deaths across 93 countries based on London School of Hygiene & Topical Medicine (DynaMICE) (A,B) and Pennsylvania State University (PSU) (C,D) models given business-as-usual and intensified-investment vaccination scenarios. The line (i.e., color and line type) and ribbon (i.e., transparent shading) for each model and scenario represent the median and 80% prediction intervals across 200 stochastic runs.

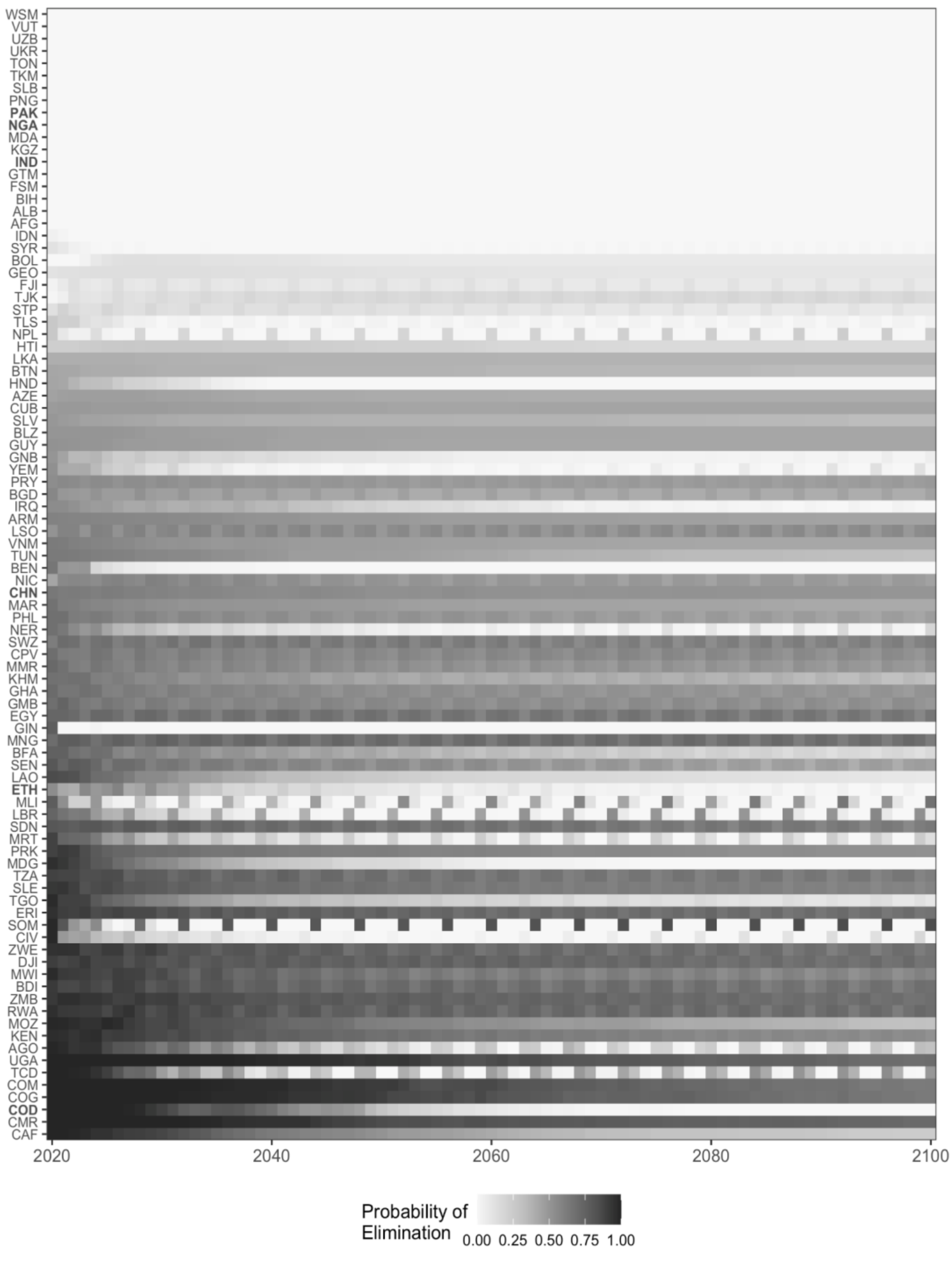

Figure S16: Probability of measles elimination, DynaMICE, business-as-usual.

DynaMICE model estimates of the annual probability of reaching the "elimination" threshold of 5 measles cases per one million population, shown as the proportion of 200 stochastic runs which reach the threshold in the **business-as-usual** vaccination scenario. Countries are listed according to their International Organization for Standardization 3166 alpha-3 country codes (ISO 3; https://www.iso.org/iso-3166-country-codes.html).

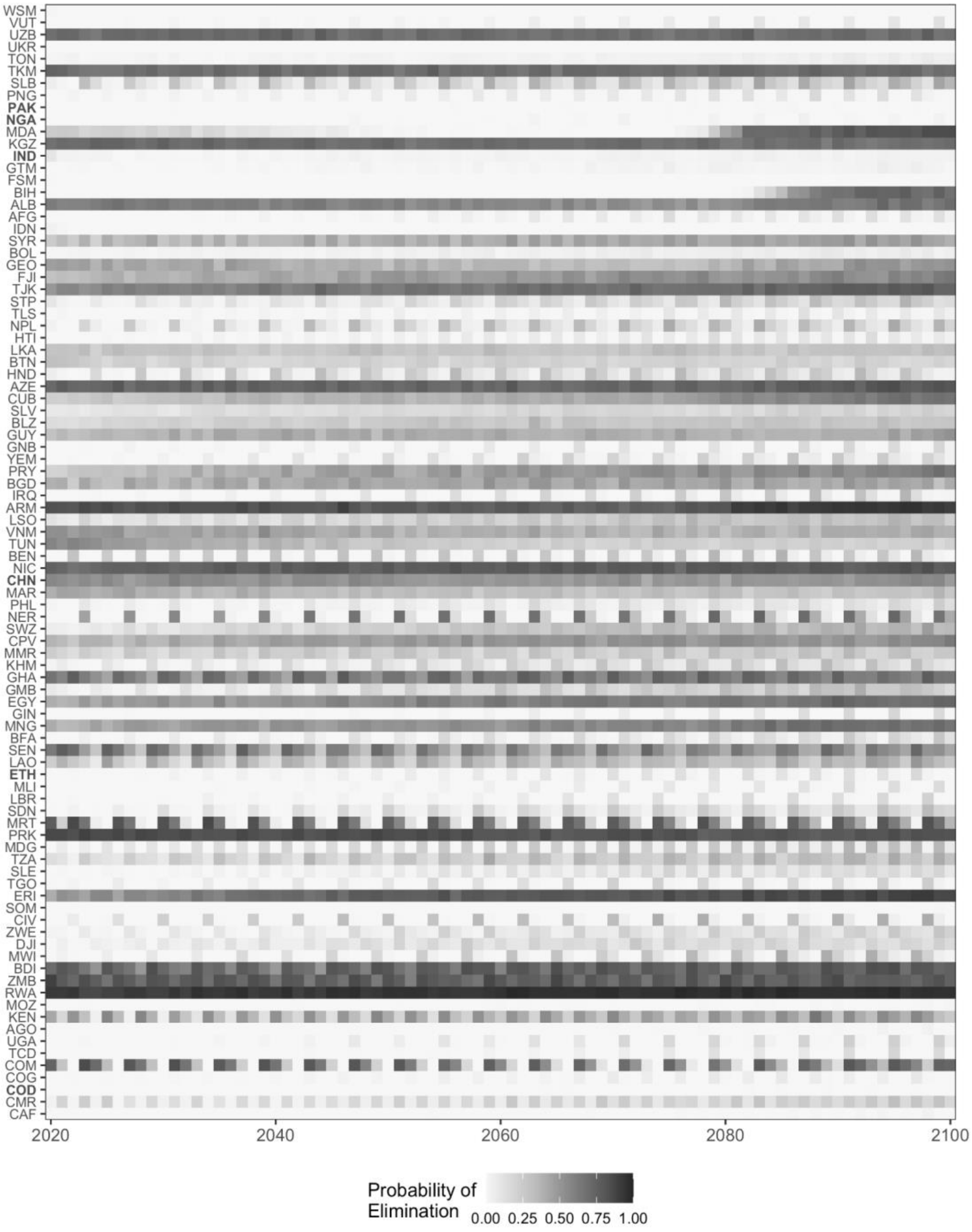

Figure S17: Probability of measles elimination, PSU, business-as-usual.

PSU model estimates of the annual probability of reaching the “elimination” threshold of 5 measles cases per one million population, shown as the proportion of 200 stochastic runs which reach the threshold in the **business-as-usual** vaccination scenario. Countries are listed according to their International Organization for Standardization 3166 alpha-3 country codes (ISO 3; https://www.iso.org/iso-3166-country-codes.html).

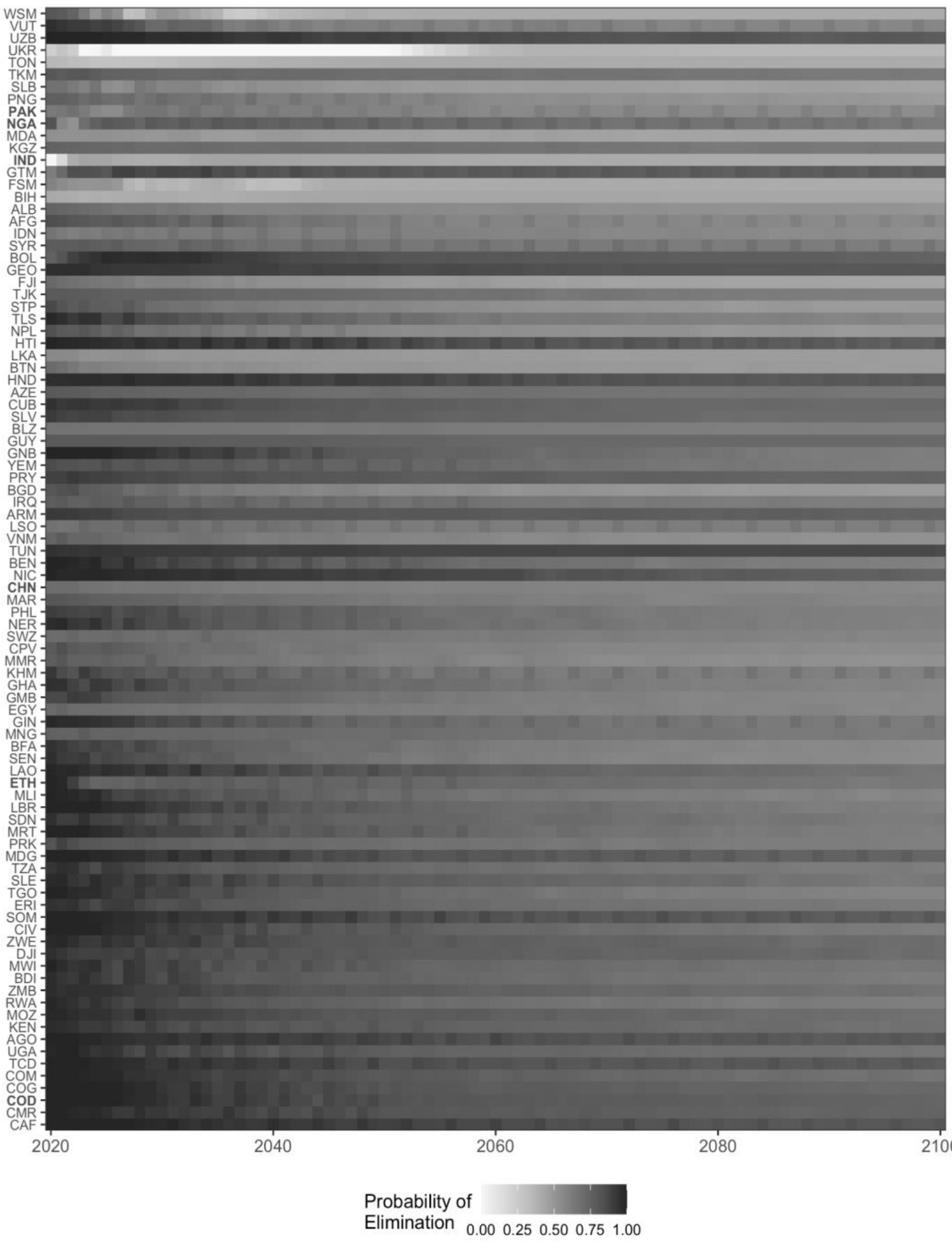

Figure S18: Probability of measles elimination, DynaMICE, intensified-investment.

DynaMICE model estimates of the annual probability of reaching the “elimination” threshold of 5 measles cases per one million population, shown as the proportion of 200 stochastic runs which reach the threshold in the **intensified-investment** vaccination scenario. Countries are listed according to their International Organization for Standardization 3166 alpha-3 country codes (ISO 3; https://www.iso.org/iso-3166-country-codes.html).

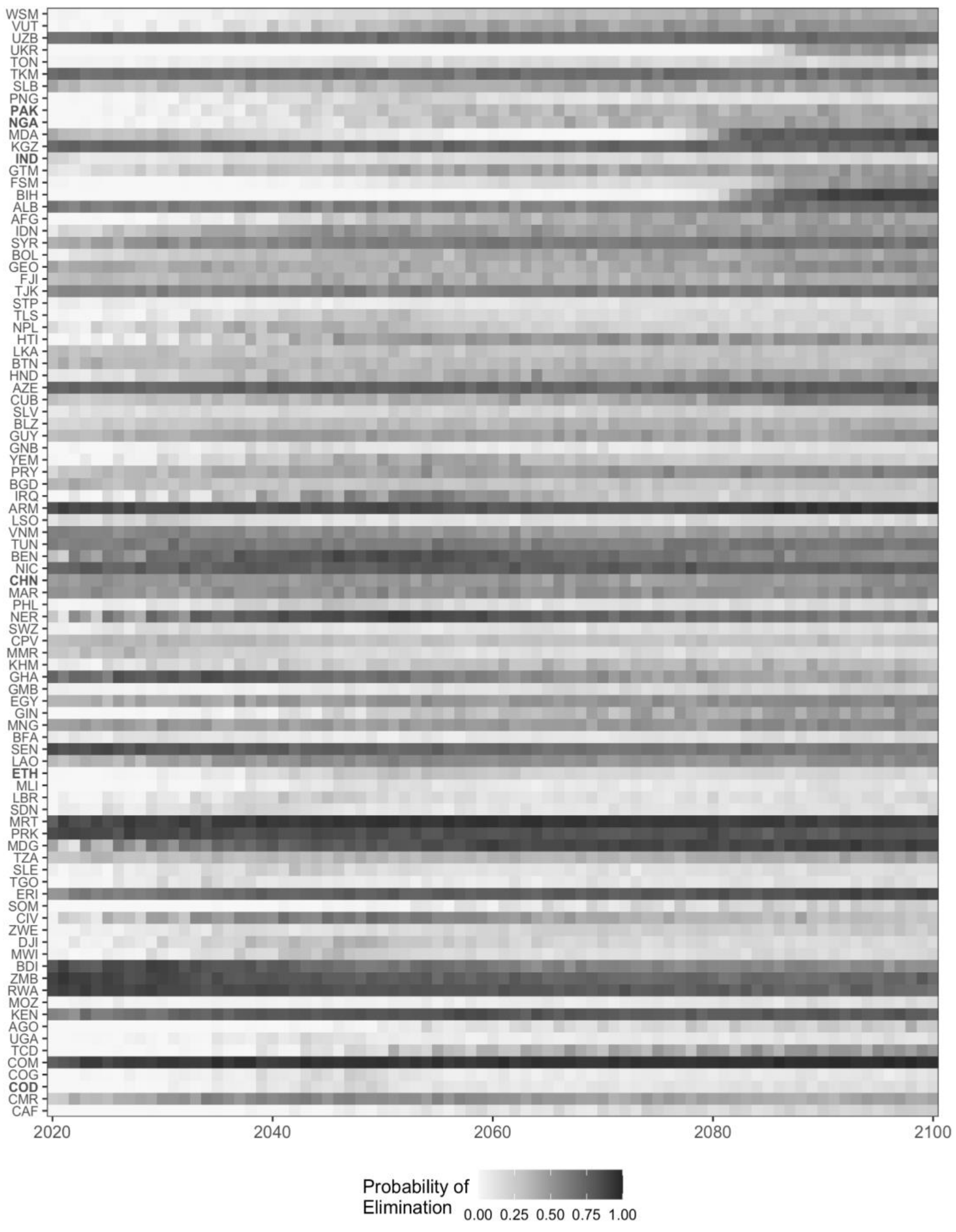

Figure S19: Probability of measles elimination, PSU, intensified-investment.

PSU model estimates of the annual probability of reaching the “elimination” threshold of 5 measles cases per one million population, shown as the proportion of 200 stochastic runs which reach the threshold in the **intensified-investment** vaccination scenario. Countries are listed according to their International Organization for Standardization 3166 alpha-3 country codes (ISO 3; https://www.iso.org/iso-3166-country-codes.html).

Measles Results - Subnational Model

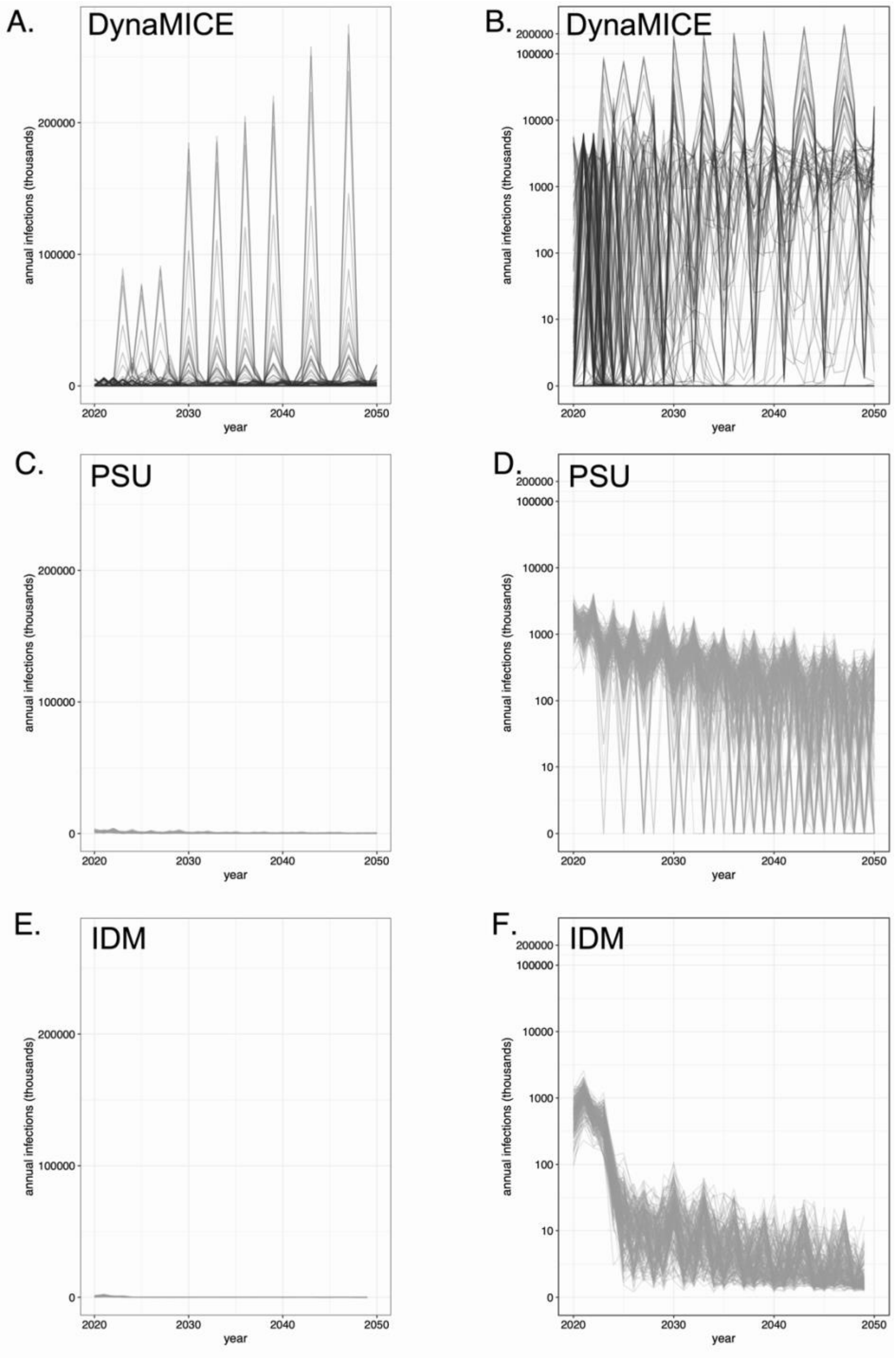

Figure S20: Time series of measles infections in Nigeria.

Time series of incident measles infections in Nigeria based on the DynaMICE national model (A,B), PSU national model (B,C), and IDM sub-national model (E, F) under the intensified-investment vaccination scenario. Each line represents one stochastic simulation, 200 total for each model. Figures B, D, and F y-axis is log base 10 annual infections in thousands with a smooth transition to linear scale around 0.

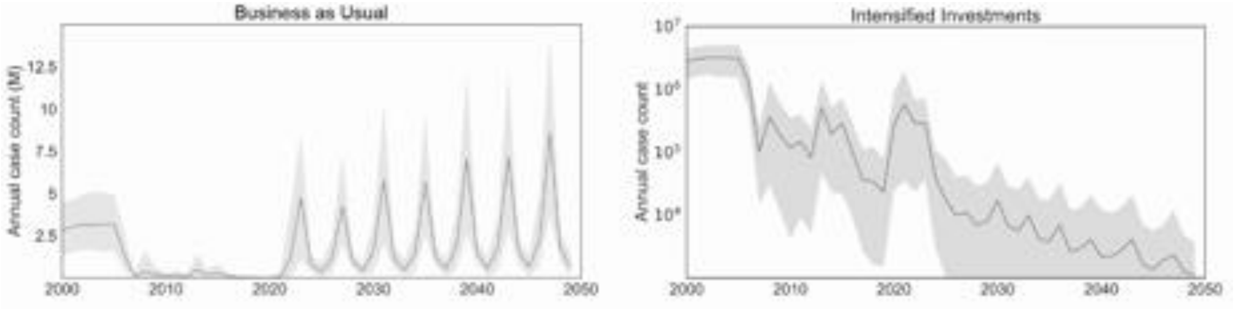

Figure S21: Annual measles infections in Nigeria, IDM.

Annual numbers of measles infections for Nigeria for the IDM sub-national model, in the **business-as-usual** (left) and **intensified-investment** (right) scenarios.

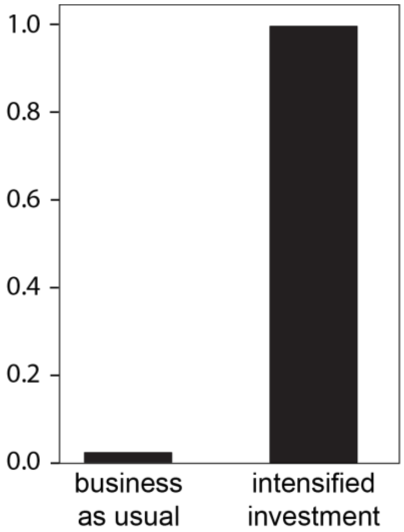

Figure S22: Probability of measles elimination in Nigeria, IDM.

Probability of reaching the "elimination" threshold of 5 measles cases per one million population, shown as the proportion of stochastic runs that reach the threshold by vaccination scenario in the IDM sub-national model.

Coverage Estimates

Vaccination coverage estimates for each country (93 total), year (1980-2100), disease (measles, rubella) and vaccine scenario (business as usual and intensified investment) can be found here: Coverage.Estimates.Appendix

Model Output

Modelled output of time-series of median estimates of measles and rubella elimination probabilities by country and vaccination scenario can be found here: Prob.Elimination.Appendix

References

1 World Population Prospects - Population Division - United Nations. https://population.un.org/wpp/ (accessed April 14, 2021).
2 WHO UNICEF coverage estimates WHO World Health Organization: Immunization, Vaccines And Biologicals. Vaccine preventable diseases Vaccines monitoring system 2020 Global Summary Reference Time Series: MCV1. https://apps.who.int/immunization_monitoring/globalsummary/timeseries/tswucoveragemcv1.html (accessed April 14, 2021).
3 Verguet S, Johri M, Morris SK, Gauvreau CL, Jha P, Jit M. Controlling measles using supplemental immunization activities: A mathematical model to inform optimal policy. *Vaccine* 2015; **33**: 1291–6.
4 Chen S, Fricks J, Ferrari MJ. Tracking measles infection through non-linear state space models. *J R Stat Soc Ser C Appl Stat* 2012; **61**: 117–34.
5 Welcome to EMOD modeling for general disease — Generic Model documentation. https://docs.idmod.org/projects/emod-generic/en/latest/ (accessed April 14, 2021).
6 Metcalf CJE, Lessler J, Klepac P, Cutts F, Grenfell BT. Impact of birth rate, seasonality and transmission rate on minimum levels of coverage needed for rubella vaccination. *Epidemiol Infect* 2012; **140**: 2290–301.
7 Metcalf CJE, Lessler J, Klepac P, Morice A, Grenfell BT, Bjørnstad ON. Structured models of infectious disease: Inference with discrete data. *Theor Popul Biol* 2012; **82**: 275–82.
8 Vynnycky E, Papadopoulos T, Angelis K. The impact of Measles-Rubella vaccination on the morbidity and mortality from Congenital Rubella Syndrome in 92 countries. *Hum Vaccines Immunother* 2019; **15**: 309–16.
9 Schenzle D. An age-structured model of pre- and post-vaccination measles transmission. *IMA J Math Appl Med Biol* 1984; **1**: 169–91.
10 Stover J, Garnett GP, Seitz S, Forsythe S. The Epidemiological Impact of an HIV/AIDS Vaccine in Developing Countries. The World Bank, 2002 DOI:10.1596/1813-9450-2811.
11 Mossong J, Hens N, Jit M, *et al.* Social Contacts and Mixing Patterns Relevant to the Spread of Infectious Diseases. *PLOS Med* 2008; **5**: e74.
12 Anderson RM, May RM. Infectious Diseases of Humans: Dynamics and Control. OUP Oxford, 1992.
13 Fu H, Abbas K, Klepac P, *et al.* Effect of evidence updates on key determinants of measles vaccination impact: a DynaMICE modelling study in ten high-burden countries. *BMC Med* 2021; **19**: 281.
14 Eilertson KE, Fricks J, Ferrari MJ. Estimation and prediction for a mechanistic model of measles transmission using particle filtering and maximum likelihood estimation. *Stat Med* 2019; **38**: 4146–58.
15 Dixon MG, Ferrari M, Antoni S, *et al.* Progress Toward Regional Measles Elimination — Worldwide, 2000–2020. *Morb Mortal Wkly Rep* 2021; **70**: 1563–9.
16 Santos H, Eilertson K, Lambert B, Hauryski S, Patel M, Ferrari M. Ensemble model estimates of the global burden of measles morbidity and mortality from 2000 to 2019: a modeling study. 2022; 10.1101/2021.08.31.21262916.
17 Wolfson LJ, Grais RF, Luquero FJ, Birmingham ME, Strebel PM. Estimates of measles case fatality ratios: a comprehensive review of community-based studies†. *Int J Epidemiol* 2009; **38**: 192–205.
18 Zimmermann M, Frey K, Hagedorn B, *et al.* Optimization of frequency and targeting of measles supplemental immunization activities in Nigeria: A cost-effectiveness analysis. *Vaccine* 2019; **37**: 6039–47.
19 The DHS Program - Quality information to plan, monitor and improve population, health, and nutrition programs. https://dhsprogram.com/ (accessed April 14, 2021).
20 Leuridan E, Hens N, Hutse V, Ieven M, Aerts M, Van Damme P. Early waning of maternal measles antibodies in era of measles elimination: longitudinal study. *BMJ* 2010; **340**: c1626.
21 Gaythorpe KA, Abbas K, Huber J, *et al.* Impact of COVID-19-related disruptions to measles, meningococcal A, and yellow fever vaccination in 10 countries. *eLife* 2021; **10**: e67023.

22 Vynnycky E, Yoshida LM, Huyen DTT, *et al.* Modeling the impact of rubella vaccination in Vietnam. *Hum Vaccines Immunother* 2016; **12**: 150–8.
23 Vynnycky E, Adams EJ, Cutts FT, *et al.* Using Seroprevalence and Immunisation Coverage Data to Estimate the Global Burden of Congenital Rubella Syndrome, 1996-2010: A Systematic Review. *PLOS ONE* 2016; **11**: e0149160.
24 Vynnycky E, Gay NJ, Cutts FT. The predicted impact of private sector MMR vaccination on the burden of Congenital Rubella Syndrome. *Vaccine* 2003; **21**: 2708–19.
25 Simons EA, Reef SE, Cooper LZ, Zimmerman L, Thompson KM. Systematic Review of the Manifestations of Congenital Rubella Syndrome in Infants and Characterization of Disability-Adjusted Life Years (DALYs). *Risk Anal* 2016; **36**: 1332–56.
26 Data Catalog | Data Catalog. https://datacatalog.worldbank.org/ (accessed April 14, 2021).
27 Miller E, Cradock-Watson JE, Pollock TM. Consequences of confirmed maternal rubella at successive stages of pregnancy. *Lancet* 1982; **320**: 781-4.
28 Grillner L, Forsgren M, Barr B, Bottiger M, Danielsson L, De Verdier C. Outcome of rubella during pregnancy with special reference to the 17th-24th weeks of gestation. *Scand J Infect Dis*. 1983; **15**: 321-5.
29 Hahne S, Macey J, van Binnendijk R, Kohl R, Dolman S, van der Veen Y, et al. Rubella outbreak in the Netherlands, 2004-2005: high burden of congenital infection and spread to Canada. *Pediatr Infect Dis J*. 2009; **28**: 795-800.
30 Toizumi M, Motomura H, Vo HM, Takahashi K, Pham E, Nguyen HA, et al. Mortality associated with pulmonary hypertension in congenital rubella syndrome. *Pediatrics*. 2014; **134**: e519-26.
31 Panagiotopoulos T, Georgakopoulou T. Epidemiology of rubella and congenital rubella syndrome in Greece, 1994-2003. *Euro Surveill*. 2004; **9**: 17-9.
32 Saad de Owens C, Tristan de Espino R. Rubella in Panama: still a problem. *Pediatr Infect Dis J*. 1989; **8**: 110-5.
33 Nicoara C, Zäch K, Trachsel D, Germann D, Matter L. Decay of Passively Acquired Maternal Antibodies against Measles, Mumps, and Rubella Viruses. *Clin Diagn Lab Immunol* 1999; **6**: 868–71.
34 Boulianne N, De Serres G, Ratnam S, Ward BJ, Joly JR, Duval B. Measles, mumps, and rubella antibodies in children 5–6 years after immunization: effect of vaccine type and age at vaccination. *Vaccine* 1995; **13**: 1611–6.
35 Prem K, Cook AR, Jit M. Projecting social contact matrices in 152 countries using contact surveys and demographic data. *PLOS Comput Biol* 2017; **13**: e1005697.
36 Farrington CP. Modelling forces of infection for measles, mumps and rubella. *Stat Med* 1990; **9**: 953–67.
37 Enders G, Miller E, Nickerl-Pacher U, Cradock-Watson JohnE. Outcome of confirmed periconceptional maternal rubella. *Lancet* 1988; **331**: 1445–7.
38 Ghidini A, Lynch L. Prenatal diagnosis and significance of fetal infections. *West J Med* 1993; **159**: 366–73.
39 Munro ND, Smithells RW, Sheppard S, Holzel H, Jones G. Temporal relations between maternal rubella and congenital defects. *Lancet* 1987; **330**: 201–4.
40 Siegel M, Fuerst HT, Peress NS. Comparative Fetal Mortality in Maternal Virus Diseases. *N Engl J Med* 1966; **274**: 768–71.
41 Winter AK, Pramanik S, Lessler J, Ferrari M, Grenfell BT, Metcalf CJE. Rubella vaccination in India: identifying broad consequences of vaccine introduction and key knowledge gaps. *Epidemiol Infect* 2018; **146**: 65–77.
